# **α**7nAChR on B cells directs T cell differentiation to prevent viral myocarditis

**DOI:** 10.1172/jci.insight.189323

**Published:** 2025-05-08

**Authors:** Jing Lu, Keren Chen, Zhihong Cen, Yanlan Huang, Yong Li, LiLi Chen, Weifeng Wu

**Affiliations:** 1 Department of Cardiology, and; 2 Emergency Department, The First Affiliated Hospital of Guangxi Medical University, Nanning, Guangxi Zhuang Autonomous Region, China.; 3 Collaborative Innovation Centre of Regenerative Medicine and Medical BioResource Development and Application Co. constructed by the Province and Ministry, Guangxi Medical University, Nanning, Guangxi Zhuang Autonomous Region, China.

**Keywords:** Cardiology, Inflammation, Cardiovascular disease, Cellular immune response, Heart failure

## Abstract

Patients with viral myocarditis (VMC) exhibit evident autonomic nervous system imbalance, and adverse cardiac remodeling is involved in impaired cholinergic function. The α7 nicotinic acetylcholine receptor (α7nAChR), which is a neurotransmitter receptor, exerts immunoregulatory effects. Recent advances have illuminated the evolution and functions of peripheral and cardiac B cells in heart disease. However, the role of α7nAChR expressed by B cells in the progression of VMC has not been established. We revealed the neuroimmune communication landscape in the heart and found that the phenotypes of cardiac and splenic B cells and their α7nAChR expression changed dynamically during the progression of VMC to dilated cardiomyopathy. α7nAChR on B cells serves as a negative regulator by inhibiting their proinflammatory functions and signaling pathways. B cell–specific α7nAChR deficiency exacerbated myocardial inflammation, fibrosis, and cardiac dysfunction. However, these effects were abrogated in non-B cells from mice with IL-17A knockdown. Enhanced degradation of acetylcholine leads to an imbalance in cholinergic signaling, resulting in impaired neurotransmission. The acetylcholinesterase inhibitor pyridostigmine bromide could improve cardiac remodeling and prevent the progression of VMC to the chronic phase, which was partly dependent on the α7nAChR on B cells. Our findings provide notable insights into cardiac-neural-immune communication during myocardial injury.

## Abstract 2

Impaired activation and dysfunction of the cholinergic system occur in the progression of VMC.The restoration of cholinergic activity by pyridostigmine bromide significantly improves myocardial injury and remodeling, which involves the regulatory role of α7nAChR on B cells in Th17 cell differentiation.

## Graphical abstract

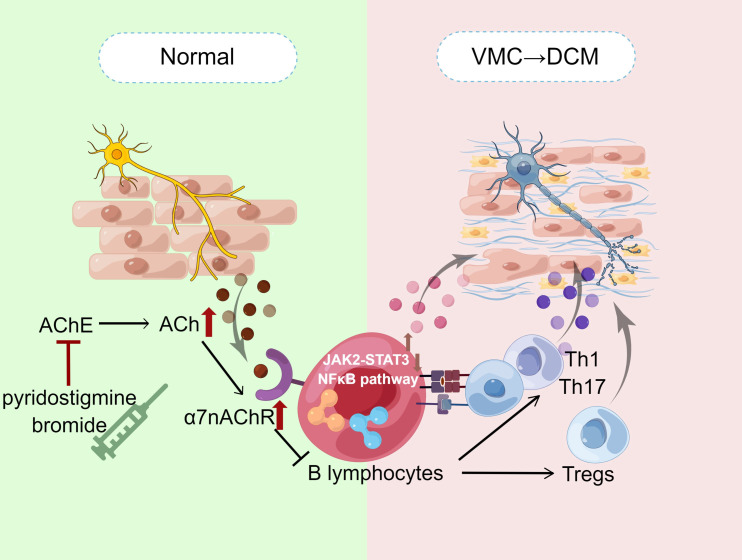

## Introduction

Viral myocarditis (VMC) is an inflammatory heart disease caused by viral infection, with coxsackievirus B3 (CVB3) being the most common pathogen (1). Some patients with VMC can resolve acute inflammation and self-heal without specific treatment, whereas others exhibit persistent inflammation and progress to chronic myocarditis or even inflammatory dilated cardiomyopathy (DCM) (2). The viral infection invades the myocardial tissue, and the excessive and prolonged activation of the antiviral immune response further aggravates cardiac inflammation and initiates a CD4^+^ T cell–mediated autoimmune response, leading to the progression of VMC (3). An increased percentage of T helper 17 (Th17) cells and greater levels of IL-17–promoting cytokines in the peripheral blood are observed in patients with myocarditis/DCM with severe heart failure and myocardial fibrosis (4). Neutralization or genetic knockout of IL-17 can substantially reduce myocardial inflammation and fibrosis and improve cardiac function (5). Thus, timely restraint of Th cell immune responses is essential for alleviating myocardial inflammation and inhibiting progression to the chronic phase of VMC.

B cells, as antigen-presenting cells (APCs), play crucial roles in the activation and differentiation of T cells in the heart inflammatory response. Cardiac B cells constitute the second largest population of leukocytes after macrophages in normal mouse hearts (6, 7). B cells play pivotal roles in inflammatory heart disease through humoral immunity and nonclassical functions independent of antibodies. Cardiac B cells produce chemotactic signals to regulate the mobilization of local innate and adaptive immune cells toward the heart (8). For example, pericardial B cells can recruit dendritic cells through the GM-CSF/CCR7 axis, promote T cell expansion in pericardial adipose tissue bone marrow (BM) granulocyte production, and reduce neutrophil infiltration in the heart (9). Adoptive transfer of regulatory B (Breg) cells markedly reduces the number of Ly6C^hi^ monocytes in the heart through the secretion of IL-10 and by reducing C-C chemokine receptor 2 (CCR2) expression in monocytes (10, 11). We have identified dual roles of B cells in VMC through the modulation of Th cell differentiation. B cells play a pathogenic role in VMC by promoting Th1 and Th17 cell differentiation while inhibiting Th2 cell differentiation (12). However, the absence of B cells reduces the number of splenic and myocardial regulatory T (Treg) cells, impairing their activation and antiinflammatory functions (13). Additionally, adoptive transfer of Breg cells can prevent a shift in the Th17/Treg balance toward Th17 cells, thereby alleviating myocardial inflammation (14). The potential beneficial effects of B cells should not be neglected. Specific targeted therapy that can coordinate B cell immune function is a potential alternative strategy to conventional nonspecific B cell depletion treatment to prevent the progression of VMC to the chronic phase.

Significant autonomic nervous system dysfunction is observed in patients with VMC and DCM (15–17), which is closely associated with the cardiac inflammatory response (18). The cholinergic signaling pathway within the heart mediates the involvement of immune cells in the local immune response in the heart. For example, pyridostigmine bromide (PYR) can reduce the chemotactic factor CCL2/7 (C-C chemokine ligand), leading to a reduction in cardiac MHC-II^hi^CCR2^+^ macrophage infiltration and increases in the numbers of M2 macrophages and Treg cells in mouse models of myocardial infarction (MI) (19–21). The α7 nicotinic acetylcholine receptor (α7nAChR) is a neurotransmitter receptor that mediates neuroimmune crosstalk, especially the cholinergic antiinflammatory pathway, which is a communication circuit connecting the brain and spleen (22). Multiple nAChR subtypes are present on murine B cells, and activated B cells upregulate α4β2 and α7nAChRs (23–25). α7nAChR promotes the proliferation and differentiation of B cells in the BM (26) and inhibits the proliferative function of mature B cells stimulated by anti-CD40 in the spleen (25). The absence of α7nAChR increases peripheral IgG1 levels and decreases the expression of Foxp3 and IL-10 in B cells (27, 28), but it does not affect immunoglobulin class switching (IgM-IgG) (25). Therefore, α7nAChR plays a regulatory role in the proliferation and antibody production of B cells in the spleen. However, the mechanisms underlying the impact of α7nAChR expressed by B cell subsets and nonclassical functions independent of antibodies on myocardial inflammation remain unclear.

Acetylcholine (ACh) in the heart is derived from neuronal (vagus) and nonneuronal (including cardiomyocytes, endothelial, and immune) cells (29). Enhancing cholinergic activity by vagus nerve stimulation and cholinesterase inhibitors has been shown to improve survival, cardiac function, and adverse remodeling in MI, heart failure, and other mouse models (30–32). This approach also holds promise for improving patient outcomes and quality of life (33). Although the majority of the cardioprotective effects of cholinergic activity have been attributed to hemodynamic improvements, the inhibition of oxidative stress and the promotion of angiogenesis and cholinergic signaling also play beneficial immunomodulatory roles by influencing the recruitment and function of cardiac immune cells (34, 35). In VMC, the precise cellular targets and mechanisms of the antiinflammatory effects of ACh signaling within the heart in vivo remain unknown. Here, we showed that ACh signaling exerts unexpectedly beneficial effects on VMC progression through a mechanism that involves α7nAChR on B cells.

## Results

### Landscape characteristics of immune infiltration and cholinergic components in VMC mouse hearts.

**Figure 1:**
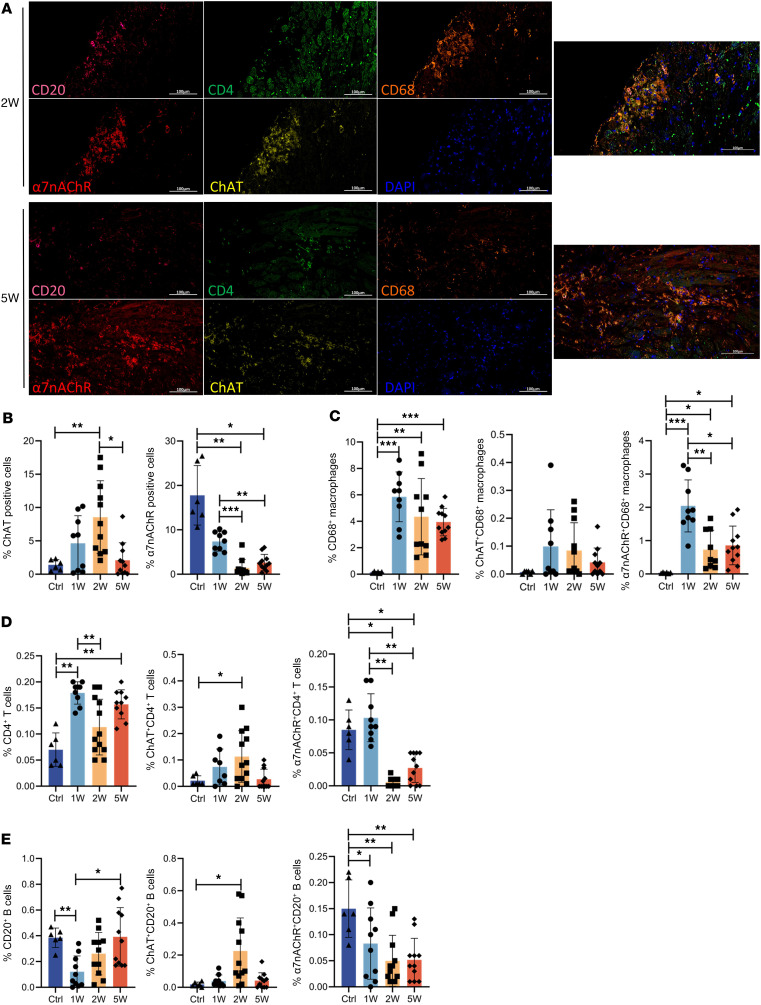
Landscape of relationship between cholinergic components and immune characteristics. (**A**) Multiplex staining was performed on transverse sections of the left ventricles of VMC mice on weeks 1, 2, and 5 after infection with CVB3. Tissue sections were imaged at ×200 magnification. Scale bars: 100 μm. CD4, green; CD68, orange; α7nAChR, red; ChAT, yellow; CD20, pink; DAPI, blue. (**B**–**E**) Quantification of the number of (**B**) ChAT^+^ and α7nAChR^+^ cells; (**C**) CD68^+^, CD68^+^α7nAChR^+^ double-positive, and CD68^+^ChAT^+^ double-positive cells; (**D**) CD4^+^, CD4^+^α7nAChR^+^ double-positive, and CD4^+^ChAT^+^ double-positive cells; and (**E**) CD20^+^, CD20^+^α7nAChR^+^ double-positive, and CD20^+^ChAT^+^ double-positive cells relative to the number of total cells (*n* = 6–12 mice/group). The data were analyzed using 1-way ANOVA and are represented as the mean ± SD. *P < 0.05; **P < 0.01; ***P < 0.001 between groups.

Research has demonstrated that various cell types, including cardiomyocytes, fibroblasts, and immune cells, can express nonneuronal components of the cholinergic system. For example, cardiomyocytes can secrete ACh to alleviate myocardial remodeling (36). Additionally, splenic T cells expressing choline acetyltransferase (ChAT) secrete ACh, which acts upon the α7nAChR on macrophages or the α9nAChR on B cells, thereby forming a neuroimmune axis (22, 37). In a preliminary characterization of the neuroimmune communication landscape and relationship in the heart, we conducted multiplex immunohistochemical analysis of CD20^+^ (B cells), CD4^+^ (T cells), CD68^+^ (macrophages), ChAT^+^ (ACh-synthesizing), and α7nAChR^+^ cells in the hearts of VMC mice at 1 week (acute phase), 2 weeks (peak of acute inflammation), and 5 weeks (chronic phase) (Figure 1A).

Numerous α7nAChR^+^ cells and ChAT^+^ cells were observed in the heart tissue. The total number of α7nAChR^+^ cells decreased significantly after CVB3 infection, whereas the total number of ChAT^+^ cells increased significantly during the acute phase and gradually decreased thereafter (Figure 1B). In addition to the postganglionic vagus nerve terminals, ChAT^+^ cells in the ventricles also include nonneuronal cells such as cardiomyocytes and immune cells. We further analyzed the correlations between immune cells and ChAT and α7nAChR. Abundant macrophages were distributed in focal or diffuse myocardial interstitial inflammatory infiltrates, whereas T and B cells were more common in the epicardium. CD68^+^ macrophages exhibited a sustained increase in the innate immune response throughout the progression of VMC and expressed elevated levels of α7nAChR, with the highest level occurring at week 1 (Figure 1C). With respect to adaptive immunity, T and B cells presented marked decreases in α7nAChR expression at weeks 1 and 2, whereas the expression of ChAT increased (Figure 1, D and E). α7nAChR on T cells has been found to act as a relay station for the regulation of immune responses in macrophages and B cells (22, 37), whereas the role of α7nAChR on B cells in immune crosstalk has been less well studied.

### The α7nAChR exhibits distinct changes within the cardiac and splenic B cell subpopulations, potentially affecting the intensity of immune responses.

**Figure 2:**
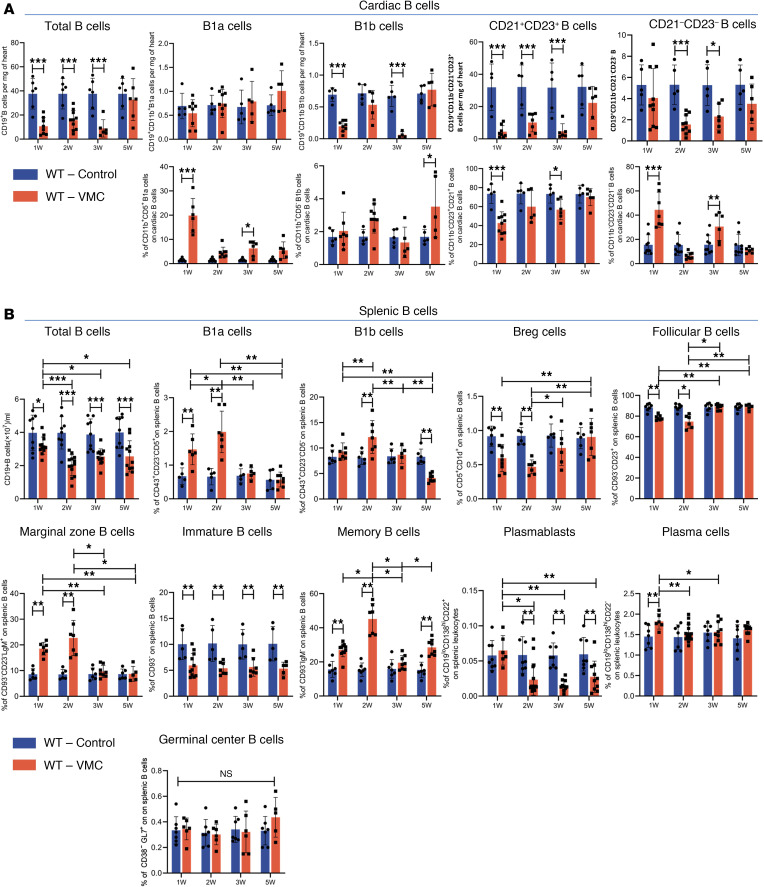
Distribution of main B cell subsets in spleen and heart during VMC progression. (**A**) Summary graphs showing the number of total infiltrating B cells among gated leukocytes in myocardial tissue, as well as the proportions of cardiac B cell subsets (B1a cells, B1b cells, CD21^+^CD23^+^ B cells, and CD21^–^CD23^–^ B cells) among gated cardiac B cells (*n* = 5–11 mice/group). (**B**) The frequencies of main B cell subsets in the spleen of VMC and control group (B1a cells, B1b cells, Breg cells, immature B cells, marginal zone B cells, follicular B cells, memory B cells, plasmablasts, plasma cells, and germinal center B cells) were analyzed by flow cytometry (*n* = 5–17 mice/group). The data were analyzed using 1-way ANOVA and are represented as the mean ± SD. *P < 0.05; **P < 0.01; ***P < 0.001 between groups.

B cells include various subsets with heterogeneous functions. Several studies have reported disturbances in B cell homeostasis, particularly the distribution of B cell subsets, in a variety of diseases (38). We investigated whether these abnormalities were present in the VMC mouse model and, if so, how their α7nAChR expression changed as the disease progressed to the chronic phase. The gating strategy for splenic and cardiac B cells for FACS analysis is shown in Supplemental Figure 1A and Supplemental Table 1 (supplemental material available online with this article; https://doi.org/10.1172/jci.insight.189323DS1) based on previous studies (39–41). The total cardiac B cell count and each subset initially decreased and then tended to increase during the progression of VMC to the chronic stage (Figure 2A). Cardiac B cells can be divided into 4 subgroups: B1 cells (CD11b^+^CD5^+^ B1a cells and CD11b^+^CD5^–^ B1b cells) and B2 cells (CD11b^–^CD21^+^CD23^+^ B cells and CD11b^–^CD21^–^CD23^–^ B cells) (41). We observed that the frequencies of cardiac B1a and CD11b^–^CD21^–^CD23^–^ B cells among total cardiac B cells increased significantly, whereas the frequency of CD21^+^CD23^+^ B cells decreased significantly at week 1 and week 3 (Figure 2A).

The frequency of splenic B1a cells and B1b cells increased significantly in the acute stage of VMC and peaked at week 2, whereas the frequency of splenic Breg cells decreased significantly. During the chronic stage of VMC, the frequency of Breg cells recovered, whereas the frequency of B1b cells decreased significantly at week 5 (Figure 2B). Among immature B cell subsets, there was a sustained decrease throughout VMC. Among B2 cells, marginal zone (MZ) B cells and memory B cells tended to increase in the acute phase and peaked at week 2, whereas the opposite effect was observed in follicular (FO) B cells. In the chronic stage, the frequency of memory B cells increased significantly at week 5 (Figure 2B). Among antibody-secreting cells and precursor cells, a marked increase in the plasma cell subset was noted at week 1, and plasmablast counts significantly decreased after week 2 (Figure 2B). Overall, substantial depletion of total B cells and the expansion of B1b cells and CD21^–^CD23^–^ B cells are characteristic of cardiac B cell disturbances during VMC development. The dynamic distribution of splenic B cell subsets in VMC was characterized by an overall shift to the terminal stage of differentiation.

**Figure 3:**
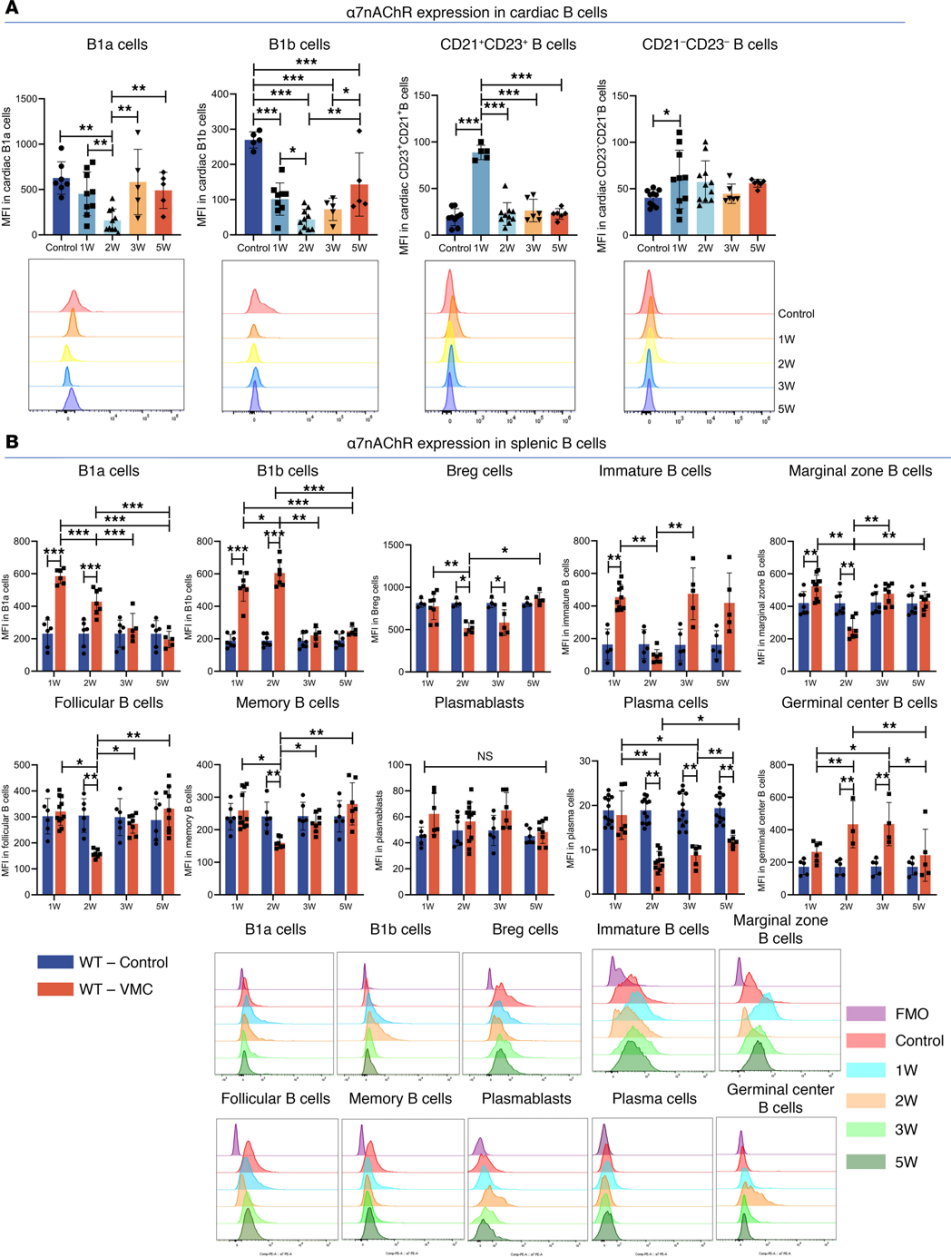
α7nAChR expression was significantly altered in cardiac and splenic B cell subpopulations. (**A**) Summary graph for mean fluorescence intensity (MFI) of α7nAChR on cardiac B cell subsets in VMC are shown (*n* = at least 5 mice/group). Representative histograms showing the MFI of α7nAChR on different cardiac B cell subsets. (**B**) Representative histograms and statistical graphs showing the MFI of α7nAChR expressed in splenic B cell subsets (B1a cells, B1b cells, Breg cells, immature B cells, marginal zone B cells, follicular B cells, memory B cells, plasmablasts, plasma cells, and germinal center B cells) over time in VMC (*n* = at least 5 mice/group). FMO, fluorescence minus one. The data are represented as the mean ± SD. *P < 0.05; **P < 0.01; ***P < 0.001 by 1-way ANOVA.

Changes in the distribution of B cell subpopulations over time may provide clues to the mechanism of α7nAChR activity on B cells. We next examined changes in α7nAChR expression levels in different subsets of B cells in the heart and spleen. Compared with those in the control group, there were substantial decreases in α7nAChR expression in cardiac B1a and B1b cells, whereas the expression levels of α7nAChR in cardiac CD23^+^CD21^+^ B cells and CD23^–^CD21^–^ B cells were increased at week 1 (Figure 3A). During the acute stage of VMC (weeks 1 and 2), α7nAChR expression levels were significantly increased in B1a cells, B1b cells, and immature B cells in the spleen but decreased in Breg cells. In the subacute and chronic stages (3 weeks and 5 weeks), α7nAChR expression in splenic B1a cells and B1b cells did not differ significantly from that in the control group but remained low in Breg cells (Figure 3B). Among B2 cells, there were strong trends toward decreased α7nAChR expression in splenic MZ B cells, FO B cells, and memory B cells at week 2 (Figure 3B). In addition, splenic germinal center B cells presented increased expression of α7nAChR after the acute phase, whereas plasma cells presented the opposite effect at weeks 2 and 3 (Figure 3B). In conclusion, the change in α7nAChR expression parallels the frequencies of certain splenic B cell subsets, specifically B1a, B1b, Breg, and FO B cells. However, the expression of α7nAChR decreased in expanded MZ B cells, memory B cells, and plasma cells.

### α7nAChR deletion in B cells promotes Th17 differentiation and impairs Treg differentiation.

**Figure 4:**
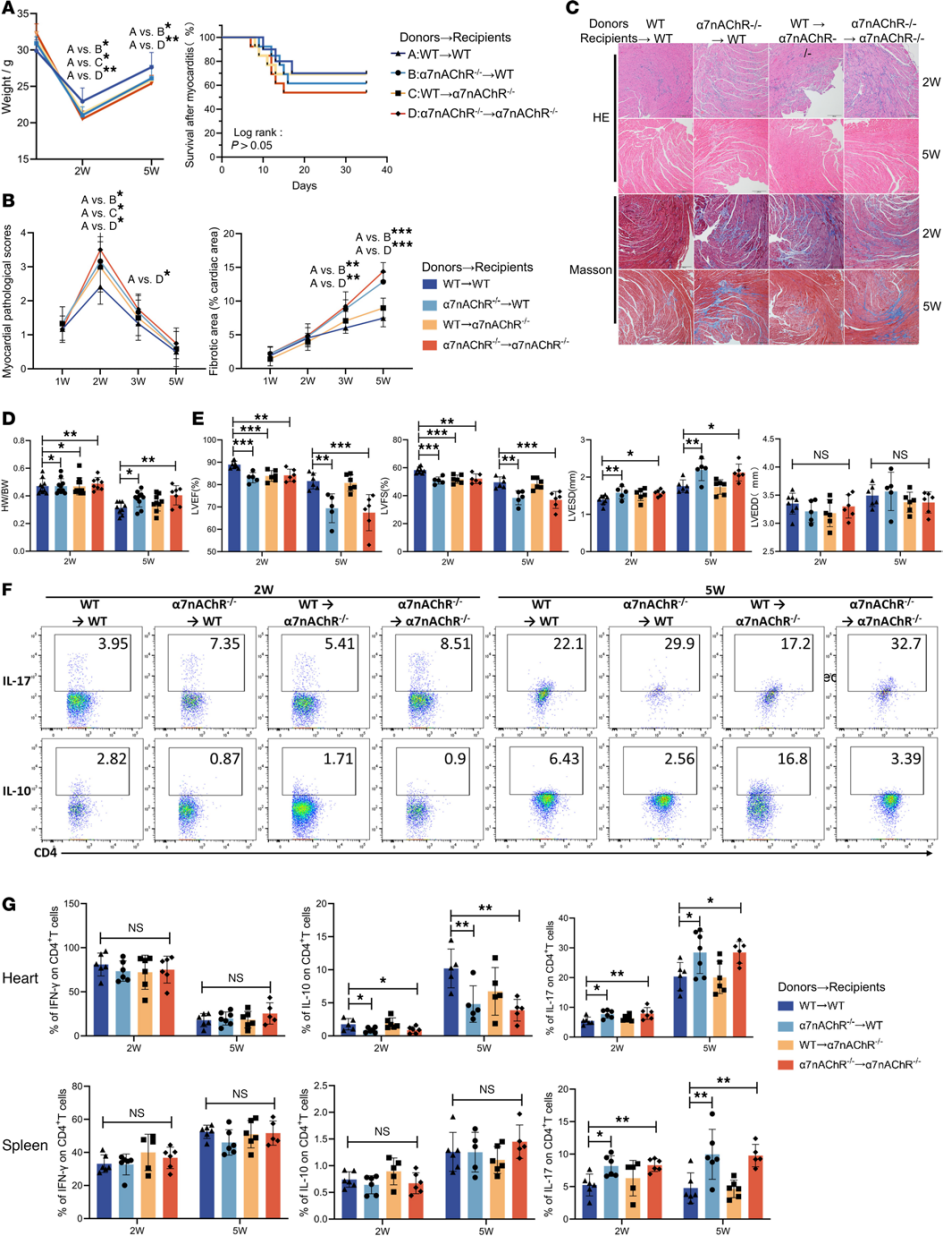
α7nAChR deficiency in hematopoietic cells reduces VMC severity in a T cell–dependent manner. Lethally irradiated WT and α7nAChR^−/−^ mice were transplanted with 1 × 10^7^ WT or α7nAChR^−/−^BM cells and chimeras were subjected to the VMC model after 4 weeks of transplantation. At 2 and 5 weeks after immunization, chimeric mice underwent echocardiography and were sacrificed. (**A**) The body weight loss and survival of each group were monitored over time. (**B**) Quantification of myocardial pathological score and the percentage of the fibrotic area are shown (*n* = 8 mice/group). (**C**) Representative images of H&E and Masson’s trichrome staining of heart sections in chimeric mice at weeks 2 and 5. Original magnification, ×40. Scale bars: 200 μm. (**D**) The heart weight/body weight (HW/BW) ratio of chimeric mice was measured (*n* = 5–11 mice/group). (**E**) The left ventricular ejection fraction (LVEF), left ventricular fractional shortening (LVFS), left ventricular end-diastolic diameter (LVEDD), and left ventricular end-systolic diameter (LVESD) of chimeric mice were determined at the endpoint (*n* = 5–7 mice/group). Representative flow cytometric plots (**F**) and flow cytometric analysis (**G**) of frequency of IFN-γ^+^, IL-10^+^, and IL-17^+^ cells among CD4^+^ T cells in the heart and spleen of VMC chimeric mice at 2 and 5 weeks after infection are shown (*n* = 5–7 mice/group). The data are represented as the mean ± SD. *P < 0.05; **P < 0.01; ***P < 0.001 by 1-way ANOVA (**A** [left], **B**, **D**, **E**, and **G**) or log-rank (Mantel-Cox) test (**A** [right]).

To determine the in vivo functions of α7nAChR, we generated α7nAChR gene–knockout mice and confirmed the knockdown efficiency by PCR and Western blotting (Supplemental Figure 1, B and C). First, we found that the systemic knockout of α7nAChR does not affect the abundance of CVB3 in myocardial tissue during the acute phase of VMC (Supplemental Figure 1D). Given that α7nAChR is expressed by nonimmune cells such as cardiomyocytes and immune cells, we used BM chimeras to distinguish the direct effects of α7nAChR on hematopoietic cells from the indirect effects mediated by nonhematopoietic cells. To exclude the effects of BM reconstruction itself, syngeneic transfers were conducted as controls. We examined the effects of specific α7nAChR deficiency on the T cell repertoire and the progression of VMC. PCR genotyping revealed that the majority of BM cells, PBMCs, and splenocytes from chimeric mice originated from wild-type (WT) or α7nAChR^–/–^ donors, providing evidence of successful BM transplantation (Supplemental Figure 1F). Chimeras lacking α7nAChR in hematopoietic or nonhematopoietic cells exhibited exacerbated cardiac inflammation (Figure 4, A–E). Regardless of the genotype of the BM recipients, chimeras lacking α7nAChR on hematopoietic cells were susceptible to more severe myocardial fibrosis and cardiac dysfunction (Figure 4, A–E). Consistent with this observation, compared with WT allogeneic transfer controls, chimeras lacking α7nAChR in the hematopoietic compartment presented substantial increases in the number and proportion of cardiac and splenic Th17 cells at week 2 and week 5; however, α7nAChR deficiency in hematopoietic cells reduced the number of Treg cells in the heart (Figure 4, F and G, and Supplemental Figure 1G). The gating strategy for flow cytometric analysis of Th cells is illustrated in Supplemental Figure 1E. There was no significant difference in the number or proportion of Th1 cells or the proliferation of Th cells among the groups (Figure 4, F and G, and Supplemental Figure 1G). These findings suggest that α7nAChR signaling in hematopoietic and nonhematopoietic cells can improve myocardial inflammation. In addition, α7nAChR expressed by hematopoietic cells inhibited the shift in the Th17/Treg balance toward the Th17 cell lineage and prevented the progression from VMC to the chronic phase.

**Figure 5:**
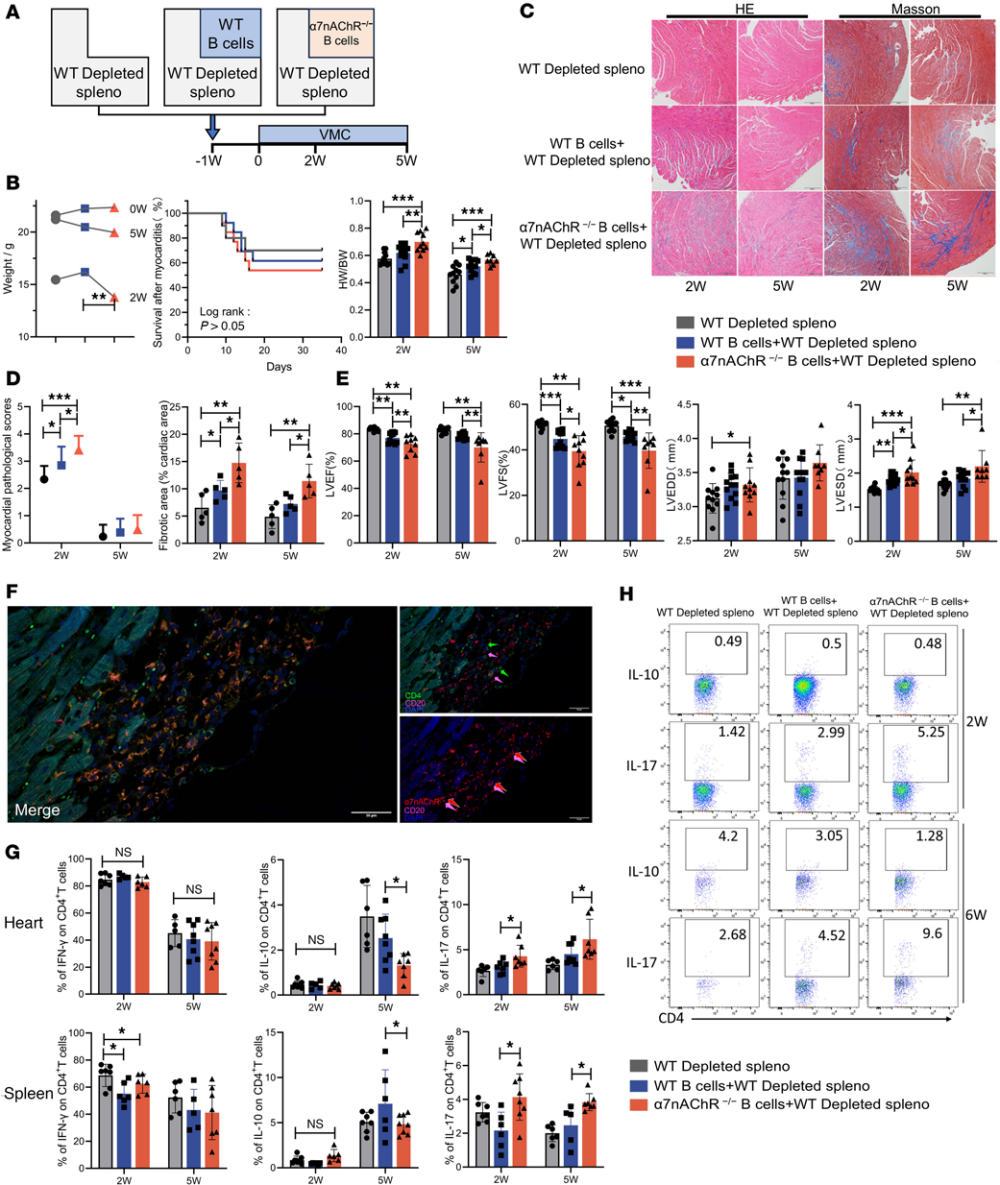
α7nAChR on B cells inhibits their role in exacerbating VMC severity and Th17 differentiation. (**A**) B cell–depleted splenocytes re-supplemented with purified B cells from WT or α7nAChR^−/−^ mice were transferred into SCID mice. (**B**) The body weight loss, survival, and heart weight/body weight (HW/BW) ratio of each group were monitored over time (*n* = 10–11 mice/group). Representative flow histograms (**C**) and quantification (**D**) of myocardial inflammation and fibrotic area after B cell deficiency and B cell–specific α7nAChR knockout were observed at weeks 2 and 5. Original magnification, ×40. (*n* = 5 mice/group). Scale bars: 200 μm. (**E**) The left ventricular ejection fraction (LVEF), left ventricular fractional shortening (LVFS), left ventricular end-diastolic diameter (LVEDD), and left ventricular end-systolic diameter (LVESD) of each group were determined at the endpoint (*n* = 8–12 mice/group). (**F**) Representative images of the merge of CD4 and CD20 channels and CD20 and α7nAChR channels; all channels are shown. Original magnification, ×400. Scale bars: 50 μm. Flow cytometric analysis (**G**) and representative flow cytometric plots (**H**) of IFN-γ–, IL-10–, and IL-17–producing cardiac and splenic T cells of SCID model mice after B cell deficiency and B cell–specific α7nAChR knockout (*n* = 5–8 mice/group). The data are represented as the mean ± SD. *P < 0.05; **P < 0.01; ***P < 0.001 by 1-way ANOVA (**B** [left], **D**, **E**, and **G**) or log-rank (Mantel-Cox) test (**B** [right]).

We subsequently generated a B cell–restricted α7nAChR-deficient mouse model by adoptively transferring splenocytes into SCID mice (Figure 5A). The purity and successful reconstitution of B lymphocytes are shown in Supplemental Figure 2, A and B. As shown in Figure 5, B–E, we found that supplementation with WT B lymphocytes significantly exacerbated myocardial inflammation and fibrosis and worsened cardiac function compared with those in the group that received only B cell–depleted splenocytes, suggesting that B lymphocytes aggravate the severity of acute VMC. Compared with WT B lymphocytes, the adoptive transfer of α7nAChR^–/–^ B lymphocytes resulted in more pronounced infiltration of inflammatory cells and collagen deposition (Figure 5, B–E). In brief, α7nAChR knockout in B cells enhanced their pathogenic effects. We observed that α7nAChR-expressing B cells were more prevalent around T cells (Figure 5F). Compared with the transfer of WT B lymphocytes, the transfer of α7nAChR^–/–^ B lymphocytes into SCID mice increased the proportions of Th17 cells and decreased the proportions of Treg cells in the heart and spleen (Figure 5, G and H, and Supplemental Figure 2C). As a negative regulator, α7nAChR expression by B cells limits its ability to promote Th17 differentiation.

### α7nAChR restrains the proinflammatory phenotypes of B cells.

**Figure 6:**
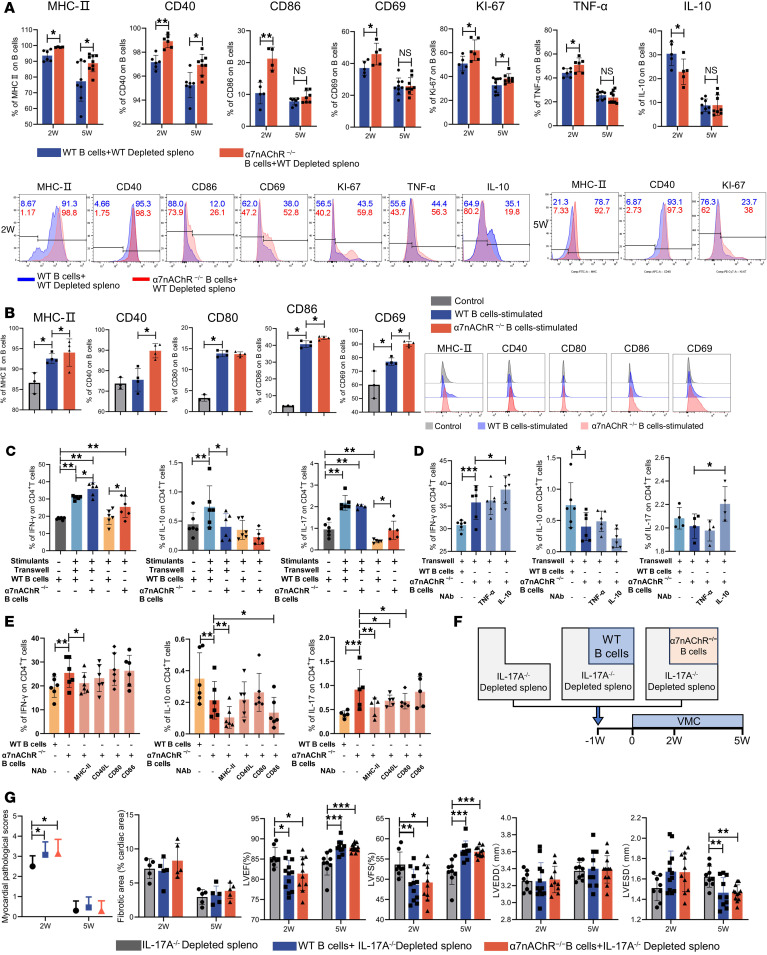
α7nAChR on B cells restrains their proinflammatory phenotypes, blocking VMC progression in a manner dependent on IL-17A. (**A**) Quantification and representative flow histograms of the percentages of MHC-II^+^, CD40^+^, CD86^+^, CD69^+^, Ki-67^+^, TNF-α^+^, and IL-10^+^ cells among gated CD19^+^ B cells from recipient spleens are shown (*n* = 4–9 mice/group). Purified B cells from WT or α7nAChR^−/−^ mice were cultured with or without LPS, anti-CD40, and necrotic cell extract stimulation for 48 hours. (**B**) Quantification and representative flow histograms of the percentages of MHC-II^+^, CD40^+^, CD80^+^, CD86^+^, and CD69^+^ cells among CD19^+^ B cells cultured in vitro (*n* = 3–4). (**C**). Quantitation of in vitro–cultured T cells producing IFN-γ, IL-10, and IL-17 (*n* = 6). Neutralizing antibodies anti–IL-10 and anti–TNF-α (**D**) were added to the Transwell coculture system and anti–MHC-II, anti-CD40, anti-CD80, and anti-CD86 (**E**) were added to the direct coculture system (*n* = 5); the differentiation of Th cells was observed. The results in **B**–**D** were pooled from at least 3 independent experiments. (**F**) Flowchart showing the experimental scheme for transferring IL-17^–/–^ B cell–depleted splenocytes re-supplemented with purified B cells from WT or α7nAChR^−/−^ mice into SCID mice. (**G**) Quantification of myocardial pathological score and the percentage of the fibrotic area in IL-17^−/−^ B cell–depleted splenocytes re-supplemented with WT or α7nAChR^−/−^ B lymphocytes on weeks 2 and 5 are shown (*n* = 6 mice/group). The left ventricular ejection fraction (LVEF), left ventricular fractional shortening (LVFS), left ventricular end-diastolic diameter (LVEDD), and left ventricular end-systolic diameter (LVESD) of each group were determined at the endpoint (*n* = 8–12 mice/group). The data are represented as the mean ± SD. *P < 0.05; **P < 0.01; ***P < 0.001 by 2-tailed, unpaired Student’s *t* test (**A**) or 1-way ANOVA (**B**–**D** and **F**).

Taken together, our results indicate that the cardioprotective effects of α7nAChR are partly mediated by B lymphocytes, which raises the intriguing possibility that α7nAChR can modulate the immune function of B cells during myocardial injury. There was no significant difference in the absolute number of splenic and cardiac B cells between recipient mice that received WT B lymphocytes and α7nAChR^−/−^ B lymphocytes at weeks 2 and 5 of VMC (Supplemental Figure 2D). Flow cytometry revealed that knocking out α7nAChR in B cells increased the expression levels of antigen-presenting molecules such as MHC-II, CD40, CD86, and CD69, as well as Ki-67 and TNF-α and decreased the expression of IL-10 in VMC at week 2 (Figure 6A). MHC-II, CD40, and Ki-67 remained highly expressed at week 5 in B cells after α7nAChR was knocked out (Figure 6A). B cells expressing IL-1β, IL-4, IL-6, IL-17, CCL2, and CD80 were not affected by α7nAChR knockout (Supplemental Figure 2E). Therefore, α7nAChR can inhibit the secretion of proinflammatory cytokines and antigen presentation to modulate the functional activity of B cells in VMC.

To further validate the regulatory role of α7nAChR in B cells, we repeated these experiments in vitro and found that α7nAChR knockout further increased the expression levels of antigen-presenting molecules such as MHC-II, CD86, CD40, and CD69 on B cells (Figure 6B and Supplemental Figure 2F). As shown in Supplemental Figure 3A, low concentrations of an α7nAChR antagonist (methyllycaconitine, MLA) and agonist (PNU-282987) promoted B cell proliferation in vitro; however, at concentrations greater than 10 μM, MLA and PNU-282987 caused a gradual decline in B cell proliferation due to drug toxicity. Based on the results of the proliferation assay, B cells were treated with a low concentration (5 μM) or a high concentration (25 μM) of MLA or PNU-282987. Drug treatment significantly increased the expression levels of CD80, CD22, CD40, CD69, and TNF-α by B cells, whereas the expression levels of CD86, IFN-γ, and the antiinflammatory cytokines IL-4 and IL-10 decreased (Supplemental Figure 3, B and C). Moreover, there was no significant difference in the expression levels of MHC-II, IL-17, and IL-6 compared to those in the control group (Supplemental Figure 3, B and C). We found that long-term exposure to specific α7nAChR agonists had regulatory effects that were consistent with those of the antagonists. Consistent with previous studies, methyltaproine and the long-existing PNU-282987 and anti-α7nAChR antibodies inhibited the secretion of IL-10 by B cells by blocking the α7nAChR signal (28). One study revealed that the α7nAChR is a highly permeable Ca^2+^ channel characterized by rapid activation dynamics (42). Prolonged exposure to agonists leads to rapid desensitization of the current, resulting in receptor downregulation and loss of responsiveness (43, 44). Therefore, α7nAChR can inhibit the secretion of proinflammatory cytokines and reduce antigen presentation to modulate the functional activity of B cells in VMC.

### α7nAChR deletion in B cells promotes Th17 differentiation and impairs Treg differentiation by regulating antigen-presenting molecules.

To further determine whether α7nAChR affects T cell differentiation by inducing cytokine production or through direct contact with B cells, we developed an in vitro T cell–B cell coculture system. Compared with WT B cells, α7nAChR^–/–^ B cells significantly promoted CD4^+^ T cell differentiation toward Th1 and Th17 subsets through direct contact or soluble mediators. In addition, B cells lacking α7nAChR strongly enhanced the differentiation and proliferation of Th1 and Th17 cells and inhibited Treg cell differentiation in vitro (Figure 6C and Supplemental Figure 4A). However, the deletion of α7nAChR expression in B cells promoted the proliferation of Treg cells (Figure 6C and Supplemental Figure 4A). Thus, α7nAChR on B cells restricts proinflammatory changes and inhibits Th17 cell differentiation. In addition, neutralization of MHC-II, CD40, and CD80 counteracted the lack of α7nAChR in B cells to promote the induction of differentiation and the proliferation of Th1 and Th17 cells, whereas neutralization of IL-10 had the opposite effect; neutralization of MHC-II and CD86 enhanced the inhibitory effect of α7nAChR in B cells on the differentiation and proliferation of Treg cells (Figure 6, D and E, and Supplemental Figure 4, B and C). Thus, α7nAChR mainly inhibits the expression of MHC-II, CD40, and CD80 antigen-presenting molecules in B lymphocytes, thereby inhibiting Th1 and Th17 differentiation.

### Il-17A mediates the role of α7nAChR on B cells in preventing the progression of VMC to the chronic phase.

To investigate whether α7nAChR on B cells drives Th17 cell differentiation to affect the progression of VMC to the chronic phase, we injected SCID mice with IL-17A^−/−^ B cell–depleted splenocytes supplemented with WT or α7nAChR^−/−^ B lymphocytes (Figure 6F). The knockdown efficiency of the IL-17A gene knockout in mice was confirmed (Supplemental Figure 4D). As shown in Figure 6G and Supplemental Figure 4, E and F, the pathogenic effects of α7nAChR knockout in B cells were enhanced, which was abrogated by splenocyte knockout of IL-17A. Taken together, these findings identify IL-17 in T cells as a critical downstream effector of the α7nAChR-mediated restriction of B cell pathogenic function and indicate that this negative regulatory axis in the immune microenvironment is essential for preventing the progression of VMC to the chronic phase.

### B cells secrete ACh in an autocrine manner, which acts on α7nAChR to inhibit the NF-κB pathway and activate the JAK2/STAT3 and PI3K/Akt signaling pathways.

**Figure 7:**
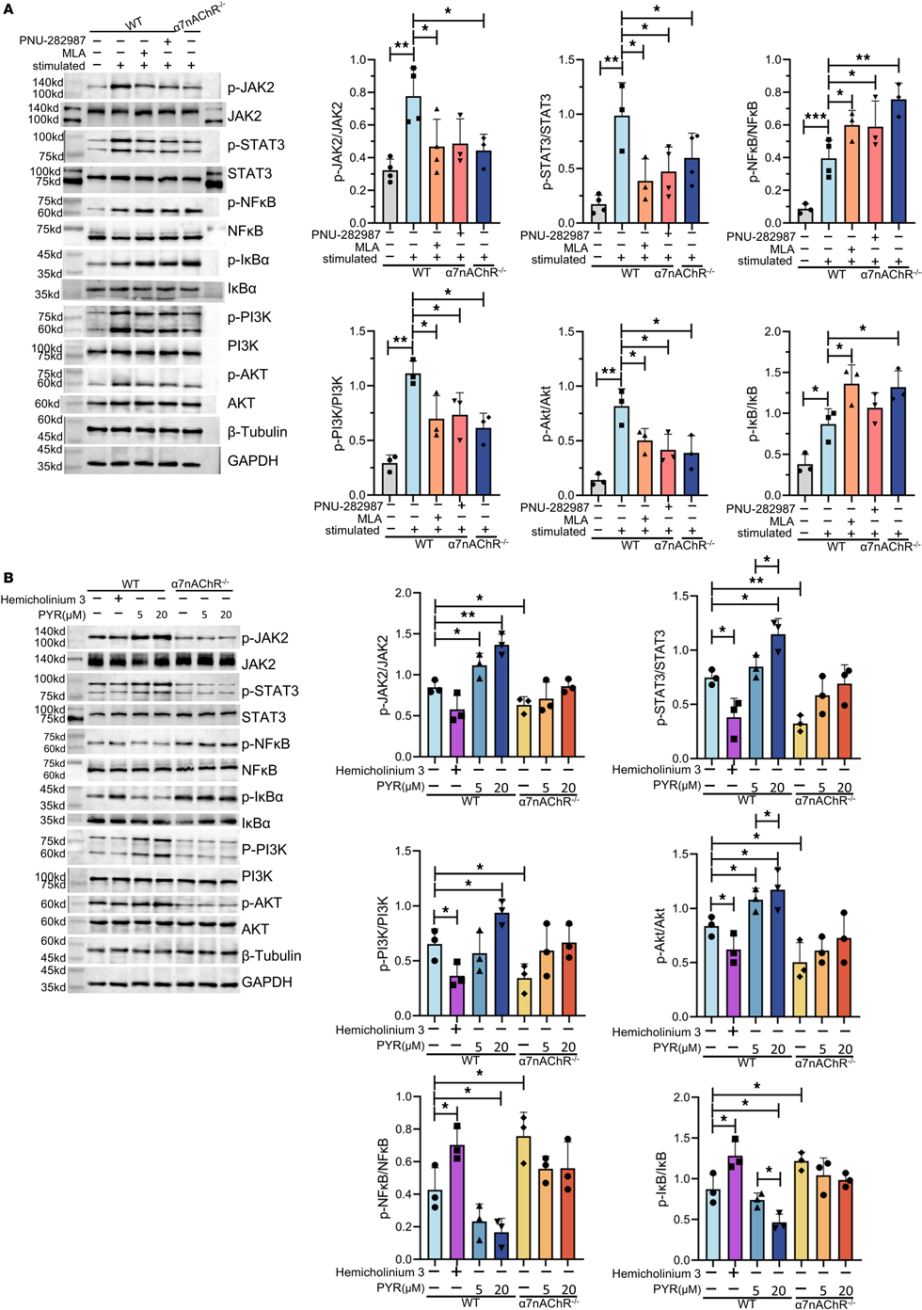
B cells can secrete ACh, which acts on α7nAChR to inhibit the NF-κB pathway while activating the JAK2/STAT3 and PI3K/Akt signaling pathways. Immunoblot analysis was performed using antibodies against the indicated proteins, including p-STAT3, STAT3, p-JAK2, JAK2, p-NF-κB p65, NF-κB p65, p-IκBα, IκBα, p-PI3K, PI3K, p-AKT, and AKT. WT or α7nAChR^−/−^ B cells supplemented with or without stimulants were treated with indicated concentrations of MLA and PNU-282987 (**A**), as well as pyridostigmine bromide (PYR) and hemicholinium-3 (**B**) for 48 hours (*n* = 3). The data were normalized to the total protein and pooled from at least 3 independent experiments (**A** and **B**). The data are represented as the mean ± SD. *P < 0.05; **P < 0.01; ***P < 0.001 by 1-way ANOVA (**A** and **B**).

To determine the downstream signaling pathways involved in the antiinflammatory effects of α7nAChR, we analyzed the changes in related inflammatory signaling pathways, including the NK-κB signaling pathway, the JAK2/STAT3 signaling pathway and the PI3K/Akt signaling pathway. The increased p-JAK2/JAK2, p-STAT3/STAT3, p-PI3K/PI3K, and p-Akt/Akt ratios in stimulated B cells were reduced by PNU-282987, MLA, and α7nAChR knockout; furthermore, these interventions promoted the activation of p-NF-κB and p-IκB in B cells (Figure 7A).

Based on a previous study indicating that B cells can induce ChAT expression via multiple TLR signals and can secrete ACh via α7nAChR to inhibit proliferation (28), we used PYR and hemicholinium-3 to examine the signaling crosstalk of ACh derived from B cells. Hemicholinium-3 is a competitive inhibitor of the high-affinity choline uptake system (HACU) that inhibits the synthesis and release of ACh (45). The addition of hemicholinium-3 significantly reduced the-JAK2/JAK2 and p-STAT3/STAT3 ratios in B cells (Figure 7B). An increase in the concentration of PYR resulted in increases in the ratios of p-JAK2 to JAK2 and p-STAT3 to STAT3, with reductions in the ratios of p-NF-κB to NF-κB and p-IκB to IκB in B cells; however, this change was not observed in α7nAChR^–/–^ B cells (Figure 7B). In summary, B cells can secrete ACh to act on α7nAChR, thereby activating the JAK2/STAT3 pathway and inhibiting the NF-κB pathway.

### Cholinergic activity is impaired during the progression of VMC to the chronic phase.

**Figure 8:**
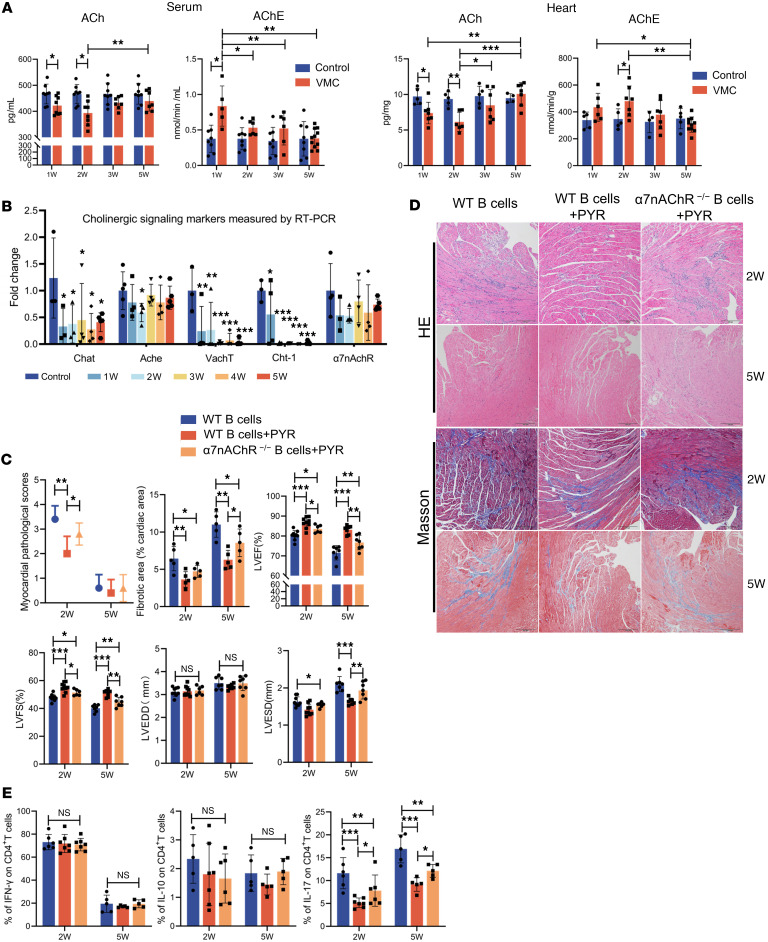
Improvement of cholinergic hypofunction to prevent VMC progression is associated with α7nAChR on B cells. (**A**) The levels of acetylcholine (ACh) and acetylcholinesterase (AChE) in serum and myocardial tissue homogenate supernatants were detected by ELISA and colorimetric assay. (**B**) mRNA expression of *Chat*, *Ache*, *Cht1*, *Vacht*, and *Chrna7* in heart tissue obtained from the VMC and control mice (*n* = 5 mice/group). The data were normalized to *Gapdh*. SCID mice administered B cell–depleted splenocytes and purified B cells transferred from WT or α7nAChR^−/−^ mice were treated with or without PYR. (**C**) Statistical results of pathology scores, fibrosis area, and parameters for cardiac function of SCID mice are shown (*n* = 6 mice/group). (**D**) Representative images showing H&E and Masson’s trichrome staining of hearts from SCID mice. Original magnification, ×40. Scale bars: 200 μm. (**E**) Summary graph showing quantification of the frequency of IFN-γ–, IL-10–, and IL-17–producing cardiac T cells in SCID mice with immune reconstitution (*n* = 5–10 mice/group). The data are represented as the mean ± SD. *P < 0.05; **P < 0.01; ***P < 0.001 by 1-way ANOVA (**A**, **C**, and **E**).

To determine the source of ACh that activates the α7nAChR, we further characterized the dynamic changes in the overall cholinergic system during the progression of VMC. Dysfunction of the autonomic nervous system characterized by sympathetic activation and impaired parasympathetic activity plays prominent roles in the pathogenesis of various cardiovascular diseases (46). To characterize the dynamic changes in the overall cholinergic system during the progression of VMC, we evaluated the levels of ACh and acetylcholinesterase (AChE) in cardiac tissue and serum, as well as the expression levels of ACh-related synthetic and degradative enzymes. The primary source of ACh in serum is peripheral blood immune cells, and we detected a marked decrease in ACh concentrations during the acute phase of VMC, which was accompanied by a marked increase in AChE activity (Figure 8A). The expression levels of *Chat*, *Vacht* (vesicular acetylcholine transporter, VAChT), and *Cht1* (high-affinity choline transporter, ChT-1) in cardiac tissue consistently decreased after CVB3 infection and failed to recover to baseline levels even in the chronic phase, which may contribute to the development of DCM (Figure 8B). Additionally, *AchE* expression was significantly decreased at the 2-week time point, whereas *Chrna7* expression in the heart remained unchanged (Figure 8B). Taken together, these findings suggest the loss of enzymatic activity in the cholinergic system and compromised cholinergic neurotransmission during the pathogenesis of VMC.

### PYR improves cardiac injury and function partly through α7nAChR on B cells.

AChE inhibitors inhibit the breakdown of ACh; therefore, we investigated whether PYR could restore ACh activity in VMC and regulate the immune system to influence disease progression. First, the effect of this intervention was determined by measuring AChE enzymatic activity, which showed a decrease with PYR treatment (Supplemental Figure 5A). Compared with the control conditions, PYR treatment resulted in a striking decrease in inflammatory infiltration and inhibited cardiac dysfunction and myocardial fibrosis (Supplemental Figure 5, B–D). To identify the receptor subtypes involved in the PYR-mediated effects of ACh, we next investigated the therapeutic effects of PYR on α7nAChR^–/–^ VMC mice. Systemic and B cell–restricted α7nAChR knockdown partially abrogated the protective effects of PYR on the progression of VMC to the chronic phase (Figure 8, C and D, and Supplemental Figure 5E). PYR regulated the T cell repertoire by significantly reducing the number of Th17 cells (Figure 8E and Supplemental Figure 5F). Additionally, α7nAChR knockdown in B cells partially inhibited the ability of PYR to decrease Th17 numbers in the heart, indicating that α7nAChR on B cells mediates the therapeutic effects of PYR (Figure 8E and Supplemental Figure 5F). The restoration of cholinergic signaling by PYR after CVB3 infection is a heart-specific phenomenon that cannot be replicated in vitro, highlighting the modulatory role of cholinergic signaling in the complex inflammatory environment in vivo. In summary, ACh exerts therapeutic effects on the progression of VMC to the chronic phase by inhibiting the cardiac remodeling effects of Th17 cells through the α7nAChR on B cells.

## Discussion

The viral clearance–induced autoimmune response leading to the progression of myocardial inflammation to the chronic phase is the major cause of inflammatory DCM. Identifying targeted therapies that limit excessive inflammation and autoimmunity initiation is crucial. Impaired activation and dysfunction of the cholinergic system occur during the development of VMC, and the infiltration of α7nAChR^+^ B cells is significantly reduced in the heart. α7nAChR exhibits distinct changes within the cardiac and splenic B cell subpopulations, potentially affecting the intensity of immune responses. α7nAChR on B cells mainly serves as a negative regulatory target for MHC-II, CD40, and CD80, inhibiting the differentiation of Th17 cells. The restoration of cholinergic activity by PYR significantly improves myocardial injury and remodeling, which involves the regulatory role of α7nAChR on B cells in Th17 cell differentiation. The α7nAChR on B cells mediate the cardiovascular/neuroimmune axis, which suppresses adaptive persistent immune-mediated inflammation to prevent myocardial injury and adverse remodeling. These findings provide a therapeutic target for VMC progression.

α9nAChR expressed on B cells can mediate the neuroimmune pathway between the brain and spleen to increase the secretion of antigen-specific IgG and improve immune function (37). In this study, we found that α7nAChR^+^ B cells also serve as potential neuroimmune mediators and significantly decrease in number as VMC progresses. This is partly attributed to the decreased number of total cardiac B cells (Figure 2). We observed marked reductions in the number of total B cells and the frequency of leukocytes within the hearts and spleens of BALB/c mice susceptible to CVB3 (Figure 2), which contrasts with our previous findings in C57BL/6 mice, in which B cells remained unchanged (12). Following the acute myocarditis phase, nonsusceptible C57BL/6 mice failed to progress to the iDCM phenotype on day 28 and later (47). B cells may contribute to the differences in immune response patterns associated with the genetic predispositions of different mouse strains. B cell loss may also be associated with deficient cell development and maturity in VMC. Our data revealed that immature splenic B cells were persistently decreased and could not recover in VMC (Figure 2). Previous studies have reported that acute MI triggers the release of glucocorticoids from the neuroendocrine system, leading to severe impairment of B cell development at the pro-B cell stage. Reduced B cell progenitor differentiation and enhanced apoptosis result in decreased BM B cells and peripheral B lymphopenia within 3 days after MI (48). Cardiac and splenic B cells subsequently recovered and were highly abundant (8, 48, 49). The contribution of BM B cells to the cardiac and peripheral B cell pools in VMC remains to be elucidated and warrants further study.

The properties of cardiac B cells have recently been substantiated by single-cell RNA-seq (7, 40, 41). The transcriptional profile of cardiac CD21^+^CD23^+^ B cells closely resembles those of splenic transitional 3 (T3) and FO B cells (41). Moreover, the CD21^–^CD23^–^ cluster is similar to those of T1 and newly formed B cells. The temporal changes in intravascular myocardial B cell subsets are not unique to the heart but reflect splenic B cell changes (40, 41). α7nAChR expression increases along with splenic B cell maturation, and the activation of α7nAChR promotes the proliferation and differentiation of immature B cells in the BM but inhibits the proliferation of mature splenic B cells (24–26, 50). Therefore, activated MZ B cells, memory B cells, and plasma cells expand during the acute phase of VMC, while their decreased expression of α7nAChR may result in excessive proliferation and activation of effector B cells, resulting in an insufficient limitation of the progression of the VMC inflammatory response. Conversely, for the B1 cell subgroup, we observed that α7nAChR expression levels were significantly higher in B1 and Breg cells from the heart and spleen than in those from the other subgroups (Figure 3). Research has shown that splenic CD5^+^ and Foxp3^+^ B lymphocytes highly express α7nAChR (28). Blocking or knocking out α7nAChR reduces the expression of Foxp3 and the secretion of IL-10 by B cells in vitro, indicating that α7nAChR is essential for the formation, induction, and function of Breg cells (28). Breg cells regulate the mobilization of inflammatory monocytes after MI and improve myocardial remodeling, and adoptive transfer of Breg cells can alleviate myocardial inflammation through Th17 and Treg balance in VMC (11, 14). α7nAChR activation may promote the restoration of Breg cell proportions and enhance their negative immunoregulatory functions in VMC. High expression of α7nAChR by B1 cells may be equally important for supporting their survival and self-renewal. In addition, in this study, we investigated the dynamic characteristics of splenic B cell subsets in VMC mice and discovered that their distribution was characterized by an overall shift to the terminal stage of differentiation (Figure 3). However, the lack of substantial changes in germinal center B cells may indicate the importance of extrafollicular pathways in B cell development in VMC, which primarily rely on assistance and signaling from Th cells.

Previous work by our group indicated that B cells play a pathogenic role in acute VMC independently of T cells and exhibit significantly enhanced activation, antigen presentation, and secretion (12). Here, we discovered that knocking out or inhibiting α7nAChR further promoted B cell proliferation and activation, as well as the expression of antigen-presenting molecules and proinflammatory cytokines such as MHC-II and TNF-α (Figure 6). CD40, CD86, and CD80 on B cells and CD28 on T cells generate costimulatory signals that stabilize the immunological synapse, thereby constituting a critical pathway for T cell–B cell interactions (51). Studies have reported that cardiac B cells influence the frequency and function of specific leukocyte pools following myocardial injury and that there the numbers of CD4^+^ and CD8^+^ T cells are increased after B cell knockout or depletion (40). The sequencing results demonstrated that cardiac B cells exhibited upregulation of chemokine-chemokine signaling pathways and expressed several cytokines (40). For example, B cell clusters express high levels of CXCR5, which is required for organizing B cells into lymphoid follicles following transverse aortic constriction (7). Additionally, pericardial B cells secrete GM-CSF to promote dendritic cell and T cell expansion, as well as granulopoiesis during MI (9). Therefore, B cells, which are important APCs, can play a crucial role in activating T cells in the inflammatory response in the heart. T cell–mediated adaptive autoimmunity is a critical stage in the progression of VMC to the chronic phase, and we further investigated the effect of B cell α7nAChR on T cells.

Several studies have shown that α7nAChR agonists/inhibitors regulate T cell differentiation. The activation of α7nAChR inhibits Th1 and Th17 differentiation while promoting Treg differentiation, thereby reducing the severity of inflammation in experimental autoimmune encephalomyelitis and VMC (52, 53). However, some researchers have reported that the direct effects of α7nAChR on T cell differentiation differ from its indirect effects. GTS-21 directly activates α7nAChR on T cells to promote differentiation toward Treg cells and effector T cells, whereas GTS-21 activation of α7nAChR on APCs downregulates T cell differentiation by inhibiting antigen-presenting capacity (54, 55). However, previous studies have not investigated the immunomodulatory effects of α7nAChRs on nonimmune cells. α7nAChR is also widely distributed in cardiomyocytes and coronary arteries, and compensatory upregulation of α7nAChR in the myocardium can occur following ischemia-reperfusion injury (56, 57). The α7nAChR on cardiac fibroblasts plays a pathogenic role by promoting right ventricular fibrosis and dysfunction in pulmonary arterial hypertension (58). BM chimeras revealed that α7nAChR expressed by hematopoietic and nonhematopoietic cells could alleviate myocardial inflammation in acute VMC, whereas only α7nAChR on hematopoietic cells played a protective role in preventing fibrosis and improving systolic function during the chronic phase (Figure 4). In addition, it has been demonstrated that α7nAChR on nonhematopoietic cells does not affect Th cell differentiation. Only the deletion of α7nAChR in hematopoietic cells promoted Th17 differentiation and inhibited Treg cell differentiation (Figure 4). The mechanism by which α7nAChR on nonhematopoietic cells such as cardiomyocytes, fibroblasts, or endothelial cells attenuates myocardial inflammation in the acute phase requires further investigation. α7nAChR can also stabilize the mitochondrial membrane, reduce oxidative stress, inhibit autophagy dysfunction, and maintain hemodynamic stability, thereby exerting cardioprotective effects on heart diseases such as ischemia-reperfusion injury and MI (59–61).

Here, we identified α7nAChR on B cells as a negative regulator that limits Th17 differentiation through direct contact or soluble factors; α7nAChR on B cells inhibits Th17 cells, thus preventing VMC progression to the chronic phase (Figure 6). Next, we investigated the cholinergic signaling sources that activate α7nAChR on B cells. The cholinergic system includes neuronal cells (via the vagus nerve) and nonneuronal cells (such as immune cells and cardiomyocytes) (62). On the one hand, immune cells such as CD4^+^ T cells act as intermediaries in the cholinergic antiinflammatory pathway by secreting ACh to inhibit proinflammatory macrophage functions (63). Researchers have shown that B cells express higher levels of ChAT than CD4^+^ T cells do; ChAT^+^ B cells have reduced expression of endothelial adhesion molecules, thus inhibiting the recruitment of local neutrophils (63). In addition, in vitro experiments have shown that PYR can reduce the level of endogenous ACh produced by B cells in a dose-dependent manner, thus inhibiting their proliferation (28). Thus, we further showed that ACh secreted by B cells acts on α7nAChR to activate the antiinflammatory JAK2/STAT3 and PI3K/Akt signaling pathways and inhibit the proinflammatory NF-κB pathway (Figure 7). These findings suggest that B cells can use autocrine ACh to negatively regulate their own functions, thus limiting excessive inflammatory damage. On the other hand, we observed substantial decreases in the expression levels of ChAT, VAChT, and ChT-1 in the heart, whereas AChE expression was increased (Figure 8). As a result, ACh synthesis was reduced, and ACh degradation was increased, leading to a marked decrease in ACh levels in the myocardium (Figure 8). The serum concentration of ACh is significantly decreased in VMC, as indicated by research demonstrating that approximately 60% of ACh in human peripheral blood is derived from leukocytes (64). However, in myocardial tissue, the proportion of immune cells expressing ChAT was relatively low, and most ChAT immunofluorescence was localized in cardiac muscle fibers (Figure 1). Previous studies have reported that cardiomyocytes can secrete increased levels of ACh, which may compensate for the sparse distribution of the vagus nerve in ventricular tissue (65). In addition, studies have demonstrated that right vagotomy exacerbates myocardial inflammation in VMC (66); however, transection of the vagus nerve leads to an increase in sympathetic nerve trafficking toward the heart, thereby activating rapid immune responses (67). This phenomenon introduces confounding factors related to the interaction between parasympathetic and cardiac sympathetic nerve signals, which may complicate these studies (68). Future work investigating ACh derived from different immune cell sources will still require the use of mice with targeted ChAT-GFP knockin for refinement.

Our findings revealed a decrease in the activity and impaired function of the cardiac and systemic cholinergic systems at the onset and during the development of VMC. ACh ultimately requires specific nAChRs on certain cells to act as intermediaries, which is not the initial disease factor. The α7nAChR on B cells can be rapidly activated for downstream signaling under both physiological and pathological conditions, which is the focus of this study. Recent studies have revealed an important homeostatic role for PYR in the heart, as it regulates the immune microenvironment. In mouse models of MI and pulmonary arterial hypertension–induced right ventricular overload, PYR increased the numbers of M2 macrophages and Foxp3^+^ Treg cells and reduced CCL2/7 chemotactic signaling, leading to decreased infiltration of MHC-II^hi^CCR2^+^ macrophages (19–21). In the present study, we found that PYR significantly improved myocardial inflammation, fibrosis, and cardiac dysfunction in VMC mice and that these effects were partially mediated by α7nAChR expressed on B cells (Figure 8). Multiple-staining analysis revealed that ChAT and α7nAChR are adjacent to immune cells (Figure 1), indicating the presence of rapid cardiac/neural/immune communication that acts as a local “early warning” system during myocardial injury. This study raises important questions about the immunomodulatory role of the cholinergic system in the heart and the development of effective therapeutic strategies targeting the cardiac/neural/immune axis.

On the one hand, the brain regulates systemic inflammatory responses by activating adaptive immune responses in peripheral lymphoid tissues via the autonomic nervous system. On the other hand, neuroimmune communication in the local microenvironment regulates the maintenance of local homeostasis in the cardiovascular system. For example, neuroimmune-cardiovascular interfaces are composed of axon terminals, smooth muscle cells, and leukocytes in the adventitia of blood vessels, which are innervated by the CNS through the sympathetic nervous system. Surgical removal of the celiac ganglia can disrupt arterial tertiary lymphoid organs and alleviate atherosclerosis (69). This local neuroimmunoregulatory axis has also been identified in the intestine and lungs. LYVE1^lo^MHC-II^hi^ interstitial macrophages in the lung are located around sympathetic nerves, and intestinal neurons stimulate β2-adrenergic receptors on muscularis macrophages following bacterial infection, ensuring rapid initiation of tissue-protective responses to distant stressors (70, 71). These studies provide a theoretical basis for clinical applications, such as invasive or noninvasive vagus nerve stimulation and cholinergic drug therapy. However, the desensitization of AChRs may limit the efficacy of α7nAChR agonists in treating inflammatory diseases. Clinical trials have shown that GTS-21 intervention in patients with endotoxemia does not lead to substantial differences in the serum levels of the proinflammatory cytokines TNF-α and IL-6 compared to those in controls (72). Studies have reported that allosteric modulators can enhance the efficacy of receptor agonists by binding ligands at both orthosteric and allosteric sites. Positive allosteric modulators can restore the activity of desensitized channels and even activate α7nAChR in the absence of agonists (73).

### Study limitations

The establishment of a Cre-lox mouse on the BALB/c background is cost and time intensive, making it impractical to establish a model that avoids the confounding effect of immune reconstitution. And further exploration through optogenetics is necessary to trace the anatomical circuits between α7nAChR on B cells and cholinergic and parasympathetic nerves.

### Conclusion

Our results provide essential insights into the ontology, development, and distribution of cardiac B cells. Cholinergic signaling partially regulates Th17 differentiation via α7nAChR on B cells, which helps to impair the progression from VMC to the chronic stage and plays a beneficial immunomodulatory role in the heart. Our study will open avenues for research and the treatment of inflammatory heart disease. Furthermore, our findings prompt a reevaluation of our understanding of the role that neuroimmune interactions play in the heart under inflammatory conditions.

## Methods

Further information can be found in Supplemental Methods.

### Sex as a biological variable.

All experiments were conducted using male mice. It has been demonstrated that male mice are more prone to developing VMC, as they experience more severe myocardial inflammation and are more likely to progress to the chronic phase.

### Animals.

α7nAChR^–/–^ mice were generated on a BALB/c background using the CRISPR/Cas9 system at Vital River Laboratory Animal Technology Company. Two single-guide RNAs (sgRNAs) were designed to target exons 5 and 10 of the mouse *Chrna7* gene (ENSMUSG00000030525): sgRNA1, CAATTCATATGAAACGGACGAGG; and sgRNA2, TTGTCATGGGCTCACCCGATAGG. The sgRNA vectors and Cas9 vector were microinjected into fertilized eggs. These manipulated zygotes were subsequently transferred into surrogate female mice. After birth, tail tissue was harvested from F1 generation mice for PCR genotyping. The primers used were F, TTATCCTCTCTGCTCTTGACTGTGC; R1, CCCTGGTGTAAAGTTGACATTGGTA; and R2, TACCTGGAGGGAGATACTGGCAA. In addition, the efficacy of α7nAChR knockdown was assessed by Western blotting. *Il17a*^–/–^ mice were generated using CRISPR/Cas9 on a BALB/c background (IL17A^–/–^ mice) by GemPharmatech Company. Four sgRNAs were designed to target exons 1 and 2 of the mouse *Il17a* gene (ENSMUSG00000030525): sgRNA1, AGTGCCGACAAACAACGGGT; sgRNA2, CCACTGACTCATGAGCTAAC; sgRNA1, CTATAGACAGGTGGCTCCAC; and sgRNA2, TGGTAGAGGAAGCATCCTGC. The knockout lines were analyzed by PCR. The primers used for PCR1 were F, ATGCTCTGCACTCGTATTCTCATG; and R, GGCTGTTCATCTGATTTGGTGAC. The primers used for PCR2 were F, AAACACTGAGGCCAAGGACTTCCT; and R, GCTCTCATGCTAGAATCATGGTTATCA. Because IL-17A is a secreted protein, we validated the knockout efficiency of the IL-17A protein using flow cytometry and stimulated T cells in vitro with PMA, ionomycin, and Brefeldin A. All WT BALB/c mice and BALB/c SCID (SCID/SCID) mice were purchased from Vital River Laboratories. All male mice used in this study were 5 weeks old and were fed under specific pathogen–free conditions, whereas the SCID mice were maintained under sterile conditions at the Guangxi Medical University Laboratory Animal Center. Each group of 20 male mice was assigned randomly. At the end of the exposure period, the mice were euthanized by CO_2_ narcosis followed by cervical dislocation.

### VMC induction.

Heart-passaged CVB3 was produced as described in a previous publication (47). The viral titer was determined using the 50% tissue culture infectious dose (TCID_50_) assay, and the viral stocks were aliquoted and stored at −80°C until further use. We established a VMC mouse model by intraperitoneal immunization with 0.1 mL of PBS containing 1 × 10^4^ TCID_50_ heart-passaged CVB3 on day 0, whereas mice that were intraperitoneally injected with an equal volume of PBS served as controls (47). The mice were observed daily to record mortality and monitor changes in body weight and behavioral status, which are important manifestations of the infection model. The mice were sacrificed at the corresponding time points after injection. The ratio of heart weight to body weight can indicate the severity of conditions such as acute myocardial edema and chronic myocardial hypertrophy (74). Transthoracic echocardiography was performed to evaluate cardiac function, after which the spleen, peripheral blood, and heart were collected aseptically for further pathological analysis after perfusion.

### Statistics.

Statistical analyses were performed in SPSS version 26 (SPSS Software, IBM). The data are presented as mean ± SD as indicated in the legends. Two-tailed, unpaired Student’s *t* tests were performed to assess for significant differences between 2 groups. For the analysis of 3 or more groups, 1-way analysis of variance (ANOVA) was employed, followed by LSD post hoc tests for equal variances and Tamhane’s T2 test for unequal variances. Survival rates were compared between groups using the log-rank (Mantel-Cox) test. A *P* value of less than 0.05 was considered significant.

### Study approval.

All research protocols were reviewed and approved by the Experimental Animal Ethics Committee of the First Affiliated Hospital of Guangxi Medical University (approval number 2023-E315-01) in Nanning, China, and all procedures conformed to the NIH *Guide for the Care and Use of Laboratory Animals* (National Academies Press, 2011).

### Data availability.

The authors confirm that the data supporting the findings of this study are available within the article and the Supporting Data Values file in the supplemental material.

## Author contributions

JL and WW conceived and designed the experiments. JL, KC, ZC, LC, YH, and YL carried out the experiments. JL analyzed the data. KC wrote the manuscript. All the authors reviewed and provided feedback on the manuscript.

## Supplementary Material

Supplemental data

Unedited blot and gel images

Supporting data values

## Version 1 05/08/2025

Electronic publication
