# Supplementary material for: α7nAChR on B cells directs T cell differentiation to prevent viral myocarditis
Source: JCI Insight. 2025 May 8;10(9):e189323. doi: 10.1172/jci.insight.189323 (PMC12128988; doi:10.1172/jci.insight.189323)
Supplement: Supplemental data [file jciinsight-10-189323-s148.pdf]

Supplemental Materials

Supplemental Methods

Drug intervention

From CVB3 infection until the observation endpoint at week 2 or week 5, the mice in the PYR group were given the anticholinesterase agent PYR (Cat. HY-B0207A; MCE, USA) dissolved in water at a dose of 30 mg/kg. The drinking volume of the solution was monitored daily, and the concentration of PYR was adjusted to maintain a daily average intake dose of 30 mg/kg/day (approximately 0.1 mg/mL)(1). The blank group was provided the same volume of blank solvent.

Generation of BM chimeric mice

WT or α7nAChR$^{-/-}$ recipient mice were irradiated with a total of 600 rads of RS2000 (Radsource, USA)(2). The femurs and tibias of WT or α7nAChR$^{-/-}$ donor mice were collected, flushed with sterile PBS, and filtered through a 70 µm cell strainer as previously described. Within 4 hours of irradiation, the recipient mice were intravenously reconstituted with a total donor BM suspension containing 1×10^7 cells. Recipient mice were maintained with antibiotic water containing 1.2 mg/mL gentamycin (Aladdin, China) and X-ray-sterilized food from 3 days before irradiation until 2 weeks after irradiation. The animals were then allowed to reconstitute for at least 4 weeks prior to the induction of VMC. Tail tissue, BM cells, PBMCs, splenocytes, and myocardial tissue after perfusion were obtained at 2 and 5 weeks after VMC induction. PCR analysis of isolated genomic DNA was used to confirm the reconstitution efficiency of the resulting chimeric mice after BM transplantation.

Histopathology

The hearts were excised and fixed in 4% paraformaldehyde for 24 hours, followed by embedding in paraffin. The wax blocks of heart tissue were sectioned transversely into 5-µm-thick slices and stained with hematoxylin and eosin (HE) to evaluate the severity of myocardial inflammation according to a semiquantitative pathological score: grade 0, no cardiac inflammation; grade 1, < 25%; grade 2, 25–50%; grade 3, 50–75%; and grade 4, > 75%. Cardiac fibrosis was assessed by Masson's trichrome staining. Two independent investigators analyzed five fields per section at a magnification of 40× using an Olympus microscope (Olympus, Japan).

Echocardiography

On days 7, 14, 21, and 35 after infection, the mice in each group were subjected to echocardiography using a MylabSat system (Esaote, Italy) with a 22 MHz ultrasound probe. Ketamine, with minimal impact on left ventricular function and heart rate, is the optimal anesthetic agent for conducting echocardiography; however, owing to its limited accessibility for purchase, we opted for alternative avertin (290 mg/kg; Aibei, China), which closely follows ketamine and rigorously control the dosage to maintain the heart rate within the range of 450 ± 50 beats per minute (bpm)(3). The heart rate was maintained at 450 ± 50 beats per minute (bpm) by administering 1.25% avertin

(0.1 mL/10 g; Aibei, China) for light anesthesia while allowing spontaneous respiration. Standard two-dimensional short- and long-axis views were obtained from the parasternal position to evaluate the left ventricular end-systolic diameter (LVESD) and the left ventricular end-diastolic diameter (LVEDD). The left ventricular ejection fraction (LVEF) and left ventricular fractional shortening (LVFS) were subsequently calculated using Teichholz's formula. Three independent measurements were taken per animal by investigators who were blinded to the experimental groups.

Cell purification and adoptive transfer

The spleens of α7nAChR$^{-/-}$, IL17$^{-/-}$, and WT mice were gently crushed, red blood cells were lysed with erythrocyte lysis buffer (Solarbio, China), and the samples were washed with PBS. The splenocyte suspensions were filtered through a 70-μm sieve (BD Biosciences, USA). A mouse CD19 microbead B-cell isolation kit (Cat. 130-052-201;Miltenyi Biotec, Germany) was used to purify CD19^{+} B cells and B-cell–depleted splenocytes from WT mice according to the manufacturer's instructions. The purity of the isolated B cells exceeded 97%. SCID mice received 1.2×10^{7} WT or IL-17A$^{-/-}$ B-cell–depleted splenocytes supplemented with 8×10^{6} WT B lymphocytes or with 8×10^{6} α7nAChR$^{-/-}$ B lymphocytes through the lateral tail vein 7 days before the induction of VMC with CVB3. T- and B-cell reconstruction was confirmed by flow cytometric analysis of the spleen and heart. The animals were then allowed to reconstitute for 1 week before the induction of VMC.

Cell culture

The purified B cells were used for subsequent in vitro experiments. We used LPS (10 μg/mL, Sigma–Aldrich, USA), anti-CD40 (2.5 μg/mL, Cat. BE0016-2; Bio-X-Cell, USA), and necrotic cell extracts to stimulate the cell cultures. To isolate necrotic cell extracts to simulate antigen processing and presentation of cardiac antigens by B cells after cardiac injury, fresh 6-week-old BALB/c mouse hearts were mechanically fragmented, followed by three freeze–thaw cycles. The heart suspensions were then centrifuged, and the supernatant was extracted to measure the protein concentration(4). Unstimulated B cells and B cells stimulated with varying concentrations of PNU-282987 (Cat. HY-12560A; MCE, USA), methyllycaconitine (MLA; Cat. HY-N2332A; MCE, USA), PYR (Cat. HY-B0207A; MCE, USA), or hemicholinium 3 (Cat. HY-B2152; MCE, USA) were cultured for 48 hours. B-cell proliferation was determined by CCK8 assays (Dojindo, Japan) according to the manufacturer's instructions. For in vitro studies, PNU-282987 and MLA were used at concentrations of 5 and 25 μM, respectively, whereas PYR was used at concentrations of 5 and 20 μM, and hemicholinium 3 was used at a concentration of 10 μM.

Coculture of B and T cells

Naïve T cells were isolated from WT spleens using a naïve T-cell isolation kit (Cat. 130-104-453;Miltenyi Biotec, Germany) according to the manufacturer's protocol. The purified B cells were stimulated in complete RPMI 1640 medium for 48 hours as

previously described and were subsequently washed with buffer. Naïve T cells were incubated alone or with activated B cells for 2.5 days with or without a Transwell chamber (Corning, USA). In vitro coculture assays were performed on cell culture inserts with porous polycarbonate filters (0.4 μm pore size) in 24-well plates. B cells (1 $\times 10^6$) in 200 μL of 10% RPMI 1640 medium were added to the upper compartment, and 2×10^6 naïve T cells in 600 μL of the same medium were added to the lower compartment. Flow cytometry was used to eliminate B-cell contamination and for further analysis.

RT–PCR

Total RNA was isolated from cardiac tissue using TRIzol® Reagent (Invitrogen, USA) and subsequently transcribed into complementary DNA according to the manufacturer's instructions (Cat. RR047A; Takara, Tokyo). Real-time PCR amplification was carried out on a 7500 Real-Time PCR System (Applied Biosystems, USA) in 20 μL reaction volumes containing SYBR Premix Ex Taq™ II (Cat. RR820A; Takara, Tokyo). GAPDH was used to normalize gene expression. The following sense and antisense primers were used: mouse choline acetyltransferase (*Chat*), 5'- CCATTGTGAAGCGGTTTGGG -3' (sense) and 5'- GCCAGGCGGTTGTTTAGATACA' -3' (antisense); mouse acetylcholinesterase (*Ache*), 5'- CTCCCTGGTATCCCCTGCATA -3' (sense) and 5'- GGATGCCCAGAAAAGCTGAGA' -3' (antisense); mouse *Chrna7*, 5'- CACATTCCACACCAACGTCTT -3' (sense) and 5'- AAAAGGGAACCAGCGTACATC' -3' (antisense); mouse vesicular acetylcholine transporter (*VachT*), 5'- GCCCATTGTTCCCGACTATATC -3' (sense) and 5'- AATAGCACGCCTATCTTCACATC' -3' (antisense); and mouse choline transporter 1 (*Cht1*), 5'- ATGTCTTTCCACGTAGAAGGACT -3' (sense) and 5'- TTGCCGCTGTTTTTGGTTTTC' -3' (antisense). CVB3, 5'- CCCTGAATGCGGCTAATCC -3' (sense) and 5'- ATTGTCACCATAAGCAGCCA -3' (antisense).

WB

To extract protein from B cells, we used lysis buffer containing RIPA buffer (Biyuntian, China), protease and phosphatase inhibitors (MeilunBio, China), and 0.1 M PMSF (Biyuntian, China). A BCA Protein Assay Kit (Thermo Fisher Scientific, USA) was used to determine the protein concentration of each sample. SDS–PAGE was performed for protein resolution, followed by transfer to a polyvinylidene difluoride membrane (Cat. IPVH00010; Millipore, USA) and blocking with QuickBlock™ blocking solution (Biyuntian, China). The membranes were incubated overnight with primary antibodies against phospho-STAT3 (Cat. 9145S; Cell Signaling, USA), STAT3 (Cat. 12640S; Cell Signaling, USA), phospho-JAK2 (Cat. 3776S; Cell Signaling, USA), JAK2 (Cat. 3230S; Cell Signaling, USA), phospho-NF-κB p65 (Cat. 3033S; Cell Signaling, USA), NF-κB p65 (Cat. 4764S; Cell Signaling, USA), phospho-IκBα (Cat. 2859S; Cell Signaling, USA), IκBα (Cat. 4812S; Cell Signaling, USA), PI3 kinase p85 (Cat. 4292S; Cell

Signaling, USA), phospho-PI3 kinase p85 (Tyr458)/p55 (Tyr199) (Cat. 17366S; Cell Signaling, USA), phospho-Akt (Ser473) (D9E) (Cat. 4060S; Cell Signaling, USA), Akt (Cat. 9272S; Cell Signaling, USA), α7nAChR (Invitrogen, Cat. PA5-115651), and GAPDH (Cell Signaling, Cat. 5174) at dilutions of 1:1000. HRP-conjugated anti-rabbit secondary antibodies (Cat. SA00001-2; Proteintech, USA) were used at a dilution of 1:10,000. Finally, the membranes were incubated with enhanced chemiluminescence (ECL) solution (Biyuntian, China), and images were acquired using a Bio-Rad ChemiDoc imaging system (Bio-Rad, USA).

Flow cytometry

After removal of pericardial adipose tissue, mouse hearts were mechanically dissociated and incubated in digestion solution supplemented with collagenase II (300 U/mL, Cat. 17101015; Invitrogen, USA) and hyaluronidase (60 U/mL, Cat.abs47014926; Absin, China) in a shaker incubator at 37°C for 40 minutes. Cardiac B-cell analysis was specifically performed using purified collagenase (300 U/mL, Cat. LS005277; Worthington, USA). The resulting digested solution was neutralized with PBS containing 2 μM EDTA and 1% FBS, followed by filtration through a 40-μM Celltrics filter to obtain the cells. Red blood cells were lysed in ACK buffer (Cat. 555899; BD Biosciences, USA), and the cells were stained with trypan blue to assess cell viability. To prevent nonspecific staining, single-cell suspensions of heart cells were incubated with purified anti-mouse CD16/CD32 (Fc block; 2.4G2; BD Biosciences, USA) at 4°C for 15 minutes. The cells were then stained with Fixable Viability Dye (Cat. 65-0866-18; eBiosciences, USA) to identify dead cells. In addition, B and T cells were stimulated with PMA (50 ng/mL; Cat. HY-18739; MCE, USA) and ionomycin (1 μg/mL; Cat. HY-13434; MCE, USA) for 6 hours, followed by the addition of BFA (10 μg/mL; Cat. HY-16592; MCE, USA) for the last 4 hours to induce the secretion of cytokines. For surface staining, the cells were then stained with different antibodies, including APC-conjugated anti-mouse IFN-γ (XMG1.2; Invitrogen, USA), FITC-conjugated anti-mouse IFN-γ (16-10A1; BD Biosciences, USA), PE-conjugated anti-mouse IL-10 (JES5-16E3; BD Biosciences, USA), PE-conjugated anti-mouse IL-1beta (NJTEN3; Invitrogen, USA), APC-conjugated anti-mouse IL-4 (11B11; Invitrogen, USA), FITC-conjugated anti-mouse IL-6 (MP5-20F3; Invitrogen, USA), APC-conjugated anti-mouse TNF-α (MP6-XT22; Invitrogen, USA), BV421-conjugated anti-mouse IL-17 (TC11-18H10.1;BioLegend, USA), PE-Cy7-conjugated anti-mouse IgM (RMM-1;BioLegend, USA), APC-Cy7-conjugated anti-mouse IgM (RMM-1;BioLegend, USA), PE-conjugated anti-mouse IL-10 (JES5-16E3;BD Biosciences, USA), PE-Cy7-conjugated anti-mouse IL-1beta (NJTEN3; Invitrogen, USA), APC-conjugated anti-mouse IL-4 (11B11; Invitrogen, USA), FITC-conjugated anti-mouse IL-6 (MP5-20F3; Invitrogen, USA), PE-conjugated anti-mouse MHC-II (M5/114.15.2; Invitrogen, USA), APC-conjugated anti-mouse TNF-α (MP6-XT22; Invitrogen, USA), APC-Cy7-conjugated anti-mouse CD45.2 (104; BioLegend, USA), BV421-conjugated anti-mouse IL-17 (TC11-18H10.1; BioLegend, USA), PE-conjugated anti-mouse CD80 (16-10A1; BD Biosciences, USA), PE-Cy7-conjugated anti-mouse CD86 (GL1; Invitrogen, USA), and FITC-conjugated anti-mouse MHC-II

(M5/114.15.2; Invitrogen, USA). For intracellular staining, cells were initially surface-stained as previously described, followed by fixation and permeabilization using an Intracellular Fixation & Permeabilization Buffer Set (eBiosciences, USA). APC-conjugated anti-mouse IFN-γ (XMG1.2; Invitrogen, USA), FITC-conjugated anti-mouse IFN-γ (16-10A1; BD Biosciences, USA), PE-conjugated anti-mouse IL-10 (JES5-16E3; BD Biosciences, USA), PE-Cy7-conjugated anti-mouse IL-1beta (NJTEN3; Invitrogen, USA), APC-conjugated anti-mouse IL-4 (11B11; Invitrogen, USA), FITC-conjugated anti-mouse IL-6 (MP5-20F3; Invitrogen, USA), APC-conjugated anti-mouse TNF-α (MP6-XT22; Invitrogen, USA), and BV421-conjugated anti-mouse IL-17 (TC11-18H10.1;BioLegend, USA) were incubated with the cells at 4°C for 30 min. The cells were permeabilized with a FoxP3/Transcription Factor Staining Buffer Set (eBiosciences, USA) according to the manufacturer's instructions, and PE-Cy7-conjugated anti-mouse Ki-67 (SolA15; Invitrogen, USA) was used to determine cell proliferation. The cells were subsequently stained with antibodies specific to intracellular antigens. Samples from the spleen and heart were collected on a FACS Canto Flow Cytometer (BD Biosciences, USA). Isotype-matched controls with continuous expression of markers were utilized in flow cytometry assays, and 123count eBeads™ Counting Beads (Cat.01-1234-42; eBiosciences, USA) were used to count each cell type. The absolute number of cells in cardiac tissues was calculated by multiplying the percentage of viable target cells by the total number of cells in the sample divided by the weight in milligrams. FlowJo V10 software (BD Biosciences, USA) was used to analyze the data.

ACh and AChE detection

The levels of secreted ACh and AChE in the serum samples, the supernatants of the heart tissue homogenates, and the supernatants of the B-cell cultures were measured using an ACh ELISA Kit (Cat. E-EL-0081c; Elabscience, China) and an AChE Assay Kit (Cat. AK339; Bioss, China), respectively. The absorbance was measured with a Varioskan™ LUX multimode microplate reader (Thermo Scientific, USA).

Luminex assay

The cell-free supernatants of the B-cell cultures were thawed, and cytokines were measured using a Luminex Mouse Premixed Multi-Analyte Kit (Cat. LXSAMSM-08; R&D Systems, USA) according to the manufacturer's instructions. We measured and analyzed seven cytokines: CCL2, IFN-γ, TNF-α, IL-1β, IL-4, IL-6, IL-17, and IL-10. The Luminex plates were read using a Luminex 200 System (Luminex Corporation, Austin, TX, USA) according to the manufacturer's instructions. MILLIPLEX Analyst (Millipore Sigma, Billerica, MA) was used to analyze the data.

Multiplex immunohistochemistry

Multiple immunohistochemistry (IHC) was performed in sequential mode as follows. Briefly, paraffin sections of formalin-fixed heart tissue obtained from VMC mice were deparaffinized and subjected to microwave treatment in citrate buffer for antigen retrieval. The sections were then blocked and stained with commercially available anti-

CD20 (1:200, ab64088, Abcam, USA), anti-CD4 (1:100, ab183685, Abcam, USA), anti-CD68 (1:100, ab283654, Abcam, USA), anti-α7nAChR (1:100, PA5-115651, Thermo Fisher, USA), and anti-ChAT (1:1000, ab178850, Abcam, USA) antibodies at room temperature for one hour. Subsequently, the samples were incubated with goat anti-rabbit IgG H&L (HRP) (1:500, ab6721, Abcam, USA) at room temperature for 30 minutes. Visualization of tyramide signal amplification (TSA) was achieved using TY fluorophores. After each staining cycle, elution treatment was carried out to remove the antibody-TSA complex. Multiplex TSA was optimized by performing a triplex (CD4, TY488; CD68, TY555; α7nAChR, TY594; CHRNA7, TY640; CD20 TY745), followed by DAPI counterstaining. The fluorescence was measured using a multispectral imaging system (KF-PRO-005, Konfoong Biotech International) and quantified in HALO software (Indica Labs, USA).

Statistics

Statistical analyses were performed in SPSS version 26 (SPSS Software, IBM, USA). The data are presented as the means ± SDs as indicated in the legend. Two-tailed unpaired Student's t tests were performed to assess for significant differences between the two groups, with a P value less than 0.05 considered significant. For the analysis of three or more groups, one-way analysis of variance (ANOVA) was employed. Survival rates were compared between groups using the Log-rank (Mantel-Cox) test.

References

1. de Cuba MB, Machado MP, Farnesi TS, Alves AC, Martins LA, de Oliveira LF, et al. Effects of cholinergic stimulation with pyridostigmine bromide on chronic chagasic cardiomyopathic mice. *Mediators Inflamm.* 2014;2014:475946.
2. Seo N, Shirakura Y, Tahara Y, Momose F, Harada N, Ikeda H, et al. Activated CD8(+) T cell extracellular vesicles prevent tumour progression by targeting of lesional mesenchymal cells. *Nature communications.* 2018;9(1):435.
3. Pachon RE, Scharf BA, Vatner DE, and Vatner SF. Best anesthetics for assessing left ventricular systolic function by echocardiography in mice. *Am J Physiol Heart Circ Physiol.* 2015;308(12):H1525-9.
4. Van der Borght K, Scott CL, Martens L, Sichien D, Van Isterdael G, Nindl V, et al. Myocarditis Elicits Dendritic Cell and Monocyte Infiltration in the Heart and Self-Antigen Presentation by Conventional Type 2 Dendritic Cells. *Front Immunol.* 2018;9:2714.

Supplemental Table 1 Phenotypic definitions of the splenic and cardiac B-cell subsets

Organ	Cell subset	Phenotypic definition
Spleen	B1a cells	$CD19^{+}CD43^{+}CD23^{-}CD5^{+}$
Spleen	B1b cells	$CD19^{+}CD43^{+}CD23^{-}CD5^{-}$
Spleen	Breg cells	$CD19^{+}CD1d^{+}CD5^{+}$
Spleen	Immature B cells	$CD19^{+}CD93^{+}$
Spleen	Marginal zone B cells	$CD19^{+}CD93^{-}$ $CD23^{-}IgM^{+}$
Spleen	Follicular B cells	$CD19^{+}CD93^{-}$ $CD23^{+}$
Spleen	Memory B cells	$CD19^{+}CD93^{-}$ IgM^{-}
Spleen	Plasmablasts	$CD19^{lo}CD138^{hi}CD22^{+}$
Spleen	Plasma cells	$CD19^{lo}CD138^{hi}CD22^{-}$
Spleen	Germinal center B cells	$CD19^{+}$ $CD38^{+}$ $GL7^{-}$
Heart	B1 cells	$CD19^{+}$ $CD11b^{+}$
Heart	B1a cells	$CD19^{+}CD11b^{+}$ $CD5^{+}$
Heart	B1b cells	$CD19^{+}CD11b^{+}$ $CD5^{-}$
Heart	$CD23^{+}CD21^{+}$cells	$CD19^{+}CD11b^{-}CD23^{+}CD21^{+}$
Heart	CD23-CD21-cells	$CD19^{+}CD11b^{-}CD23^{-}CD21^{-}$

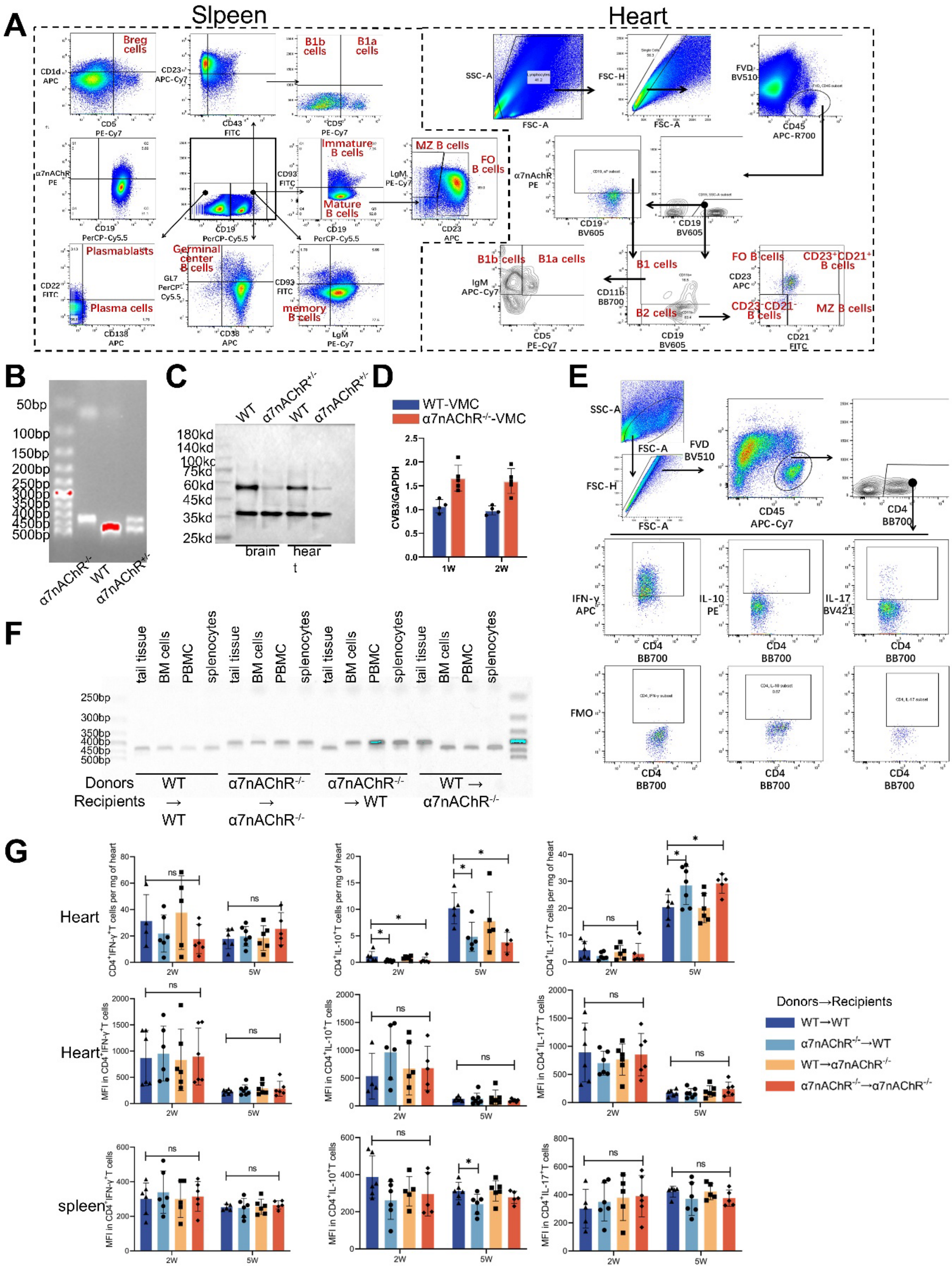

A
Slpeen
Heart
Breg cells
B1b cells
B1a cells
Immature B cells
Mature B cells
MZ B cells
FO B cells
Plasmablasts
Plasma cells
Germinal center B cells
memory B cells
B1b cells
B1a cells
B1 cells
B2 cells
FO B cells
CD23+CD21+ B cells
CD23-CD21- B cells
MZ B cells
B
C
D
E
F
G
WT-VMC
α7nAChR-/--VMC
CVB3/GAPDH
1W
2W
brain
heart
Donors
Recipients
WT → WT
α7nAChR-/- → α7nAChR-/-
α7nAChR-/- → WT
WT → α7nAChR-/-
tail tissue
BM cells
PBMC
splenocytes
Heart
spleen
Donors→Recipients
WT→WT
α7nAChR-/-→WT
WT→α7nAChR-/-
α7nAChR-/-→α7nAChR-/-

Supplemental Fig. 1 (A). Flow cytometry gating strategies of splenic and cardiac B cell subsets were shown. The phenotypic definition of spleen and cardiac B-cell subsets is shown in Supplemental Table 1. **(B)**. Genotyping PCR showing wild type, α7nAChR+/− (heterozygote), and α7nAChR −/− (knockout). **(C)**. The efficiency of α7nAChR knockout mice was identified by WB. In homozygous mice, the protein expression of α7nAChR in B cells was completely inhibited, with brain tissue serving as a positive control. **(D)**. mRNA expression of CVB3 in heart tissue obtained from the VMC and control mice (n= 4 mice/group). **(E)**. Flow cytometry gating strategies of cardiac T cell subsets were shown. **(F)**. Genotyping PCR showed that bone marrow cells, PBMCs, and splenic cells from the recipient had the same genotype as the donor after successful bone marrow transplantation. **(G)**. A summary graph shows the quantification of the number of IFN-γ, IL-10 and IL-17-producing cardiac T cells per mg of heart in chimeric mice; And statistical graphs showing the mean fluorescence intensity(MFI) of Ki-67 expressed in IFN-γ, IL-10 and IL-17-producing cardiac and splenic T cells from chimeric mice (n= at least 5 per group).The data are analyzed using one-way ANOVA (G) and represented as the means±SD. *p<0.05,**p<0.01,***p<0.001 between groups.

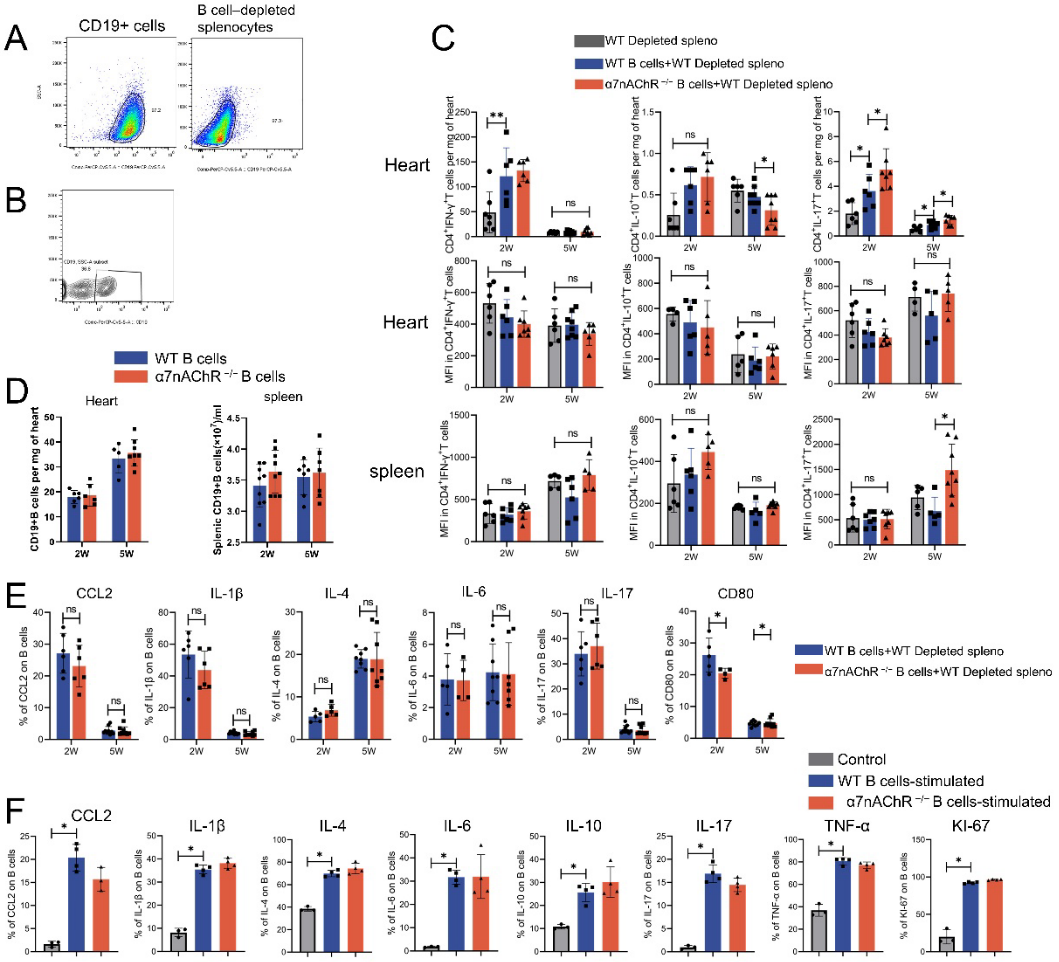

Supplemental Fig.2 (A). The identification of flow cytometry revealed that CD19$^+$ cells had a purity >97% and B cell–depleted splenocytes had a purity >97%. **(B)**. Successful reconstitution of splenic B cells was confirmed by flow cytometry. **(C)**. Summary graph showing quantification of the number of IFN-γ, IL-10 and IL-17-producing cardiac T cells per mg of heart in SCID mice with immune reconstitution; And statistical graphs showing the mean fluorescence intensity(MFI) of Ki-67 expressed in IFN-γ, IL-10 and IL-17-producing cardiac and splenic T cells from SCID mice with immune reconstitution (n= at least 5 mice/group). **(D)**. The absolute number of splenic and cardiac B cells between recipient mice that received WT B lymphocytes and α7nAChR$^{-/-}$ B lymphocytes at weeks 2 and 5 of VMC are shown (n= at least 5-8 mice/group). **(E)**. Quantification of the percentages of CCL2, IL-1β,IL-4, IL-6, IL-17, and CD80 on gated CD19$^+$B cells from recipient spleens are shown (n= at least 5 mice/group).**(F)**. Quantification of the percentages of CCL2, IL-1β,IL-4, IL-6, IL-10, IL-17, TNF-α and Ki-67 on CD19$^+$B cells cultured in vitro (n= 3-4). The results from (F) are pooled from at least three independent experiments. The data are represented as

the means ±SD. **p*<0.05,***p*<0.01,****p*<0.001 calculated with two-tailed paired t test (D and E) and one-way ANOVA(C and F).

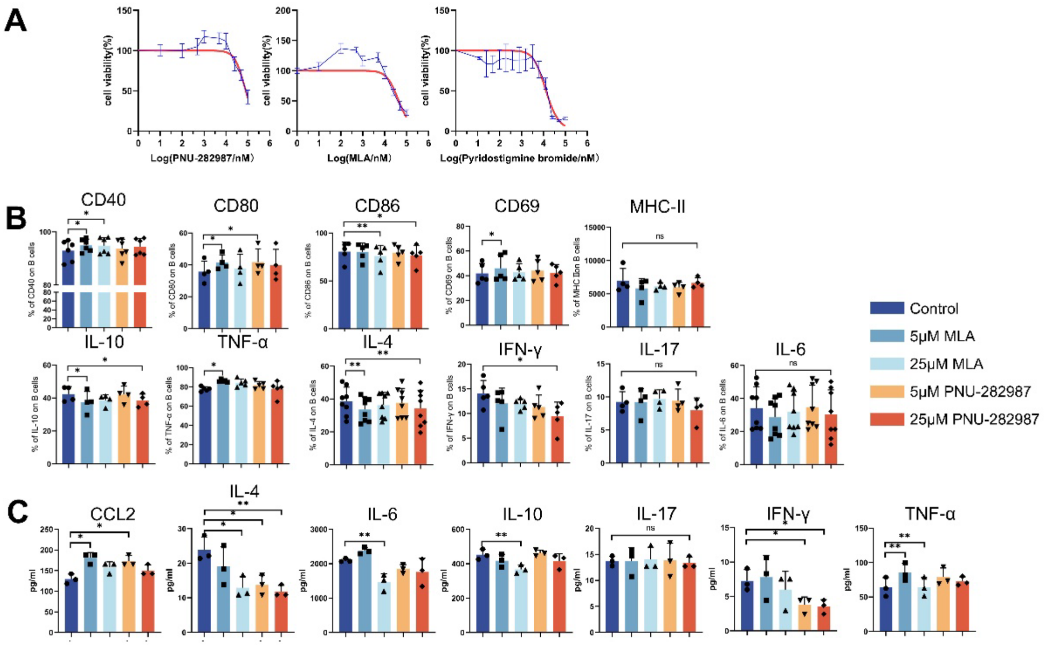

Supplemental Fig.3 (A). The viability of B cells and fitting curve were measured by the CCK8 assay at all concentrations from 100 nM until 100 μM PNU-282987, methyllycaconitine(MLA), and pyridostigmine bromide (PYR) .**(B)**. Quantification of the percentages of CD40,CD80, CD86, CD69, MHC-II, IL-10, TNF-α, IL-4, IFN-γ, IL-17, and IL-6 on $CD19^+$B cells treated with MLA and PNU-282987 (n= 4-6). **(C)**. The protein concentration of CCL2 , IL-4, IL-6, IL-10, IL-17, IFN-γ, and TNF-α in B cell culture supernatant was quantified using the Luminex assay after treatment with methyllycaconitine(MLA) and PNU-282987 (n= 4-6).The results from (A-C) are pooled from at least three independent experiments. The data are represented as the means ±SD. **p*<0.05,***p*<0.01,****p*<0.001 calculated with one-way ANOVA(B and C).Non-linear regression(A) were used.

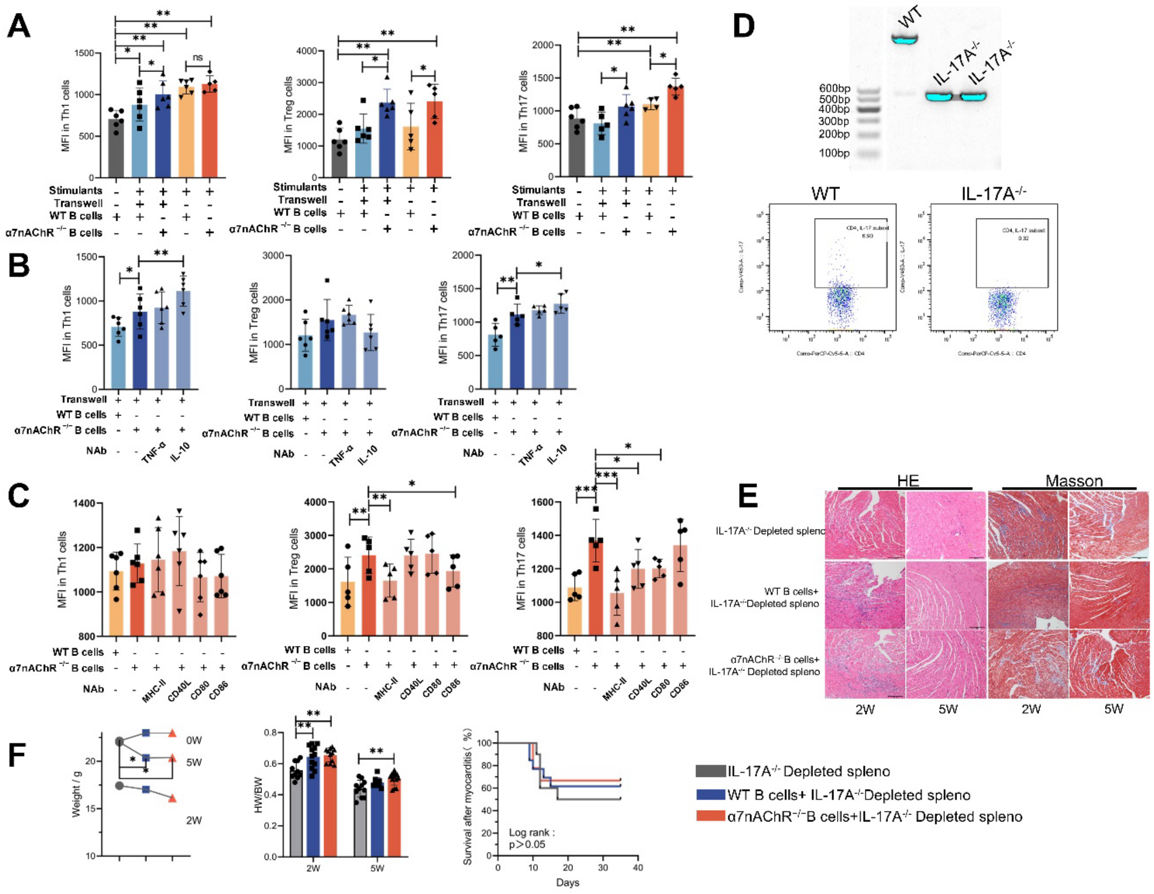

Supplemental Fig.4 (A). Quantitation of Ki-67 mean fluorescence intensity(MFI) in IFN-γ, IL-10 and IL-17-producing splenic T cells cultured in in vitro co-culture system. Neutralizing antibodies anti-IL-10 and anti-TNF-α were added to the transwell co-culture system **(B)** and anti-MHC-Ⅱ, anti-CD40, anti-CD80, and anti-CD86 were added to the direct co-culture system **(C)**; The Ki-67 expression of spleen T cells that produce IFN-γ, IL-10 and il-17 in vitro were observed. The results from (A-C) are pooled from at least three independent experiments. **(D)**. PCR and flow cytometry were used to determine the efficiency of IL-17A knockout. Genotyping PCR showing wild type and IL-17A $^{-/-}$ (knockout). T cells lost the ability to secrete IL-17 upon stimulation with PMA, ionomycin, and BFA. **(E)**. Representative images of HE and masson staining of heart sections. at 40× magnification. Scale bar= 200 μM. **(F).** The body weight loss, survival, and HW/BW of each group was monitored over time (n= 10-11 mice/group).The data are represented as the means ±SD.*p<0.05,**p<0.01,***p<0.001 calculated with Log-rank (Mantel–Cox) test (F) and one-way ANOVA(A,B,C, and F).

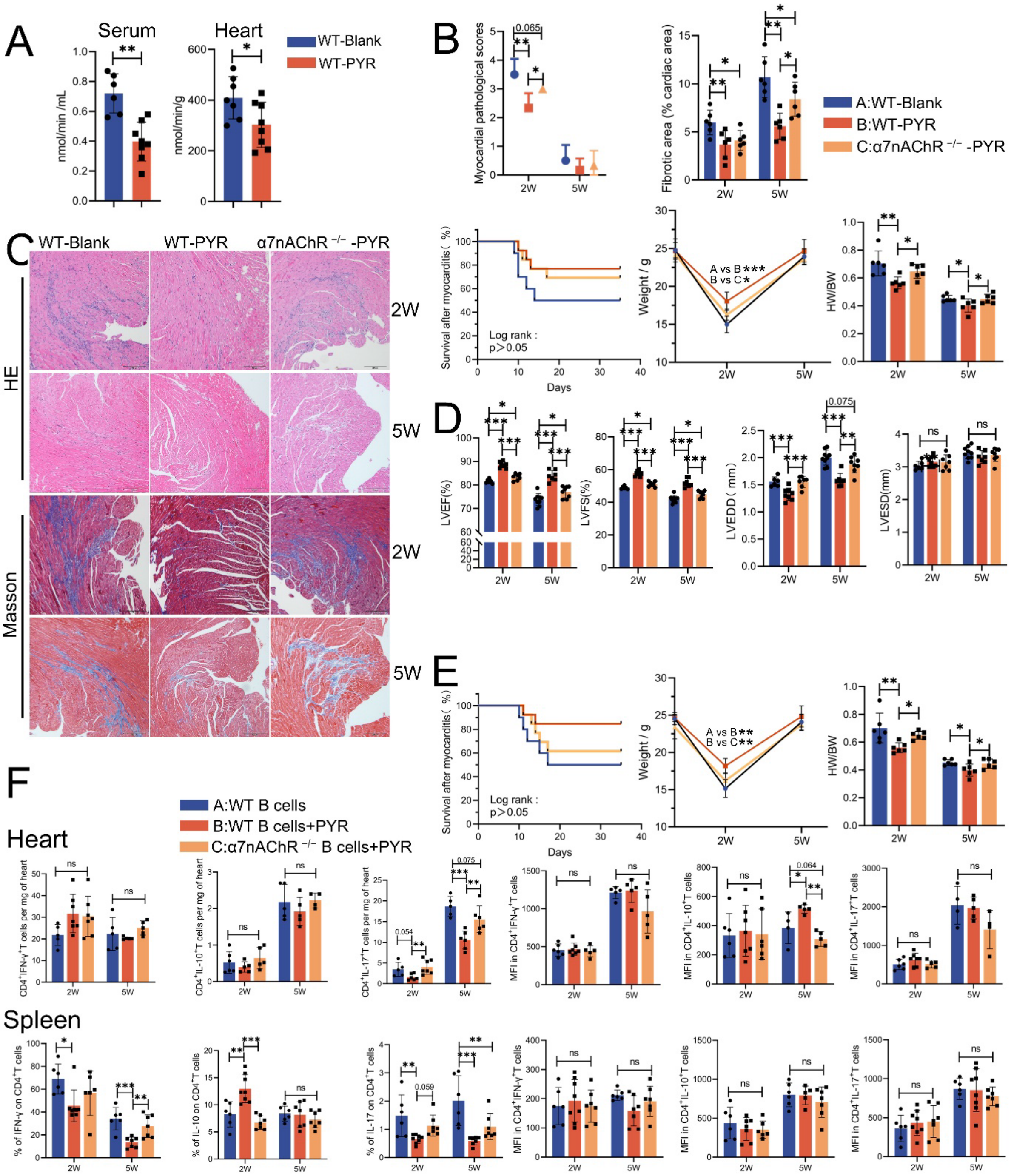

Supplemental Fig.5 (A). After pyridostigmine bromide (PYR) treatment, the protein levels of acetylcholinesterase (AChE) in serum and myocardial tissue homogenate supernatant were detected by colorimetric assay(n= 6-9 mice/group). After infection with CVB3, WT and α7nAChR −/− mice were fed either normal drinking water or 30mg/kg/day pyridostigmine bromide (PYR) daily until the endpoint of observation. **(B)**. Tissue damage and myocardial fibrosis was evaluated by HE and masson staining, and the body weight loss, survival, and HW/BW of each group was monitored over

time (n= 6 mice/group). **(C)**. Representative images of HE and masson staining of hearts from PY-intervened WT and α7nAChR −/− mice were shown. Scale bar= 200 μM. **(D)**. Cardiac function of blank and PYR group were measured (n= 6 mice/group). **(E)**. The body weight loss, survival, and HW/BW of SCID mice was monitored over time (n= 6 mice/group). **(F)**. Summary graph showing quantification of the number of IFN-γ, IL-10 and IL-17-producing cardiac T cells per mg of heart in SCID mice treated with or without PYR; And statistical graphs showing the mean fluorescence intensity(MFI) of Ki-67 expressed in IFN-γ, IL-10 and IL-17-producing cardiac and splenic T cells from SCID mice treated with or without PYR (n= at least 5 mice/group). The data are represented as the means ±SD. *p<0.05,**p<0.01,***p<0.001 calculated with Log-rank (Mantel–Cox) test (B and E), two-tailed unpaired t test(A), and one-way ANOVA(B-F).
