# Supplementary material for: α7nAChR on B cells directs T cell differentiation to prevent viral myocarditis
Source: JCI Insight. 2025 May 8;10(9):e189323. doi: 10.1172/jci.insight.189323 (PMC12128988; doi:10.1172/jci.insight.189323)

Fig.7A

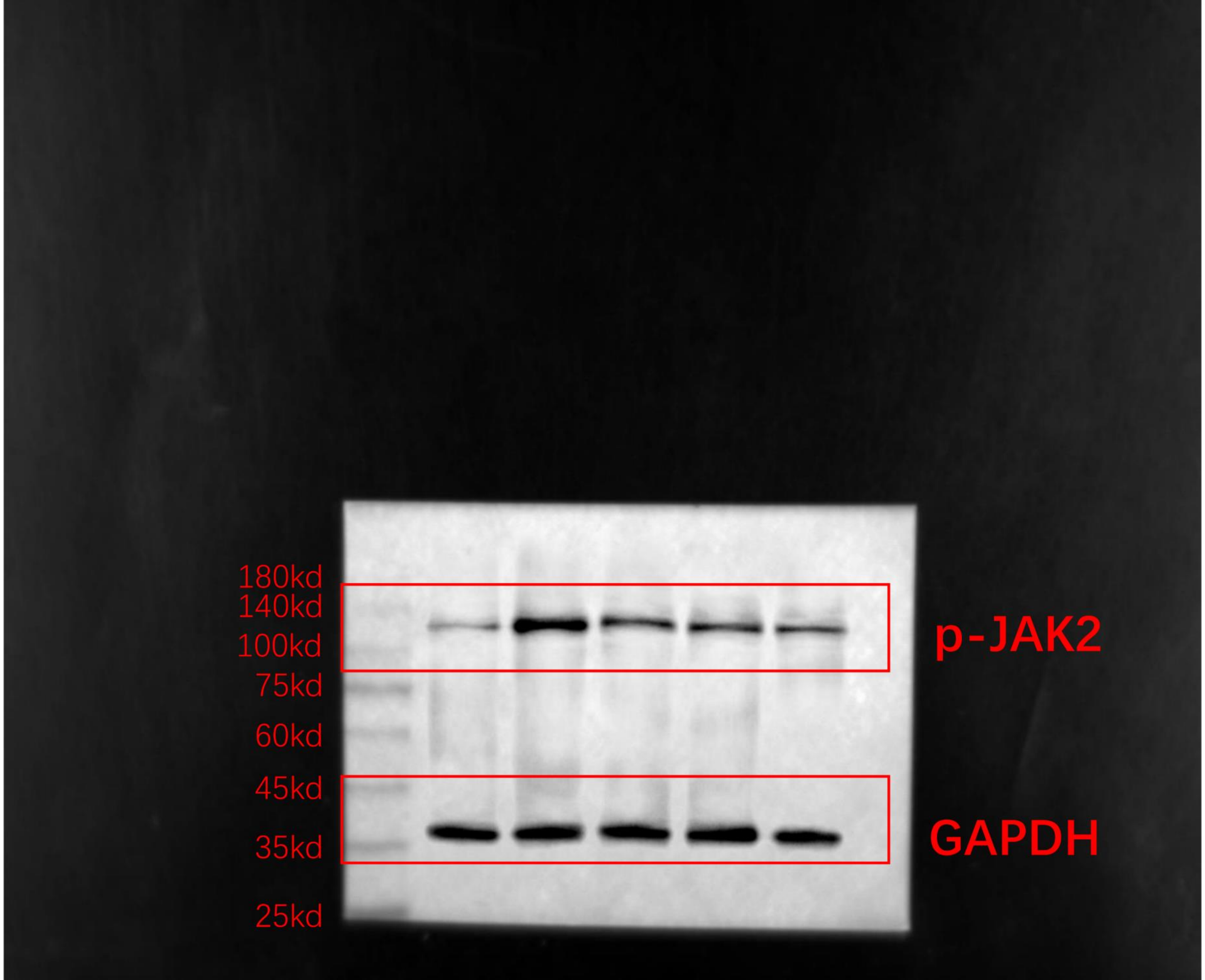

Fig.7A

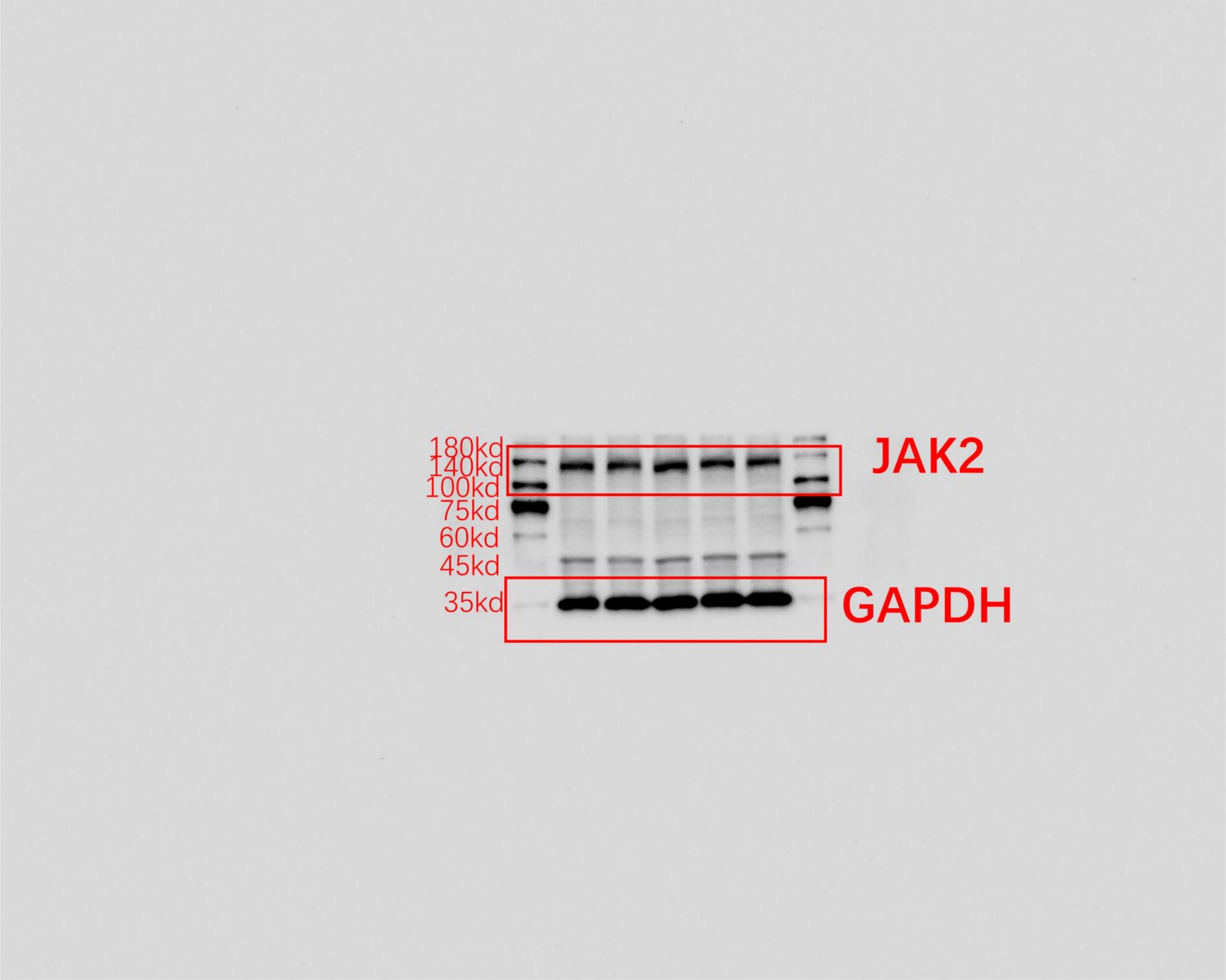

Fig.7A

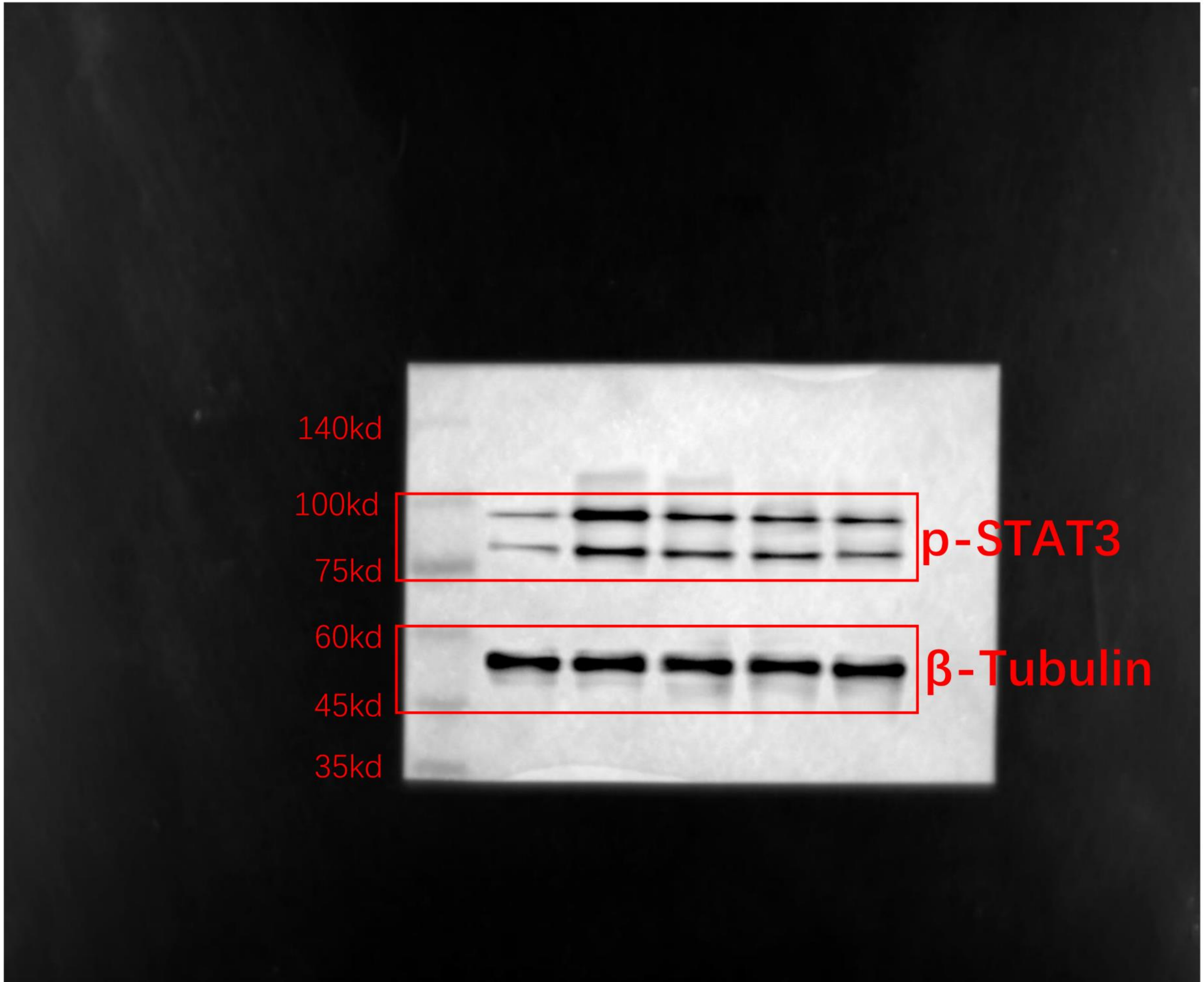

Fig.7A

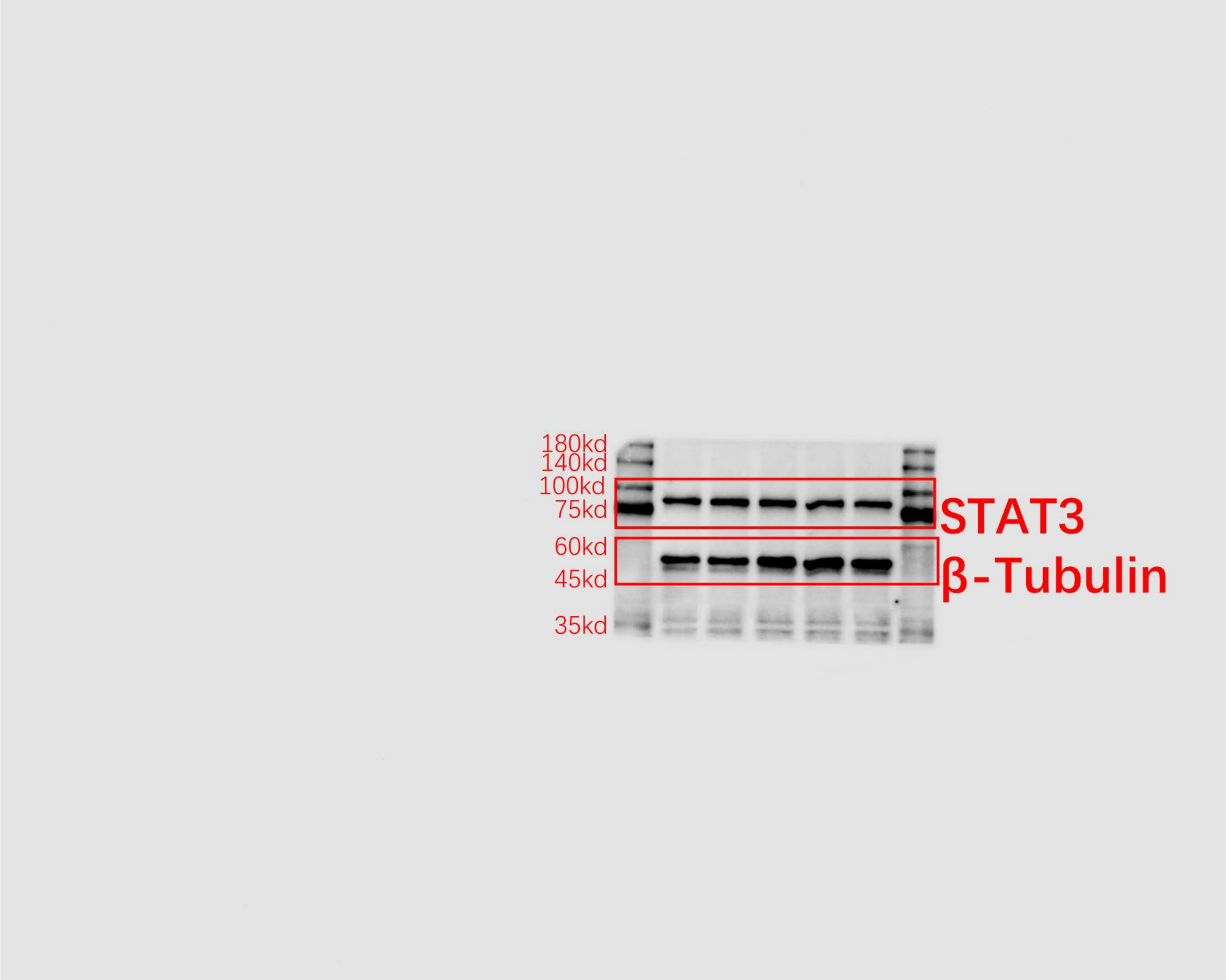

Fig.7A

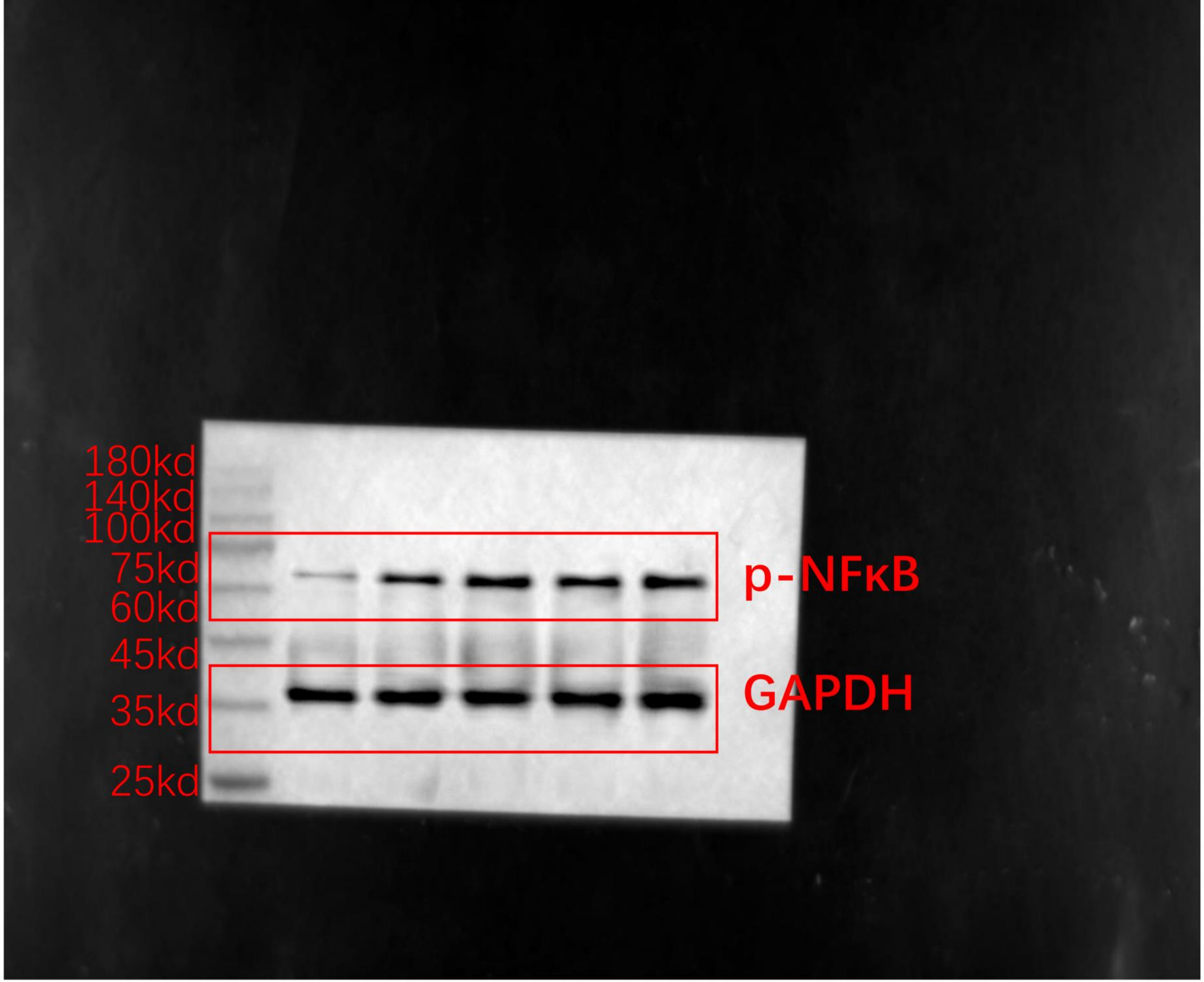

Fig.7A

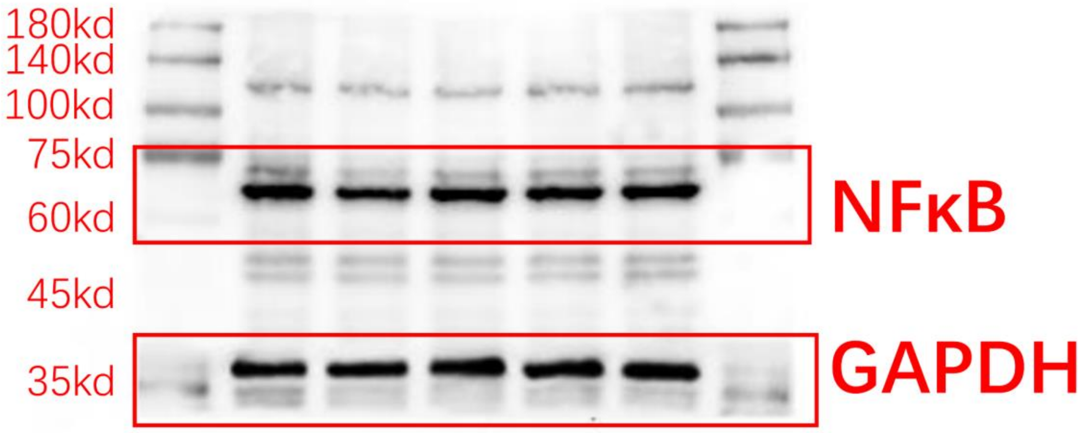

Fig.7A

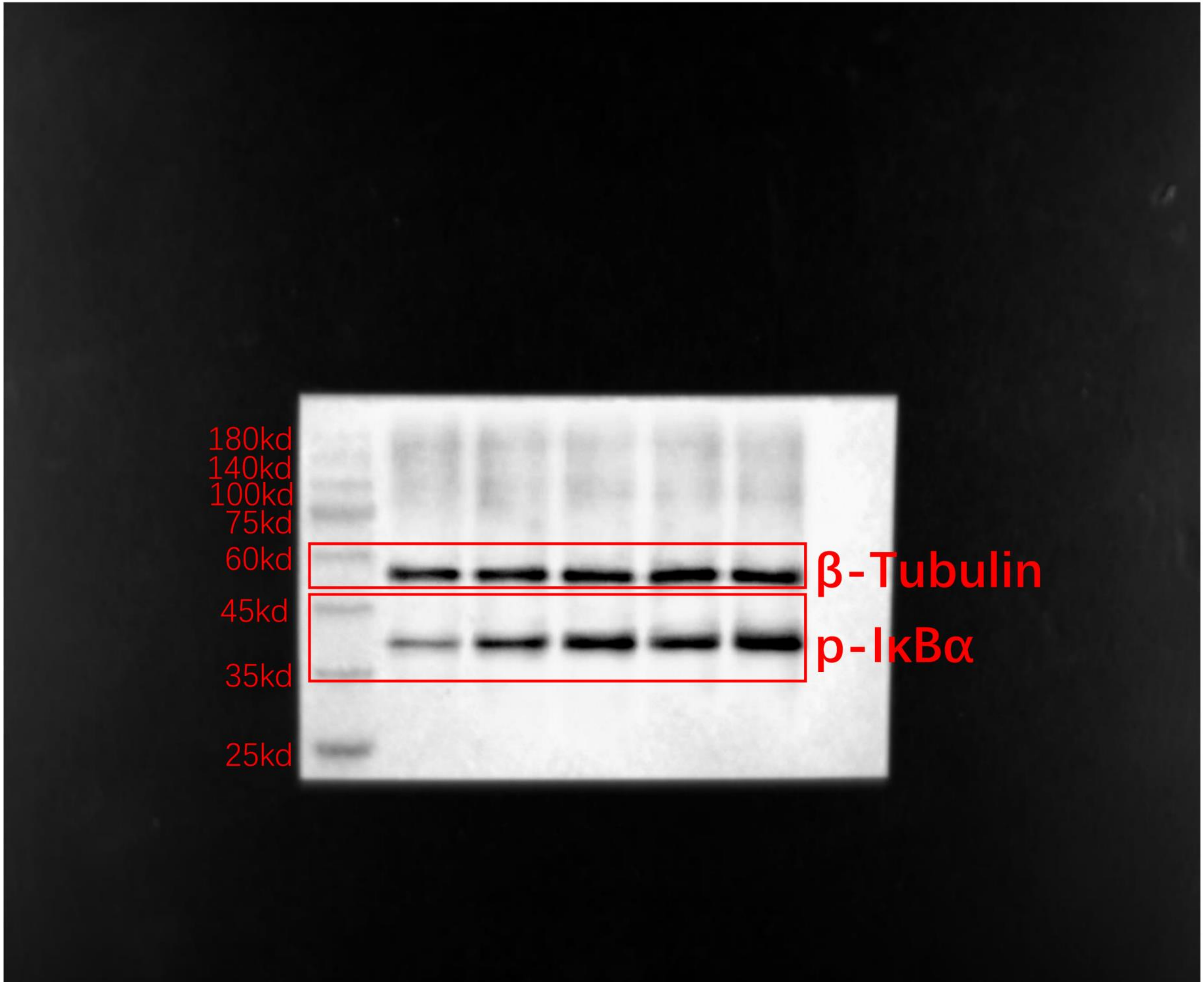

Fig.7A

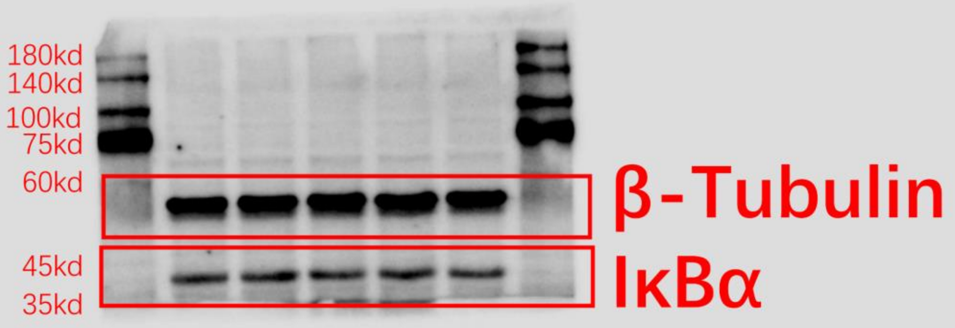

Fig.7A

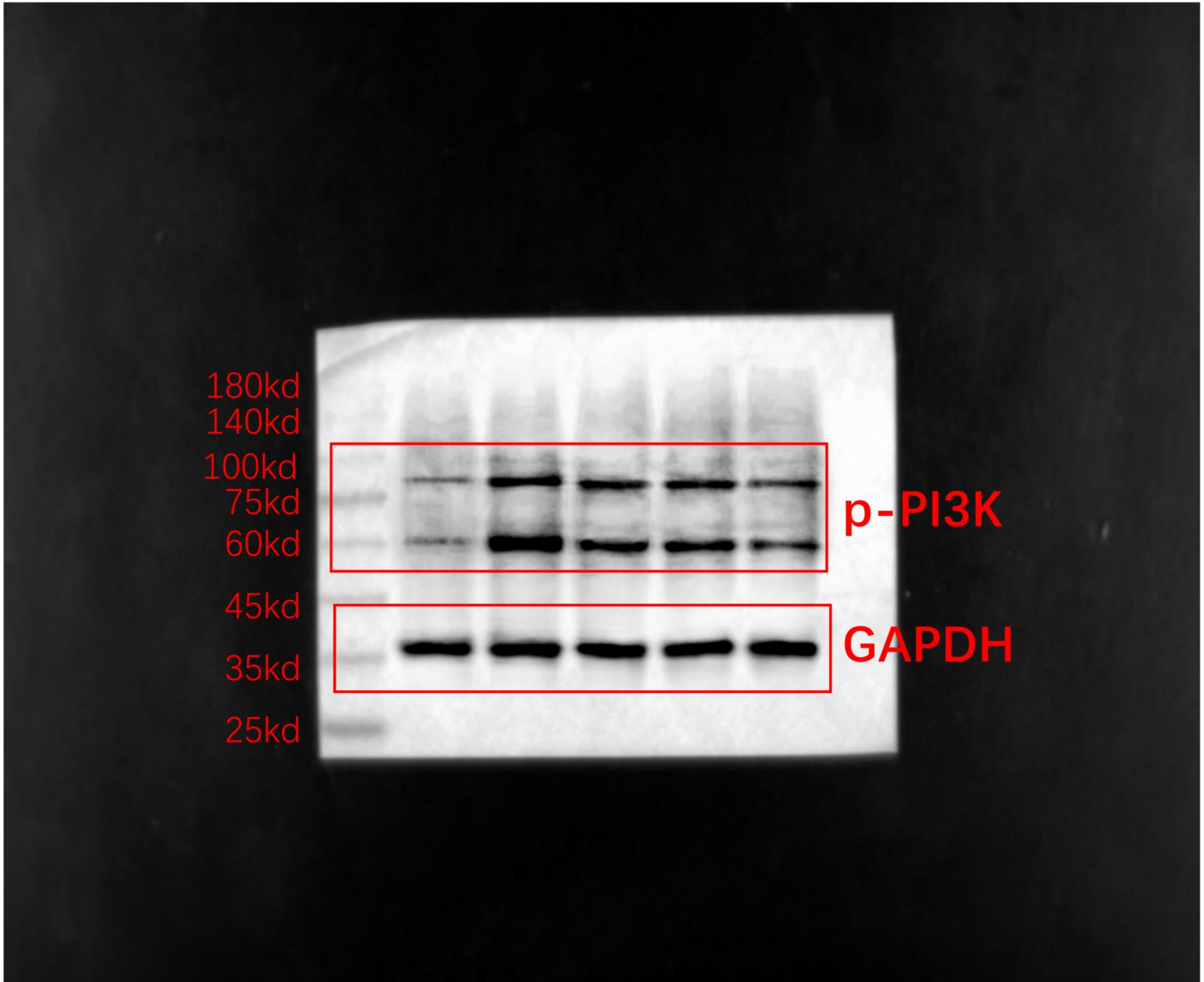

Fig.7A

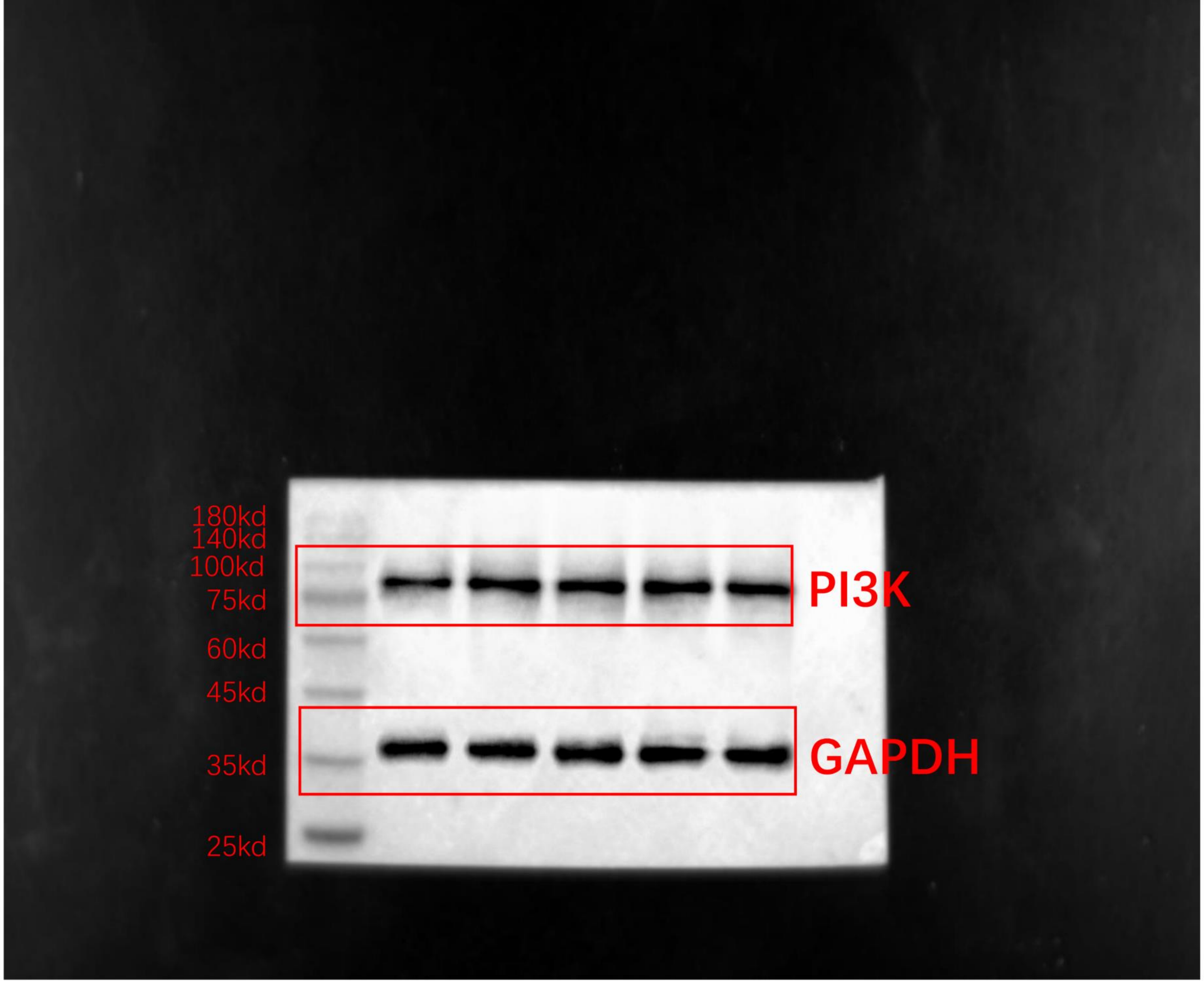

Fig.7A

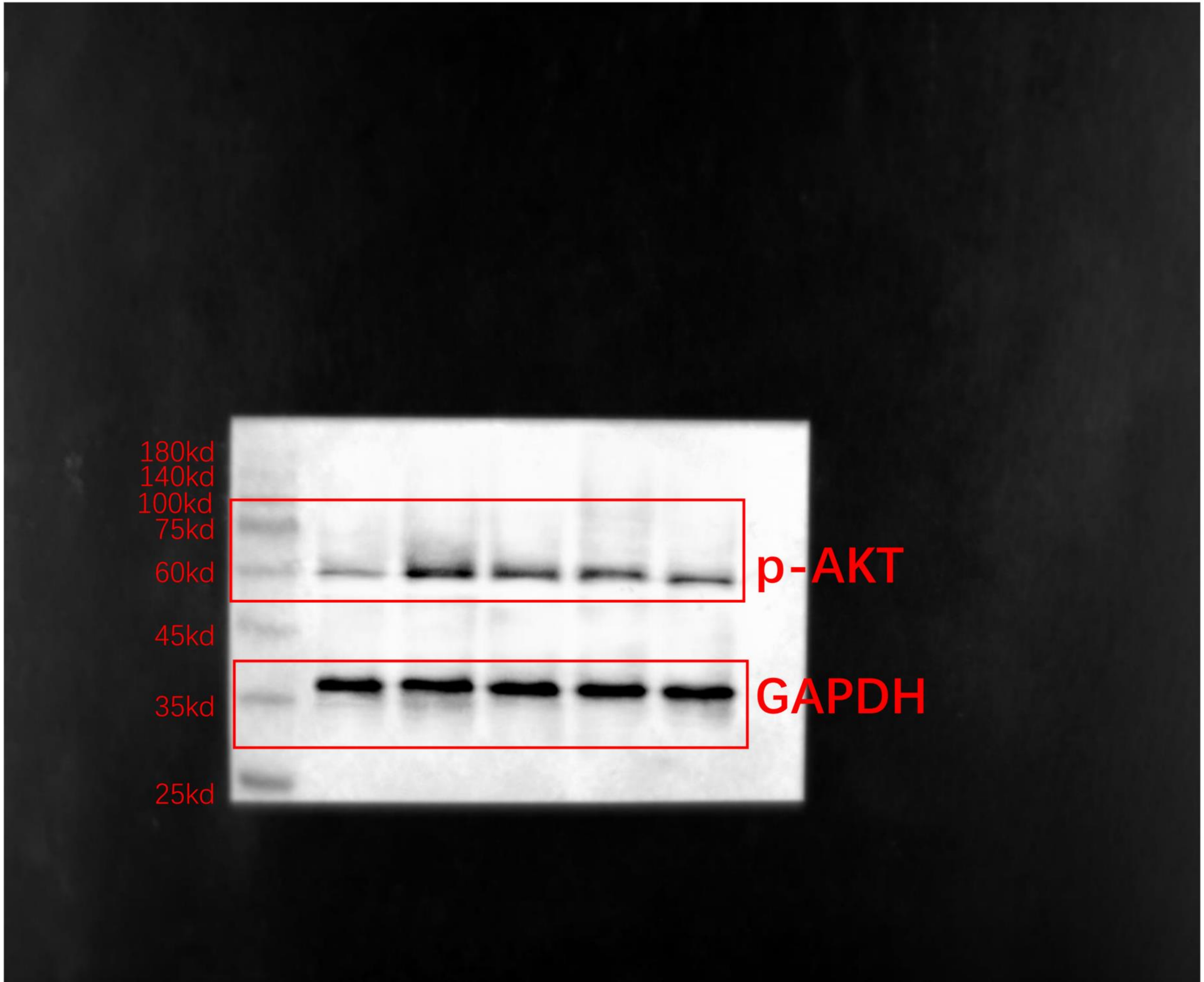

Fig.7B

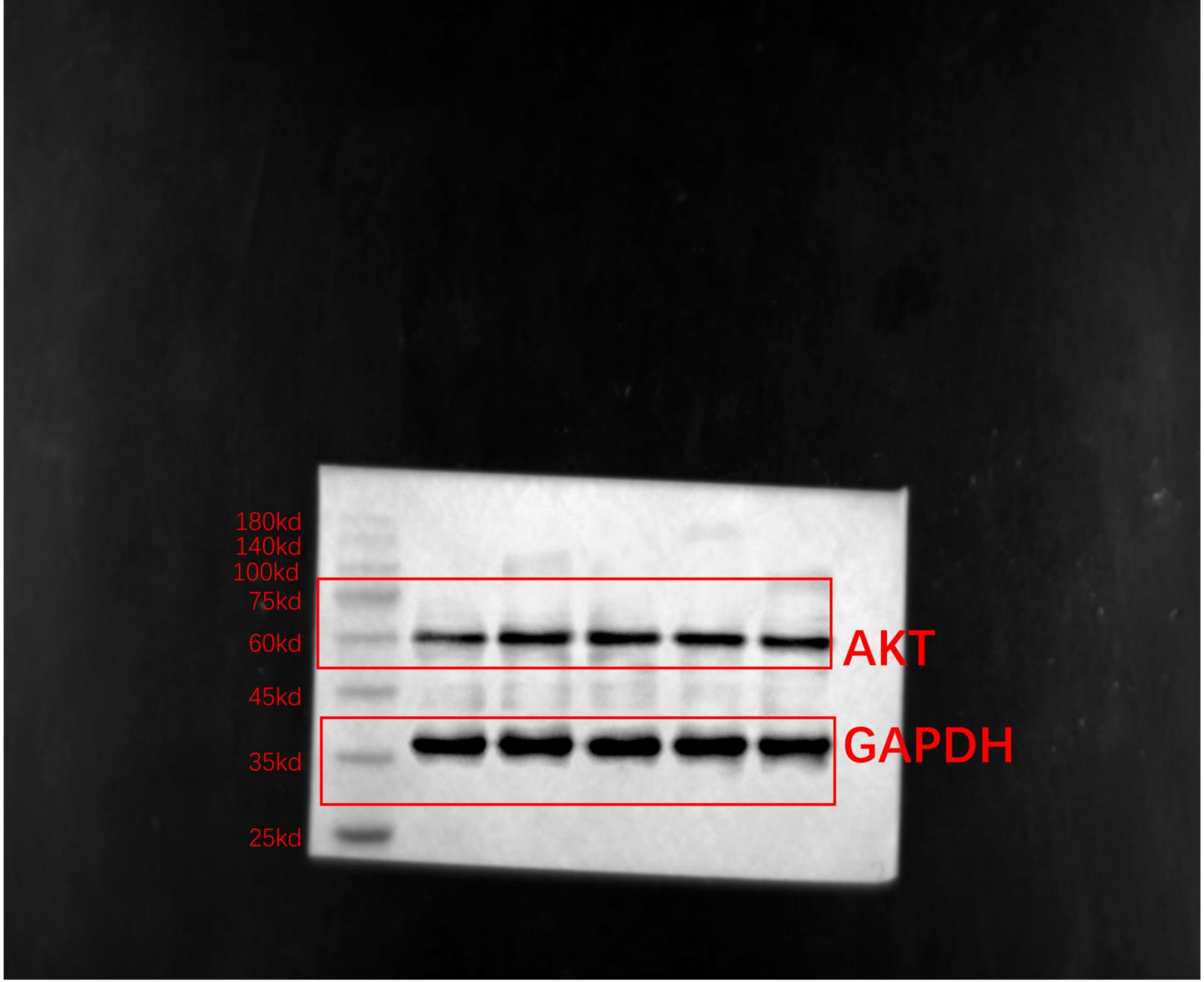

Fig.7B

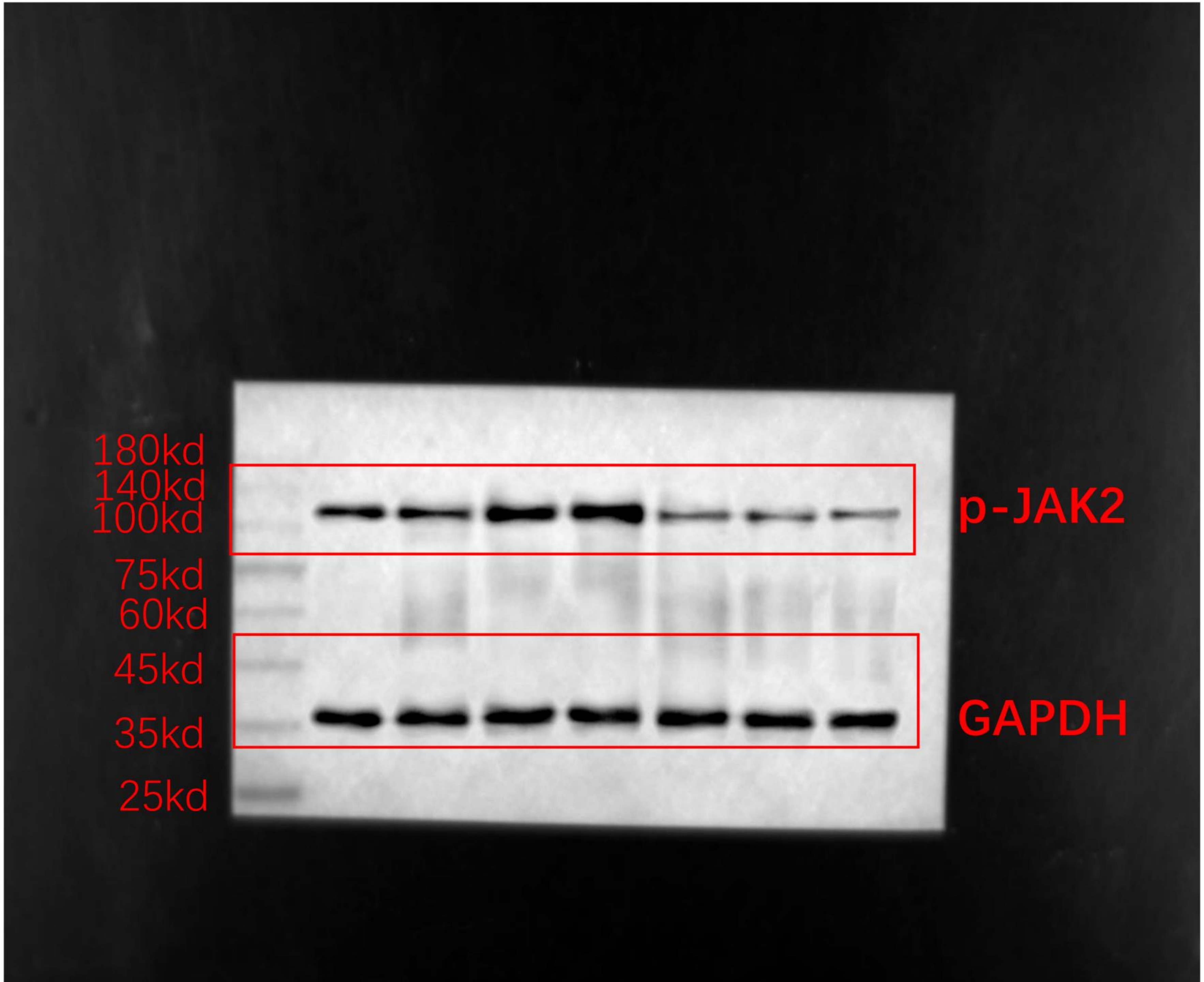

Fig.7B

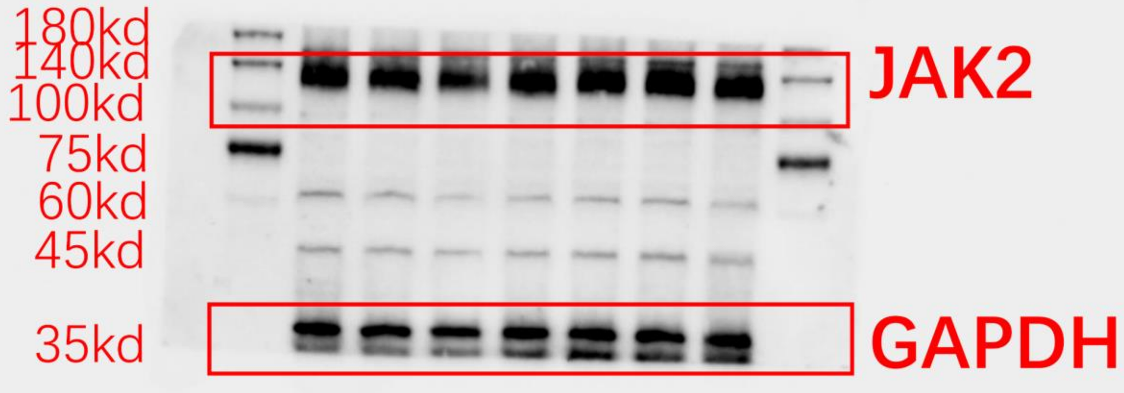

Fig.7B

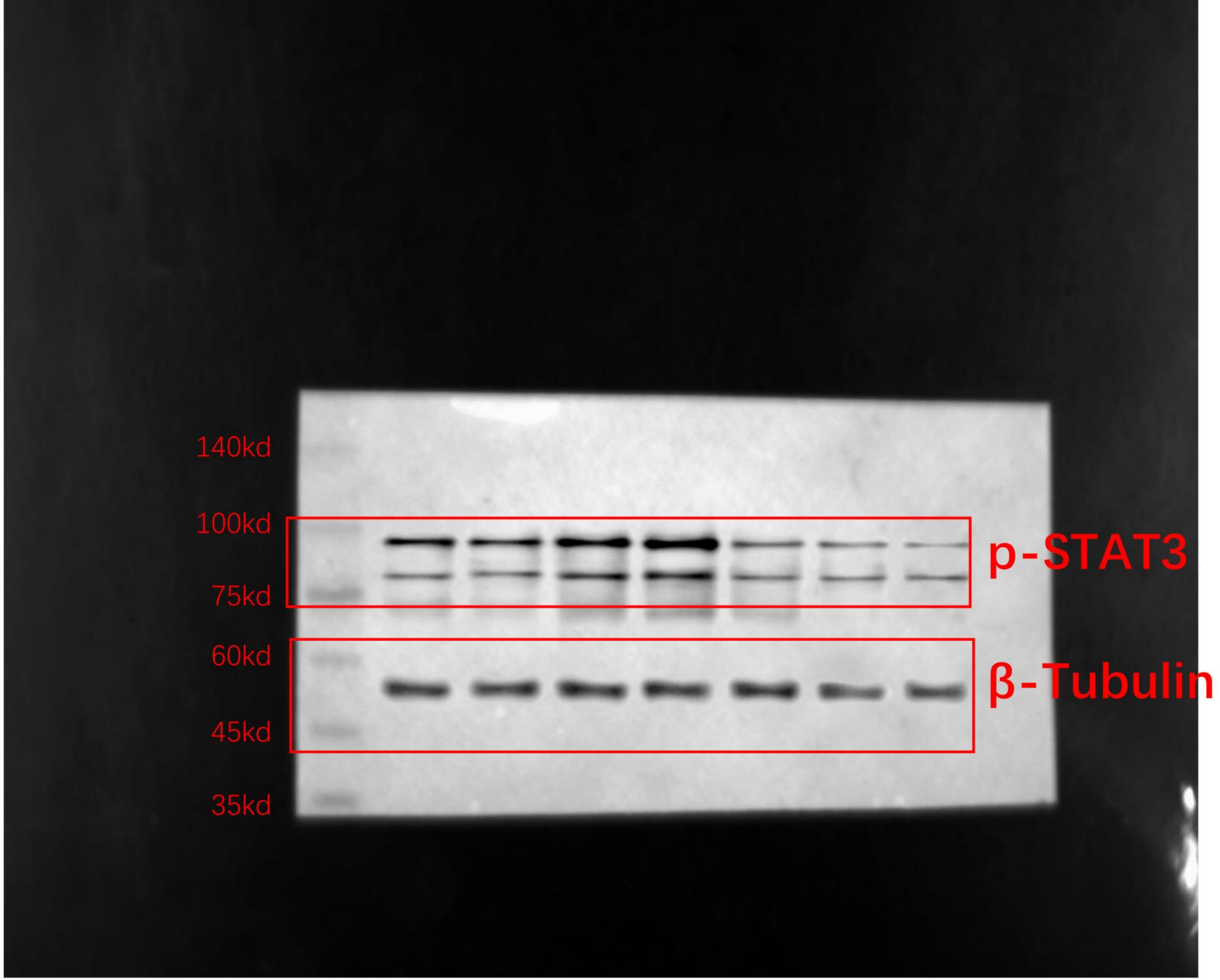

Fig.7B

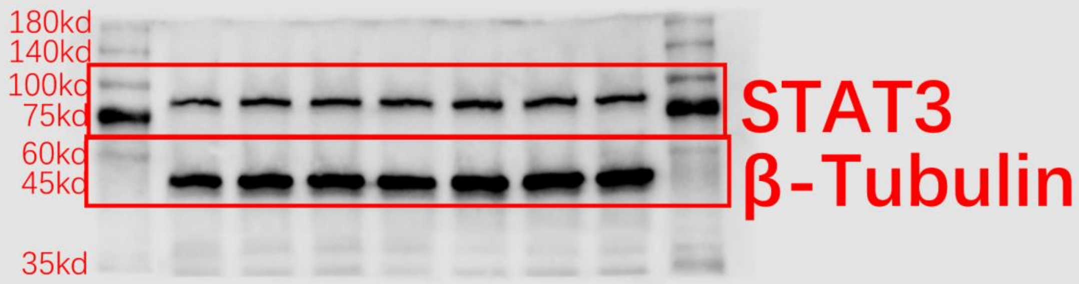

Fig.7B

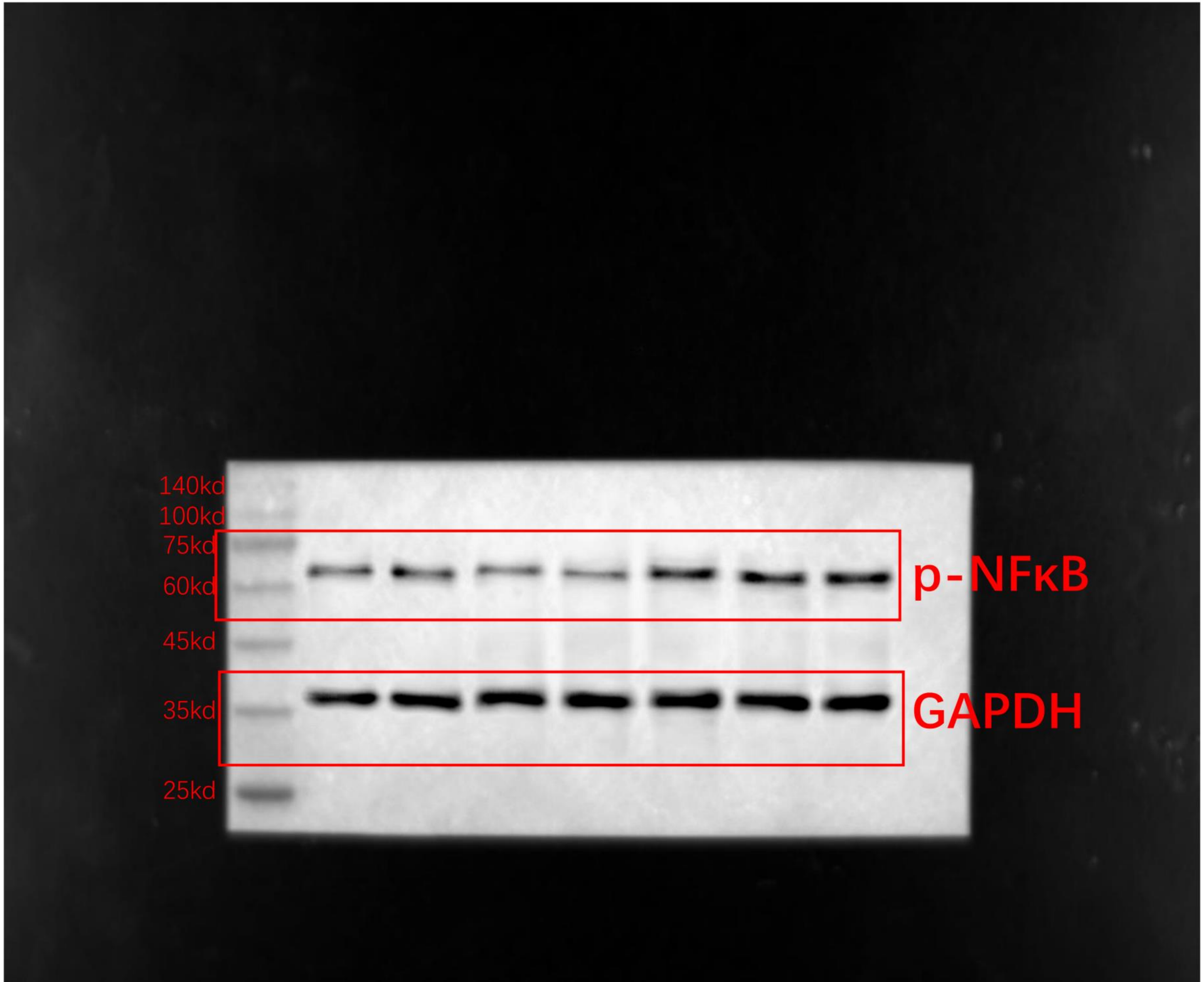

Fig.7B

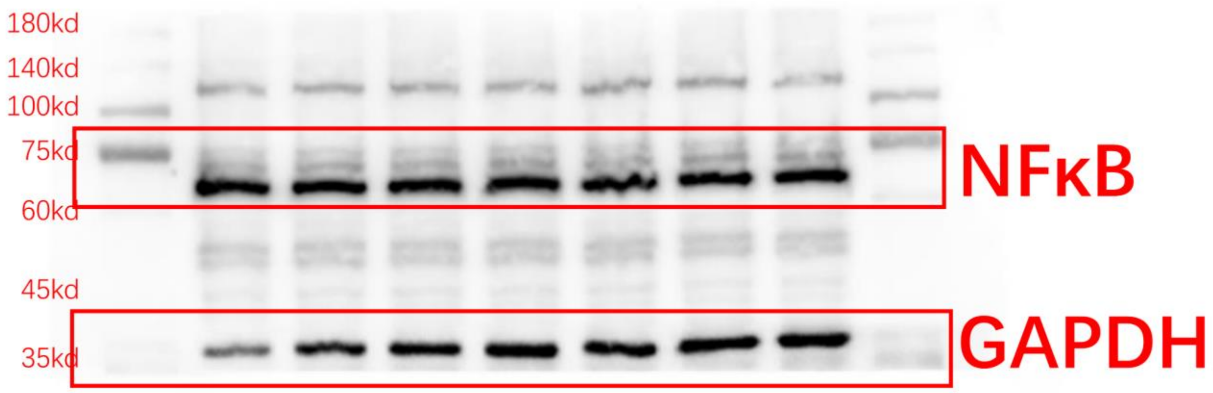

Fig.7B

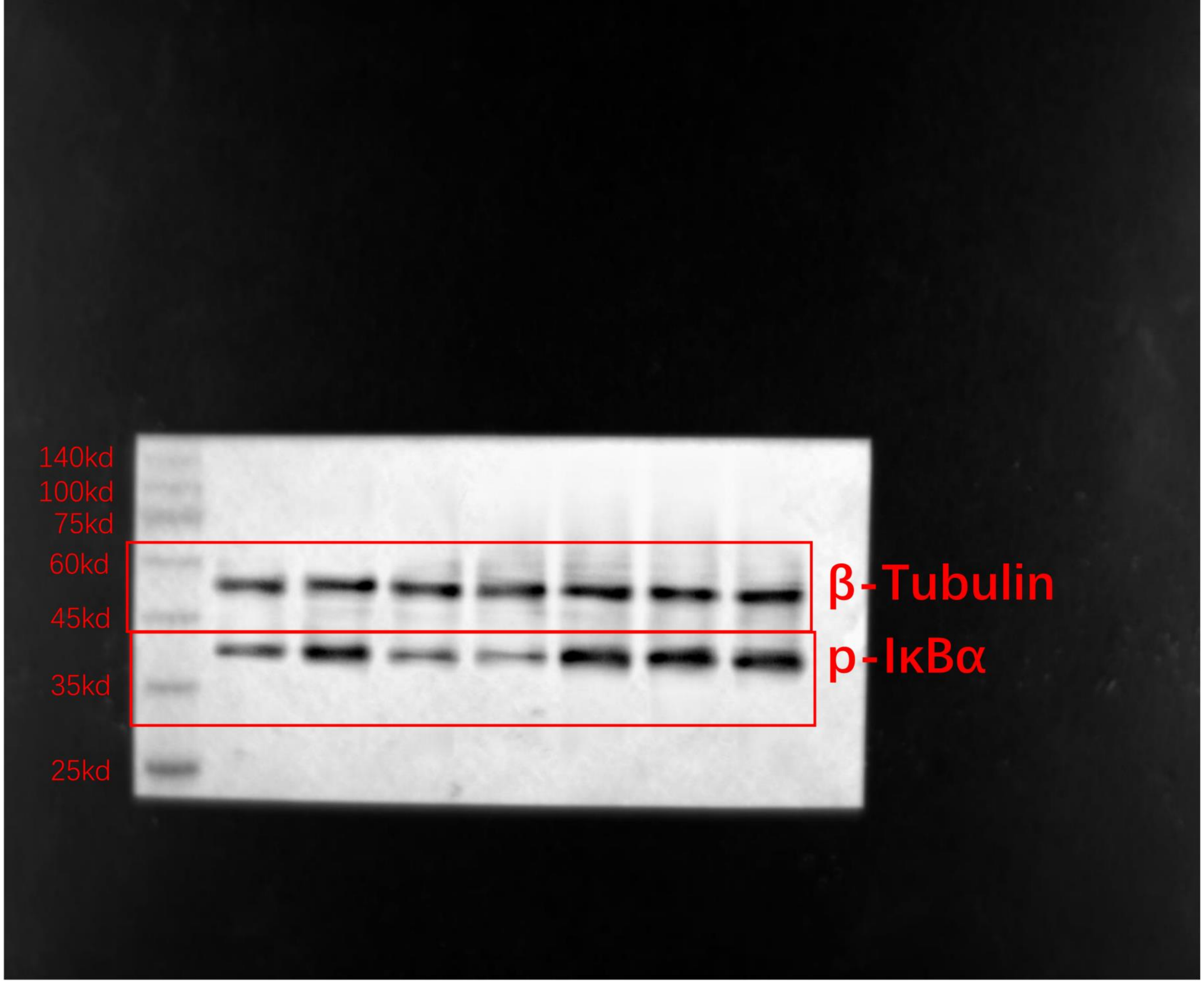

Fig.7B

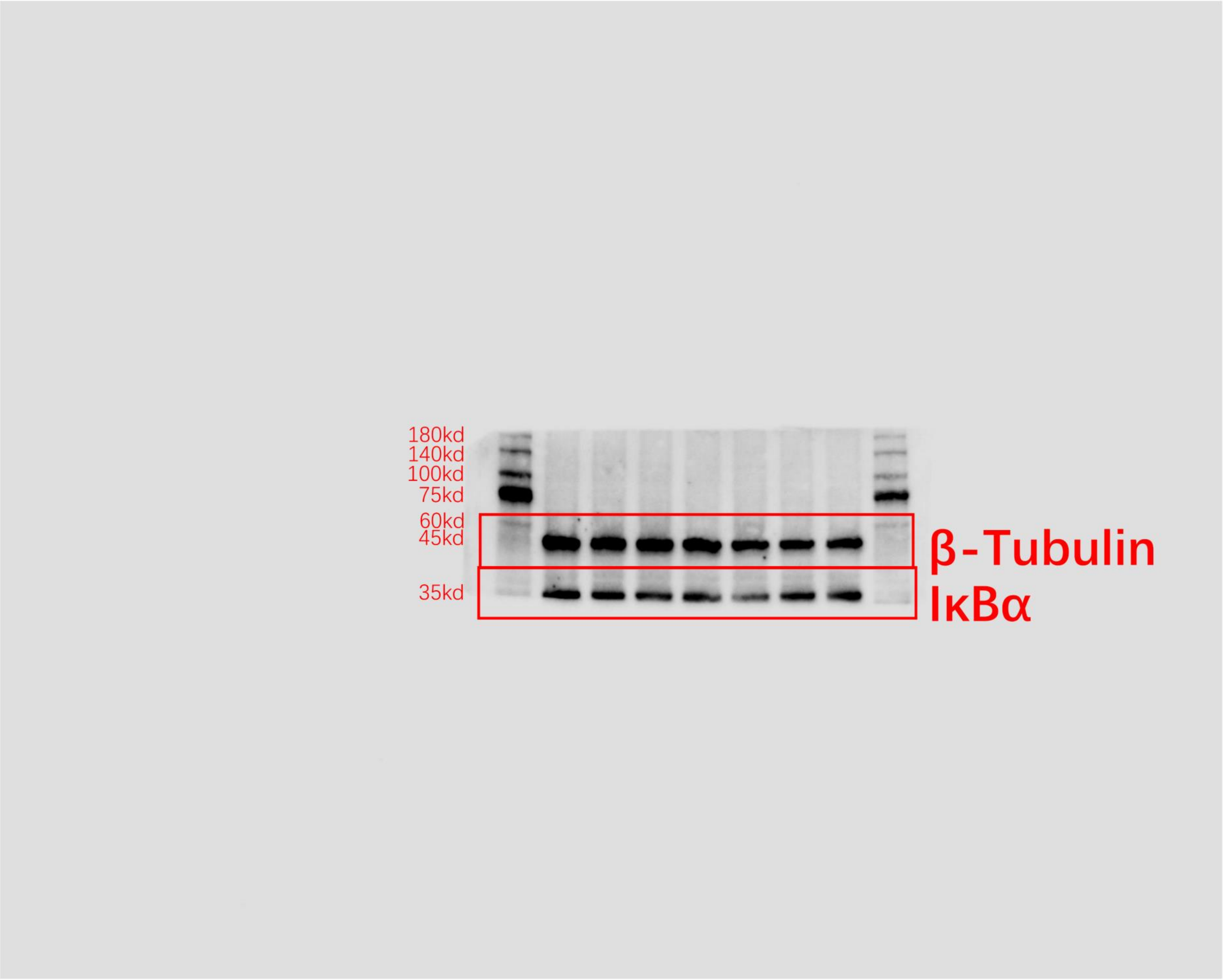

Fig.7B

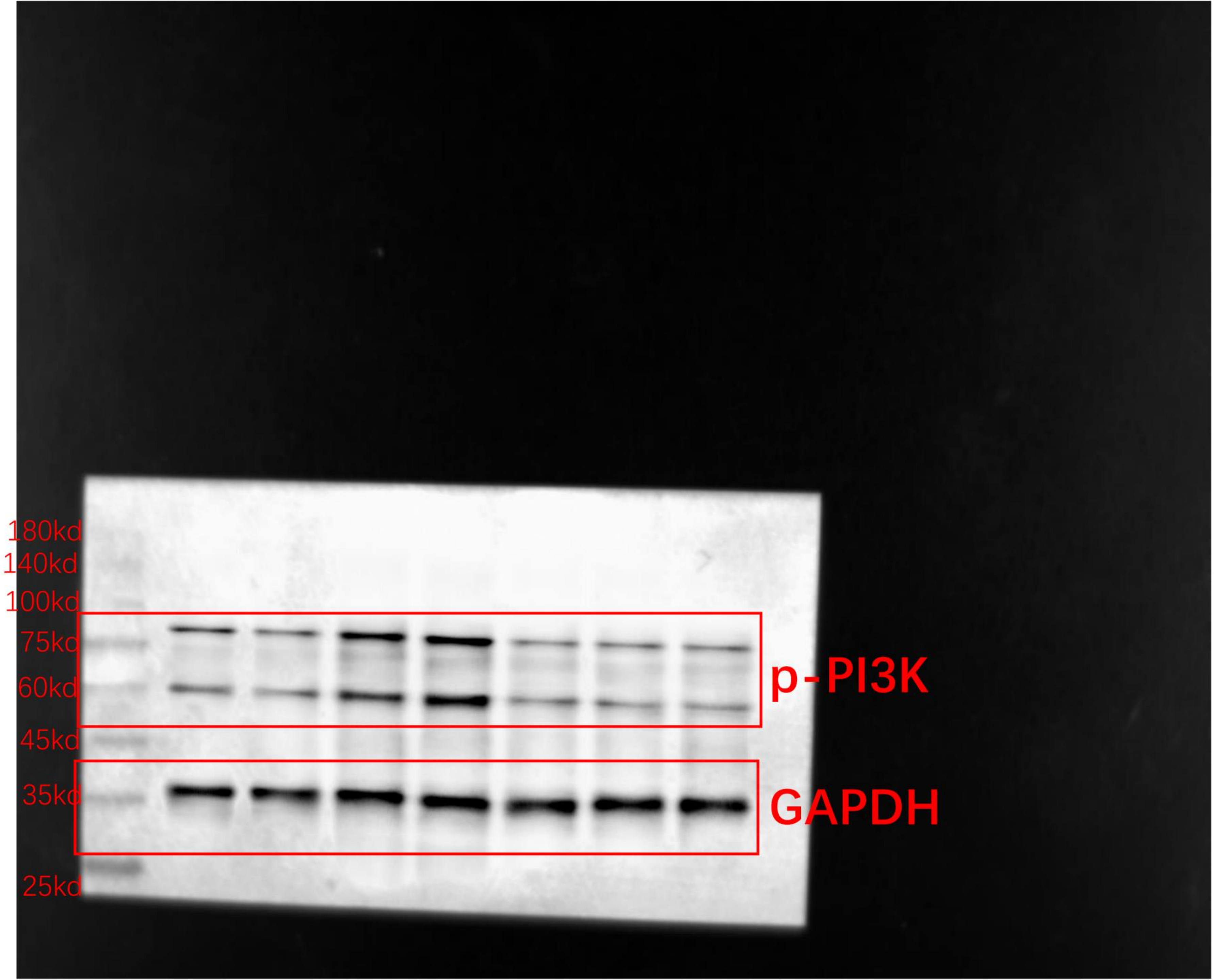

Fig.7B

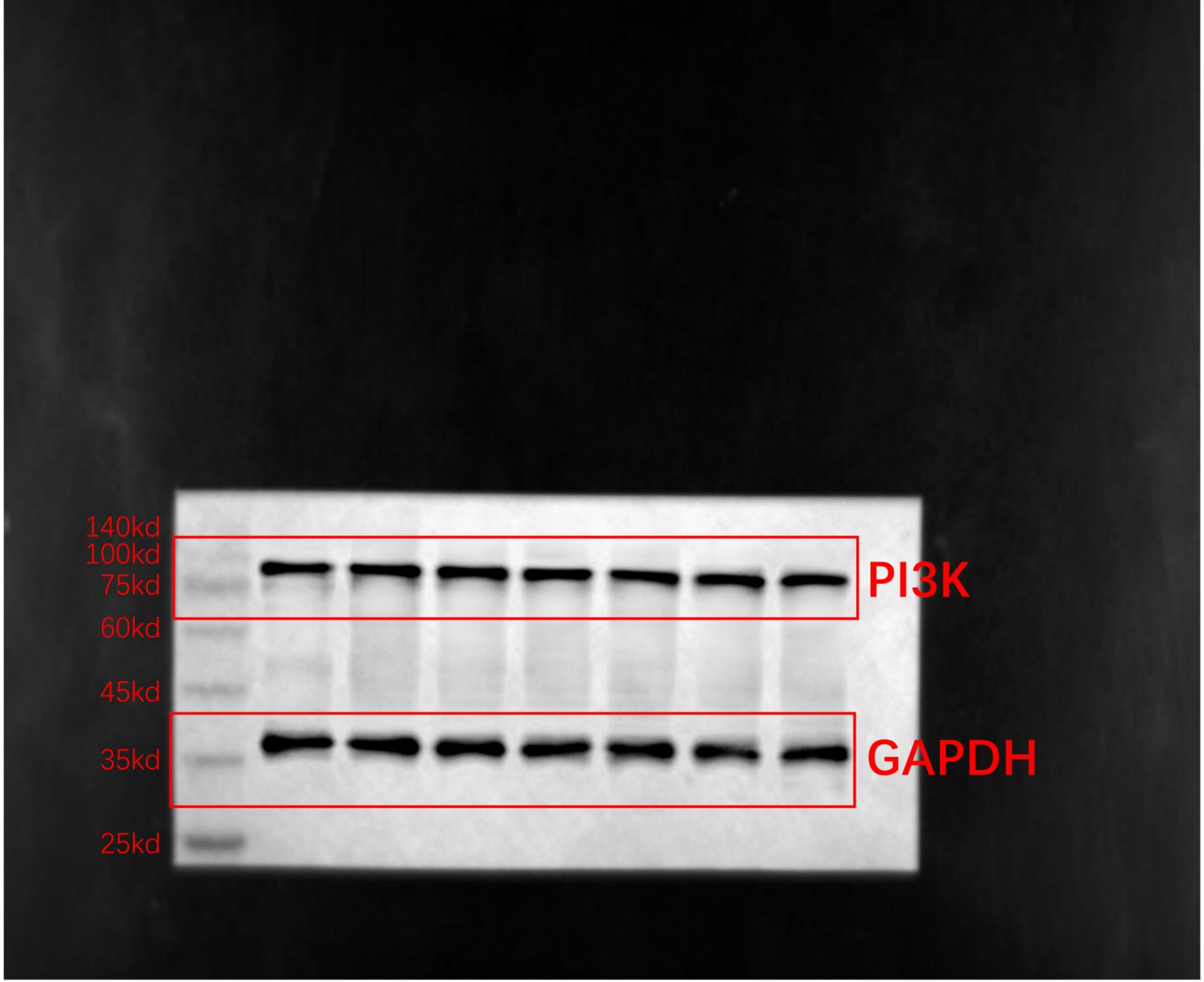

Fig.7B

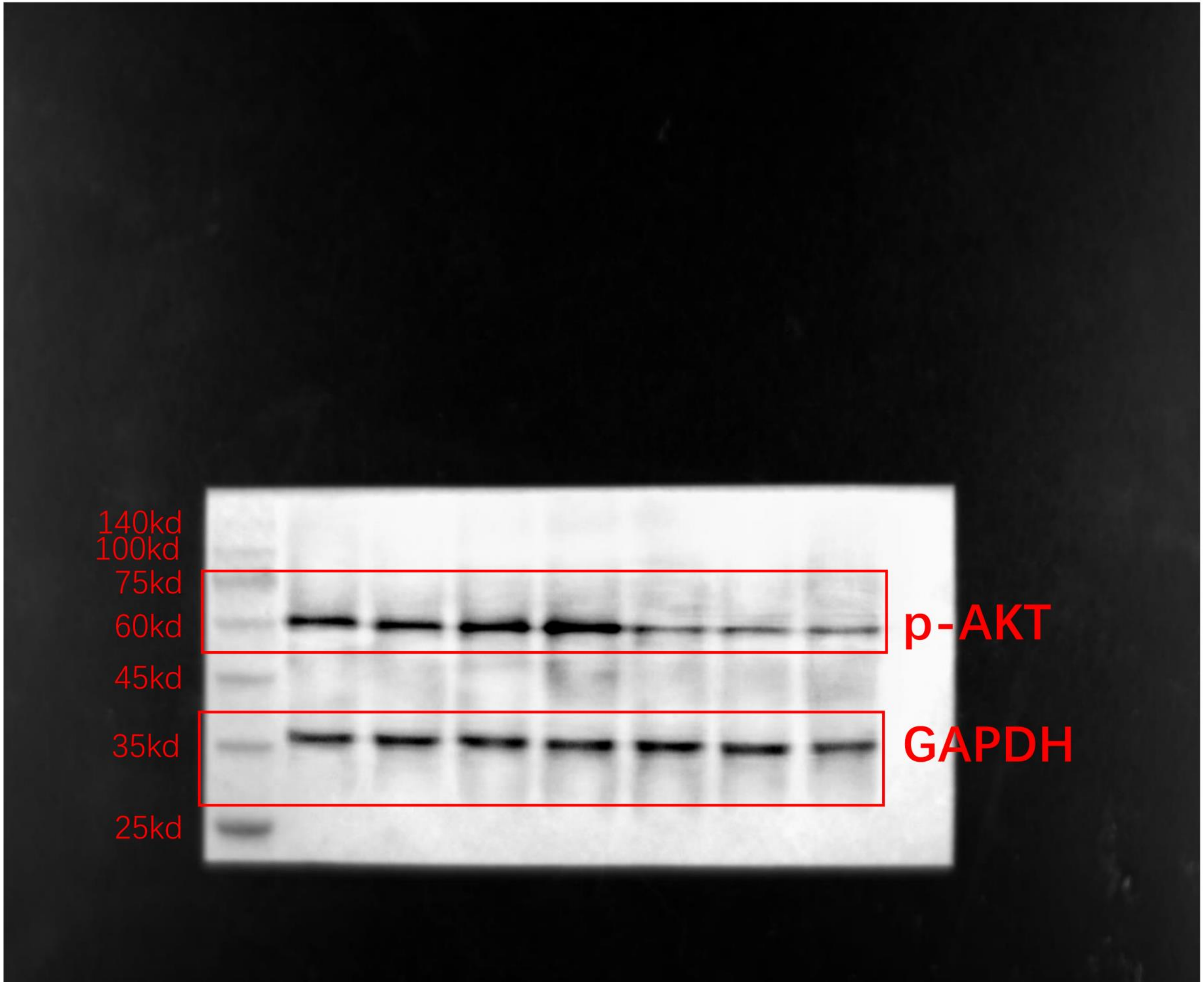

Fig.7B

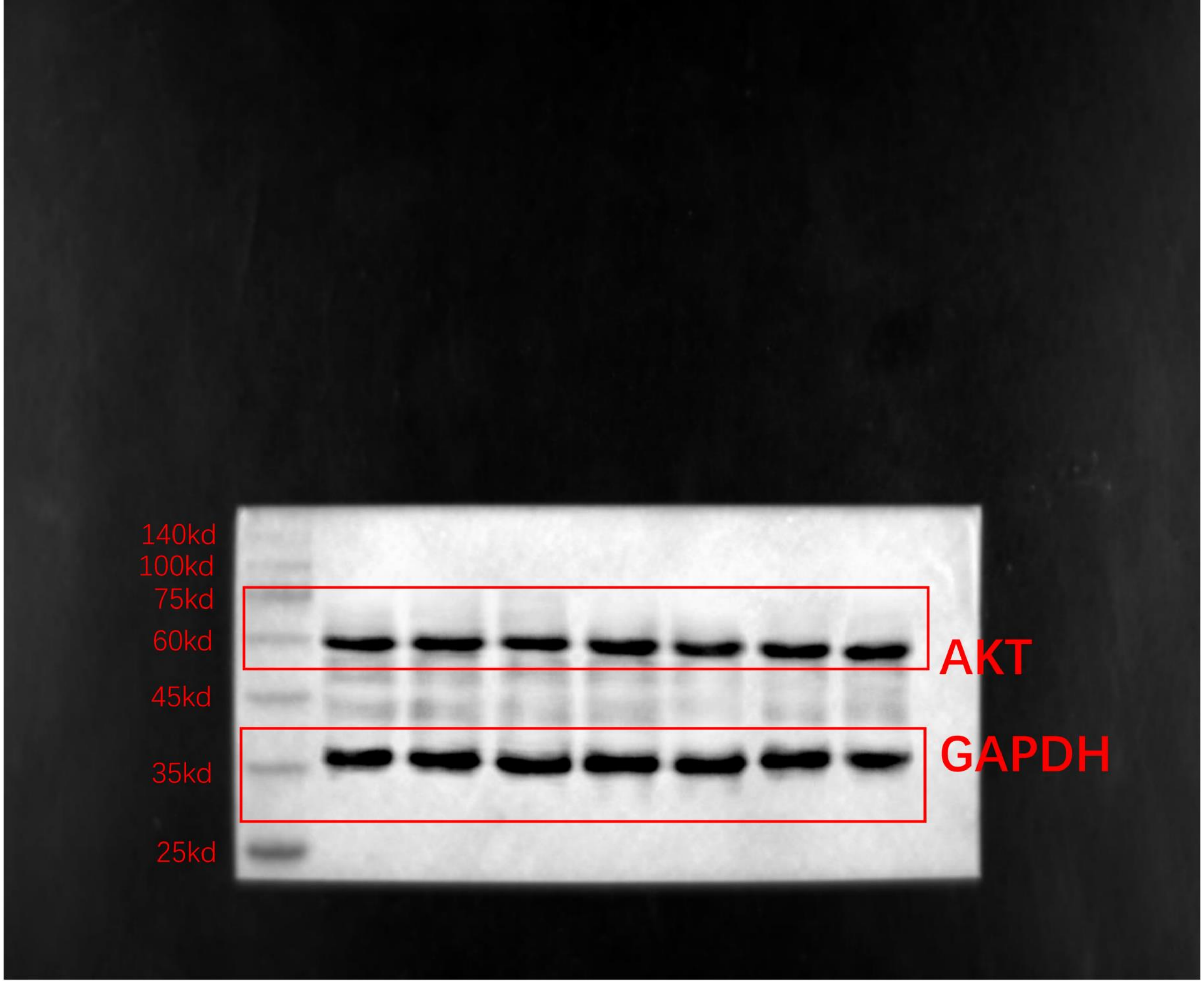

Supplementary figure 1B

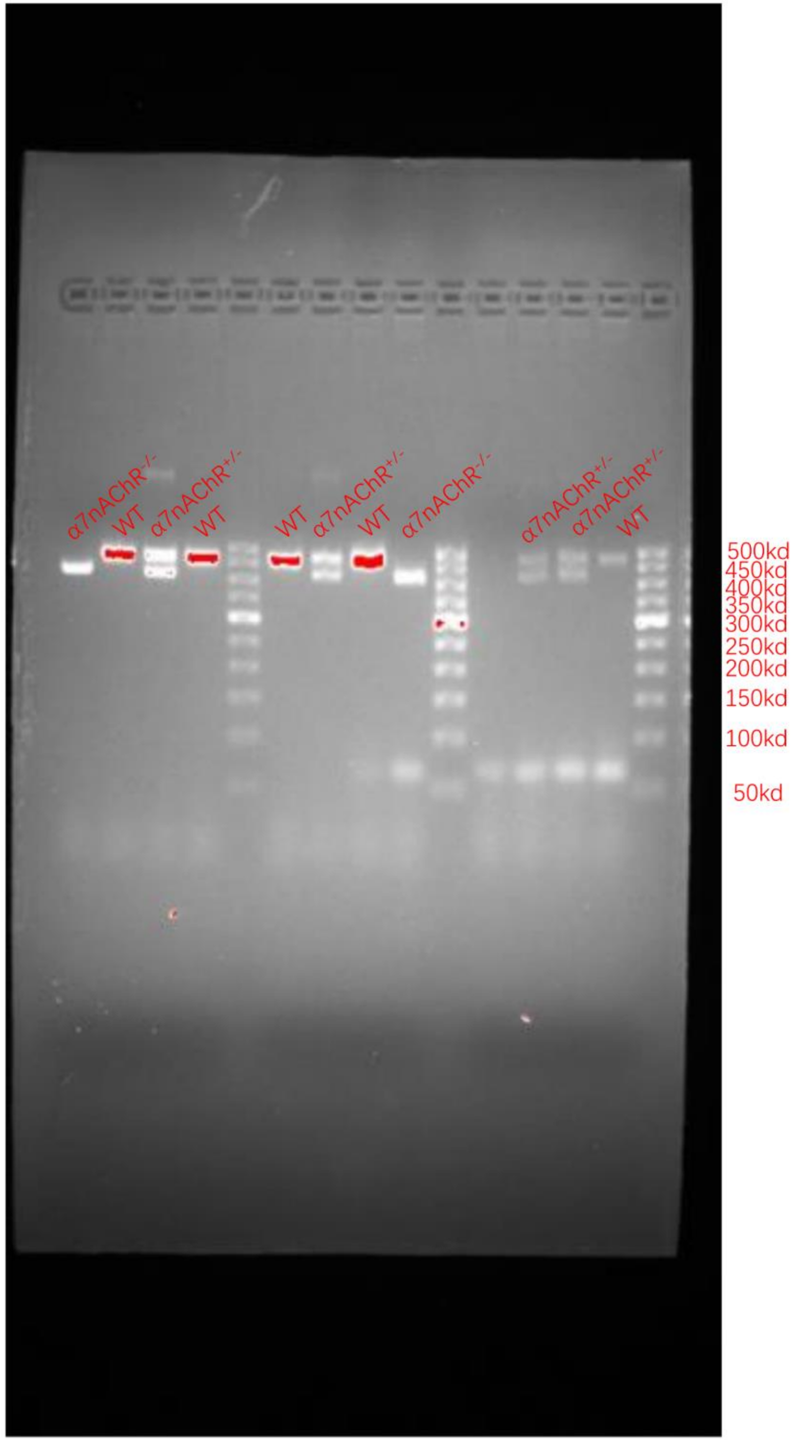

Supplementary figure 1C

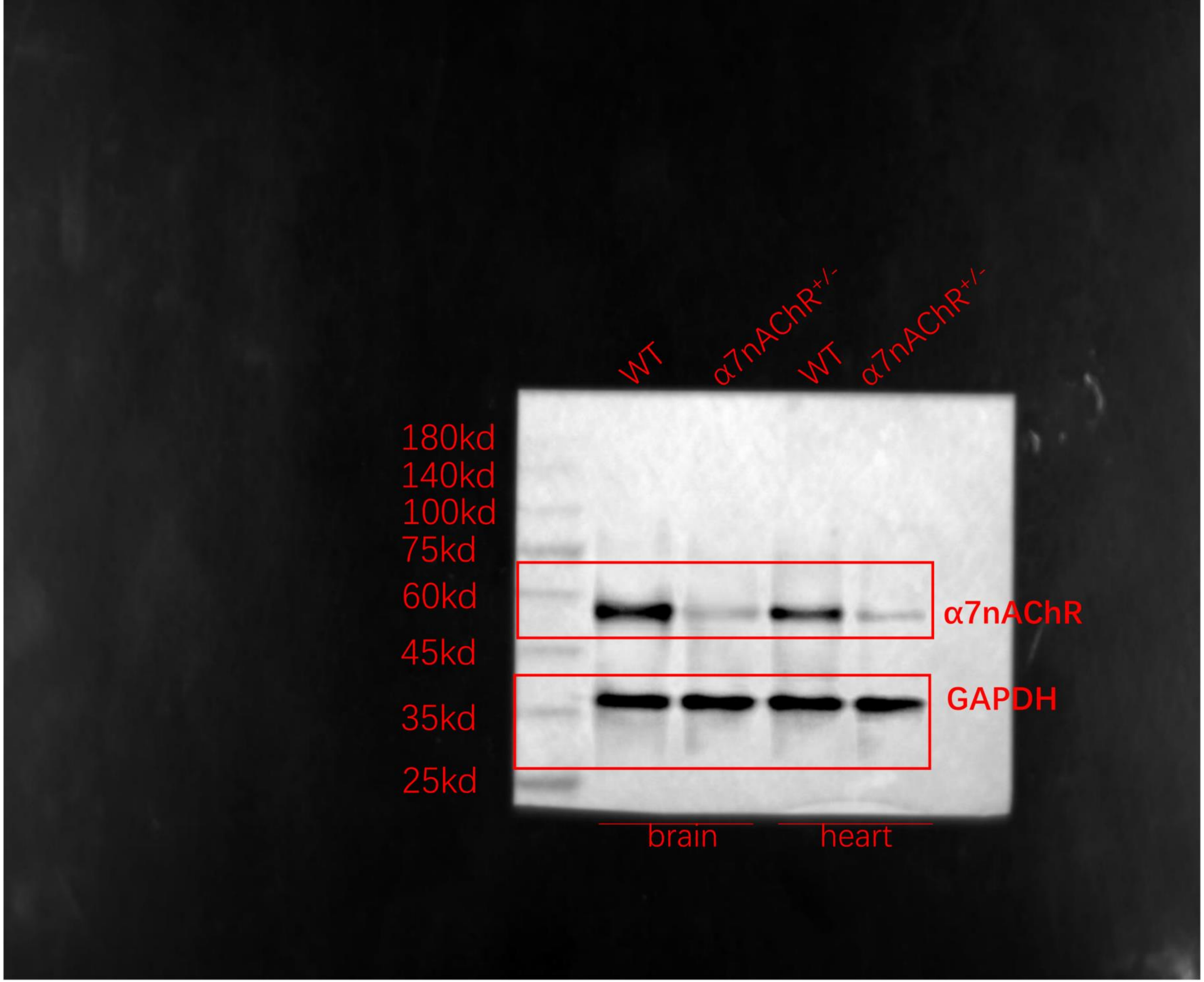

Supplementary figure 1F

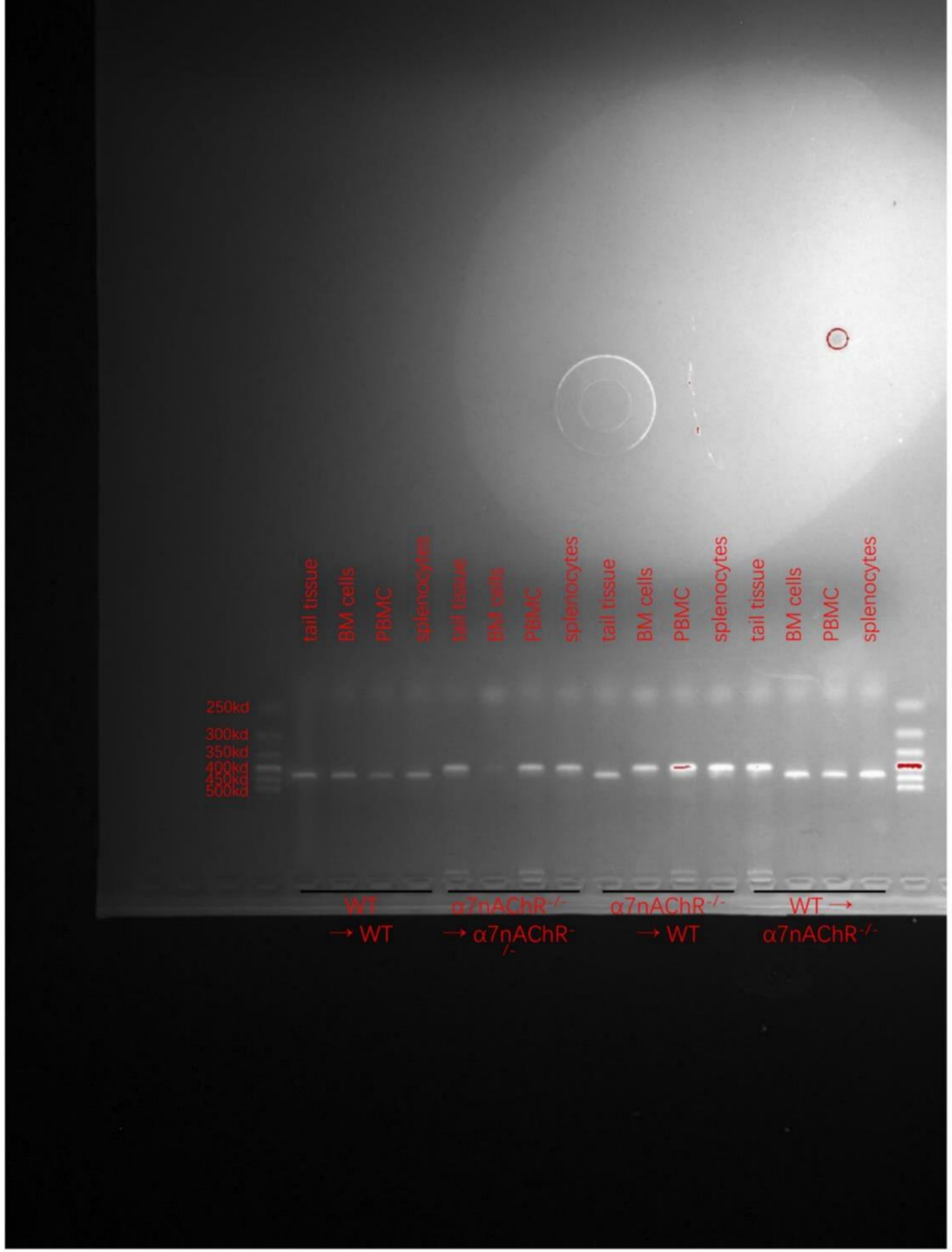

Supplementary figure 4D

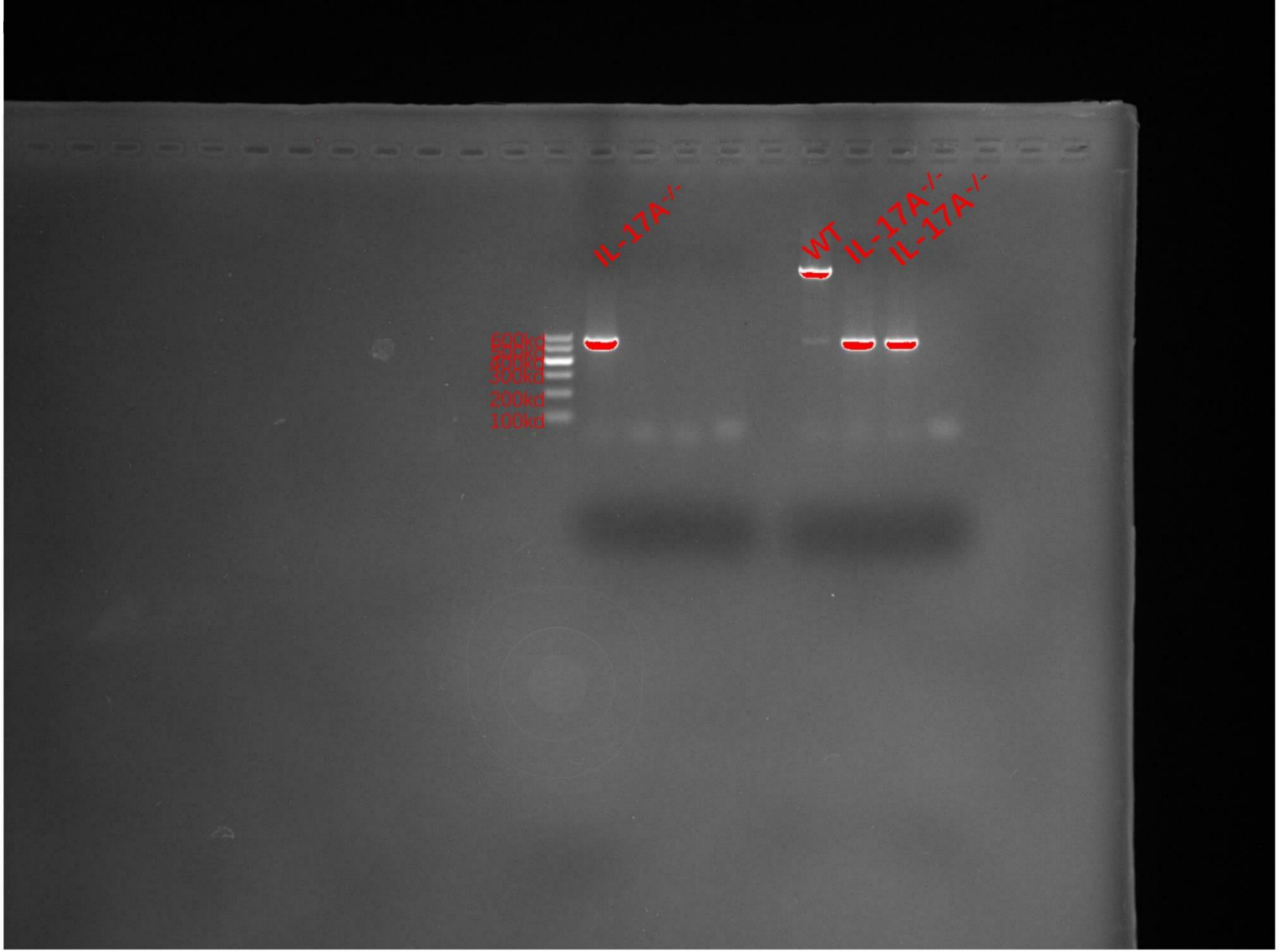

Supplement: Unedited blot and gel images [file jciinsight-10-189323-s149.pdf]
